# Supplementary material for: The signature of competition in ecomorphological traits across the avian radiation
Source: Proc Biol Sci. 2020 Nov 11;287(1938):20201585. doi: 10.1098/rspb.2020.1585 (PMC7735287; doi:10.1098/rspb.2020.1585)
Supplement: Supplementary Material for The signature of competition in ecomorphological traits across the avian radiation [file rspb20201585supp1.docx]

**Supplementary materials for: The signature of competition in ecomorphological traits across the avian radiation**

Authors:

Chira AM, Cooney CR, Bright JA, Capp EJR, Hughes EC, Moody CJA, Nouri LO, Varley ZK, and Thomas, GH

This document contains:

- supplementary information on the quantification of biogeographic reconstructions
- supplementary information on the quantification of phylogenetic uncertainty estimates using the posterior distribution of trees
- Tables S1 – S8

**Models with biogeographic reconstructions**

For each avian clade, we used breeding ranges provided by Birdlife (BirdLife International and Handbook of the Birds of the World (2016) Bird species distribution maps of the world. Version 6.0. Available at http://datazone.birdlife.org/species/requestdis) to first build a matrix which records the presence or absence of each species across the world’s grid cells. We used this presence-absence matrix to build a matrix of co-existence indices for all pairs of species within the focal clade (i.e. a matrix with elements of 1 if the species co-occur anywhere within their ranges, and 0 for the reverse). We then used the clustering algorithm implemented in upgma(), in the R package phangorn to identify sets of species that broadly overlap geographically. The function takes a distance matrix; in our case, the distance between any two species is represented in the distance matrix by d = 1 – species’ co-existence index. This way, a pair of species is separated by a distance of 0 if they co-exist, and a distance of 1 if their ranges do not overlap. The clustering algorithm returns a tree, in which species are separated by edges with lengths between 0 and 0.5. We used the edge lengths to define sets (or clusters) of species that broadly overlap. To avoid a scenario of large numbers of clusters per clade that could lead to unreliable biogeography reconstructions, we defined geographic clusters as groups of species that are separated by distances greater than 0.3 (rather than 0) in the cluster tree, and further, we excluded clusters with less than five species. Each cluster was given a geographic cluster index. We used these indices as an equivalent for discrete characters and used make.simmap() in the R package phytools to build stochastic maps that estimate the biogeography history of the focal clade. Similar to the ecoguild analyses, the geographic object could not be built for all clades. In total, we summarize patterns of trait evolution in 52 out of the 59 clades reported in the main analyses.

**Phylogenetic uncertainty estimates using the posterior distribution of trees**

# We ran our analyses using a maximum clade credibility (MCC) tree for each clade. For each clade, we also quantified the distance between the MCC tree and all trees in the posterior using the function treedist() in the R package *phangorn*. The algorithm calculates four distance metrics: the Robinson-Foulds distance, the branch score difference, the path and weighted path differences. For each clade, we averaged the distance metrics across the posterior to obtain four alternative metrics of phylogenetic dissimilarity between the consensus tree and the posterior. Further, we also used the average across the 75% quantile (to account for the more extreme dissimilarities). We used these metrics as measures of phylogenetic uncertainty and use a phylogenetic logistic regression to test for associations between each of these metrics and a signal for competition within clades.

**Table S1.** Model support (proportion of times each model is chosen as best i.e. smallest AICc values) across clades when modelling the evolution of various ecomorphological traits. The models considered are: Brownian motion (BM), Ornstein-Uhlenbeck (OU), linear (TDlin) and exponential (TDexp) time-dependent, linear (DDlin) and exponential (DDexp) diversity-dependent, and the matching competition (MC). Results summarized across (a) avian orders and super-families, (b) analyses incorporating the biogeographic history of clades, and (c) finer macroevolutionary scales.

| Model | Beak shape PC1 | | Beak shape PC2 | | Beak shape PC3 | | Beak shape PC4 | | Beak size | | Body mass | |  |
| --- | --- | --- | --- | --- | --- | --- | --- | --- | --- | --- | --- | --- | --- |
| a) Orders and super-families (59 clades) | | | | | | | | | | | | |  |
| BM | 0.32 | | 0.10 | | 0.15 | | 0.07 | | 0.12 | | 0.54 | |  |
| OU | 0.51 | | 0.78 | | 0.73 | | 0.76 | | 0.71 | | 0.24 | |  |
| TDlin | 0.03 | | 0 | | 0 | | 0 | | 0 | | 0.05 | |  |
| TDexp | 0.05 | | 0 | | 0 | | 0 | | 0 | | 0.14 | |  |
| DDlin | 0.03 | | 0 | | 0 | | 0 | | 0.05 | | 0 | |  |
| DDexp | 0.03 | | 0.12 | | 0.12 | | 0.10 | | 0.12 | | 0 | |  |
| MC | 0.02 | | 0 | | 0 | | 0.02 | | 0 | | 0.03 | |  |
| b) Orders and super-families (52 clades) – biogeographic analyses | | | | | | | | | | | | |  |
| BM | 0.37 | | 0.12 | | 0.21 | | 0.08 | | 0.19 | | 0.56 | |  |
| OU | 0.40 | | 0.69 | | 0.73 | | 0.77 | | 0.69 | | 0.15 | |  |
| TDlin | 0.02 | | 0 | | 0 | | 0.02 | | 0 | | 0.08 | |  |
| TDexp | 0.06 | | 0 | | 0 | | 0 | | 0 | | 0.12 | |  |
| DDlin | 0.04 | | 0.04 | | 0 | | 0.02 | | 0.06 | | 0 | |  |
| DDexp | 0.10 | | 0.15 | | 0.04 | | 0.10 | | 0.6 | | 0.04 | |  |
| MC | 0.02 | | 0 | | 0.02 | | 0.02 | | 0 | | 0.06 | |  |
| c) Finer macroevolutionary scales (93 clades) | | | | | | | | | | | | | |
| BM | | 0.35 | | 0.12 | | 0.19 | | 0.20 | | 0.17 | | 0.59 | |
| OU | | 0.43 | | 0.75 | | 0.61 | | 0.60 | | 0.63 | | 0.19 | |
| TDlin | | 0.03 | | 0 | | 0.01 | | 0 | | 0.01 | | 0.03 | |
| TDexp | | 0.02 | | 0 | | 0 | | 0.01 | | 0 | | 0.05 | |
| DDlin | | 0.03 | | 0 | | 0 | | 0.03 | | 0.01 | | 0 | |
| DDexp | | 0.12 | | 0.13 | | 0.14 | | 0.13 | | 0.17 | | 0.08 | |
| MC | | 0.01 | | 0 | | 0.04 | | 0.02 | | 0 | | 0.05 | |

**Table S2.** Phylogenetic signal (D statistic) in the signature of competitive interactions. A clade is considered to show a signature of competitive interactions if at least one model with competition is best supported and shows an AICc difference greater than two units from any model assuming lineages evolve independently from each other in either beak shape (PC1, PC2, PC3, PC4), beak size, or body mass. Clades where models with and without competition cannot be distinguished by an AICc difference greater than two are not considered. The D statistic generally takes values between 0 (in which the focal trait, here absence/presence of competition signal, is dispersed as expected under a Brownian motion model, and so shows high phylogenetic signal) and 1 (where the focal trait is dispersed randomly across the phylogeny). Additionally, D statistic values outside these ranges can be obtained if the focal trait is extremely phylogenetically clumped (values <0) or overdispersed (values >1). D statistic values are compared to a Brownian expectation (significant p-values indicate no phylogenetic signal) and a random expectation (significant p-values indicate evidence for phylogenetic signal). Results summarized across avian orders and super-families (a), as well as across finer macroevolutionary scales (b).

| Trait | D estimate | D is different from 0 (Brownian expectation) | Difference from 1 (random expectation) | # Clades with support for competition |
| --- | --- | --- | --- | --- |
| (a) Orders and super-families | | | | |
| Any trait | 1.463 | p = 0.002 | p = 0.886 | 20/58 |
| (b) Finer macroevolutionary scales | | | | |
| Any trait | 0.808 | p = 0.013 | p = 0.213 | 37/91 |

**Table S3.** The relationship between clade age and the signal of competition in avian clades, while accounting for clade species richness. A clade is considered to show a signature of competitive interactions if at least one model with competition is best supported and shows an AICc difference greater than two units from any model assuming lineages evolve independently from each other in either beak shape (PC1, PC2, PC3, PC4), beak size, or body mass. Clades where models with and without competition cannot be distinguished by an AICc difference greater than two are not considered. Results summarized across avian orders and super-families (a), as well as across finer macroevolutionary scales (b).

| Predictor | Coefficient estimate ± SE | p - value |
| --- | --- | --- |
| (a) Orders and super-families, 20/58 clades show support for competition | | |
| Log clade age | -0.824 ± 0.743 | 0.267 |
| Log species richness | -1.019 ± 0.463 | **0.028** |
| (b) Finer macroevolutionary scales, 26/90 clades show support for competition | | |
| Log clade age | 0.668 ± 0.416 | 0.125 |
| Log species richness | -0.778 ± 0.419 | 0.064 |

**Table S4.** The relationship between clade age and the signal of competition in avian clades, while accounting for clade species richness. A clade is considered to show a signature of competitive interactions if at least one model with competition is best supported and shows an AICc difference greater than two units from any model assuming lineages evolve independently from each other in either beak shape (PC1, PC2, PC3, PC4), beak size, or body mass. Clades where models with and without competition cannot be distinguished by an AICc difference greater than two are not considered. Results summarized across avian orders and super-families trees build using species for which genetic data is available.

| Predictor | Coefficient estimate ± SE | p - value |
| --- | --- | --- |
| Orders and super-families, 16/53 clades show support for competition | | |
| Log clade age | -0.662 ± 0.736 | 0.368 |
| Log species richness | -0.708 ± 0.495 | 0.153 |

**Table S5.** Model support (proportion of times each model is chosen as best i.e. smallest AICc values) across clades when modelling the evolution of various ecomorphological traits. The models considered are: Brownian motion (BM), Ornstein-Uhlenbeck (OU), linear (TDlin) and exponential (TDexp) time-dependent, linear (DDlin) and exponential (DDexp) diversity-dependent, and the matching competition (MC). Results summarized across avian orders and super-families trees built using species for which genetic data is available (54 clades).

| Model | BeakShape PC1 | BeakShape PC2 | BeakShape PC3 | BeakShape PC4 | Beak size | Body mass |
| --- | --- | --- | --- | --- | --- | --- |
| BM | 0.30 | 0.13 | 0.07 | 0.07 | 0.13 | 0.56 |
| OU | 0.46 | 0.76 | 0.78 | 0.81 | 0.70 | 0.26 |
| TDlin | 0.06 | 0 | 0.02 | 0.02 | 0 | 0.11 |
| TDexp | 0.06 | 0 | 0.02 | 0 | 0 | 0.07 |
| DDlin | 0.02 | 0 | 0.02 | 0 | 0.02 | 0 |
| DDexp | 0.09 | 0.11 | 0.09 | 0.09 | 0.13 | 0 |
| MC | 0.02 | 0 | 0 | 0 | 0.02 | 0 |

**Table S6.** Phylogenetic signal (D statistic) in the signature of competitive interactions across beak shape (PC1, PC2, PC3, PC4), relative beak size, body mass, or any trait (i.e. a signal of competition in beak shape, size, and/or body mass). A clade is categorized as showing a signature of competitive interactions if at least one model with competition is best supported and shows an AICc difference greater than two units from any model assuming lineages evolve independently from each other. Clades where models with and without competition cannot be distinguished by an AICc difference greater than two are not considered. The D statistic generally takes values between 0 (in which the focal trait, here absence/presence of competition signal, is dispersed as expected under a Brownian motion model, and so shows high phylogenetic signal) and 1 (where the focal trait is dispersed randomly across the phylogeny). Additionally, D statistic values outside these ranges can be obtained if the focal trait is extremely phylogenetically clumped (values <0) or overdispersed (values >1). D statistic values are compared to a Brownian expectation (significant p-values indicate no phylogenetic signal) and a random expectation (significant p-values indicate evidence for phylogenetic signal). Results summarized across avian orders and super-families trees build using species for which genetic data is available.

| Trait | D estimate | D is different from 0 (Brownian expectation) | Difference from 1 (random expectation) | # Clades with support for competition |
| --- | --- | --- | --- | --- |
| Any trait | 2.022 | p = 0 | 0.997 | 16/53 |

**Table S7.** Model support (proportion of times each model is chosen as best i.e. smallest AICc values) across clades when modelling the evolution of avian beak shape using a phylogenetic principal component analysis. The models considered are: Brownian motion (BM), Ornstein-Uhlenbeck (OU), linear (TDlin) and exponential (TDexp) time-dependent, linear (DDlin) and exponential (DDexp) diversity-dependent, and the matching competition (MC). Results summarized across 59 avian orders and super-families.

| Model | BeakShape pPC1 | BeakShape pPC2 | BeakShape pPC3 | BeakShape pPC4 |
| --- | --- | --- | --- | --- |
| BM | 0.10 | 0.27 | 0.31 | 0.08 |
| OU | 0.76 | 0.49 | 0.54 | 0.76 |
| TDlin | 0.02 | 0.12 | 0 | 0 |
| TDexp | 0 | 0.03 | 0 | 0 |
| DDlin | 0 | 0 | 0.05 | 0.02 |
| DDexp | 0.12 | 0.07 | 0.10 | 0.10 |
| MC | 0 | 0.02 | 0 | 0.03 |

| **Table S8.** The relationship between measures of phylogenetic uncertainty (dissimilarity between the MCC tree and the posterior trees) and the signal of competition in avian clades. A clade is considered to show a signature of competitive interactions if at least one model with competition is best supported and shows an AICc difference greater than two units from any model assuming lineages evolve independently from each other in either beak shape (PC1, PC2, PC3, PC4), beak size, or body mass. Clades where models with and without competition cannot be distinguished by an AICc difference greater than two are not considered. Results summarized across avian orders and super-families. For each clade, dissimilarities between the consensus tree and the posterior are (a) averaged, or (b) averaged across the 75% quantile of the distribution of distances. Note that the path differences show significant negative effects on the prevalence of competition signal (i.e. higher odds of competition signal for smaller levels of uncertainty), but the p- values are very close to the threshold of significance. Further, if false positives in competition signal are of concern, the expectation would be of positive coefficient estimates in these models. | | |
| --- | --- | --- |
| Uncertainty metric | Coefficient estimate ± SE | p – value |
| (a) Average distances |  |  |
| Robinson-Foulds distance | -0.150 ± 0.089 | 0.091 |
| Branch score difference | -0.007 ± 0.008 | 0.287 |
| Path differences | -0.693 ± 0.350 | **0.047** |
| Weighted path differences | -0.693 ± 0.350 | **0.047** |
| (b) Average distances across the 75% quantile | | |
| Robinson-Foulds distance | -0.117 ± 0.089 | 0.187 |
| Branch score difference | -0.015 ± 0.009 | 0.105 |
| Path differences | -0.741± 0.364 | **0.042** |
| Weighted path differences | -0.741± 0.364 | **0.042** |
