# Supplementary material for: The signature of competition in ecomorphological traits across the avian radiation
Source: Proc Biol Sci. 2020 Nov 11;287(1938):20201585. doi: 10.1098/rspb.2020.1585 (PMC7735287; doi:10.1098/rspb.2020.1585)
Supplement: Supplementary figures for The signature of competition in ecomorphological traits across the avian radiation [file rspb20201585supp2.pdf]

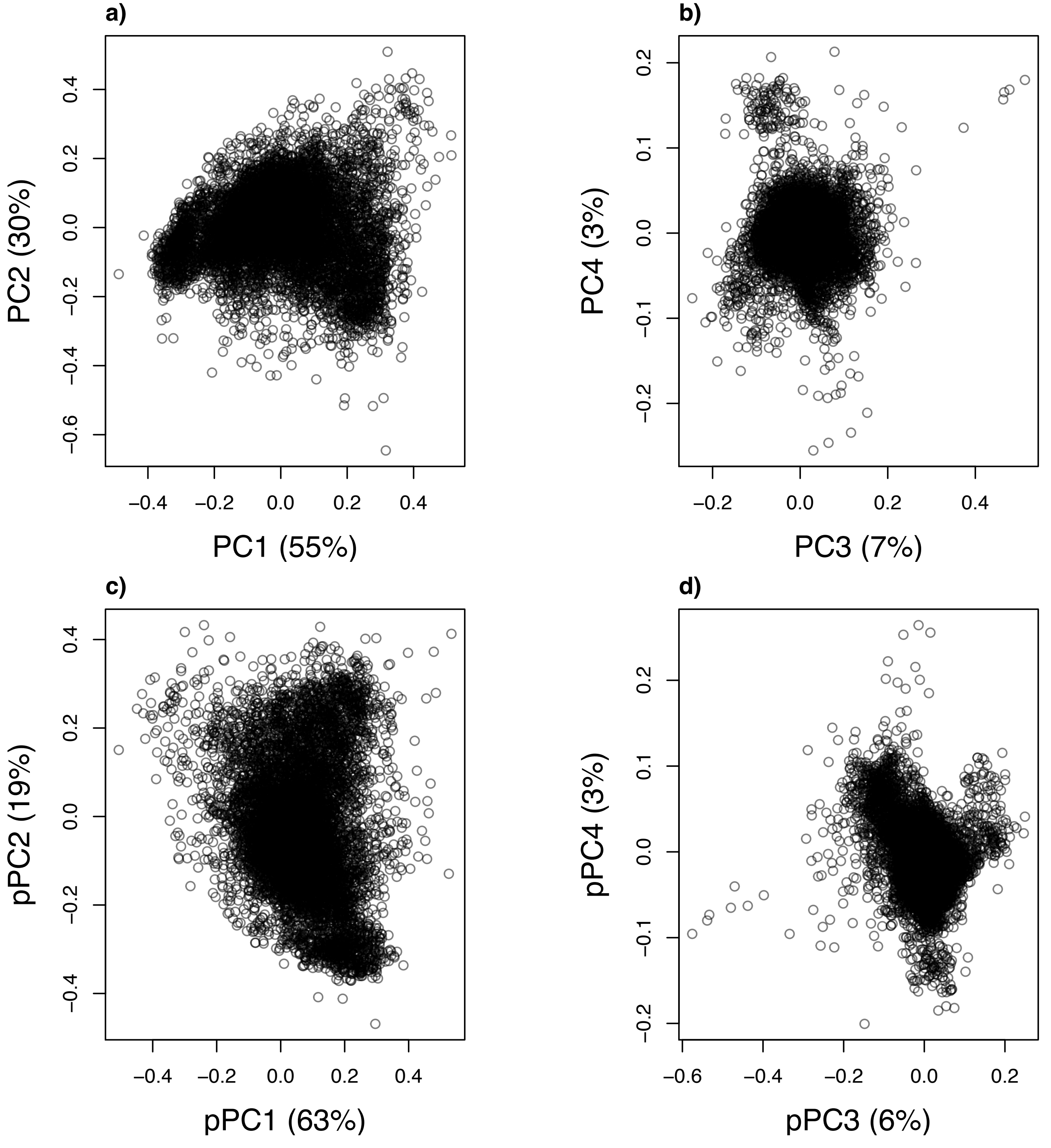

Figure S1a. Avian beak shape morphospace illustrated as a scatterplot of the first four (ab) PC, and (cd) phylogenetic PC axes of variation. The percentage of variance explained by each PC and phylogenetic PC axis is shown in brackets.

Figure S1b (Overleaf). Avian ecoguild morphospace for various orders and super-families. Each species is assigned a diet and foraging strategy category, and a PCA for categorical data is performed for each clade. The first two PC axes of variation are used to cluster species into four ecoguilds, correspondent to the four quadrants of the scatterplot regressing the first two PC axes of variation (percentage of variation explained by each dimension is shown in brackets). Each point represents one or a cluster of species with similar values for foraging strategy and diet category. The original EltonTraits categories that shape the ecoguild space are shown, and their contribution is marked by the colour scheme.

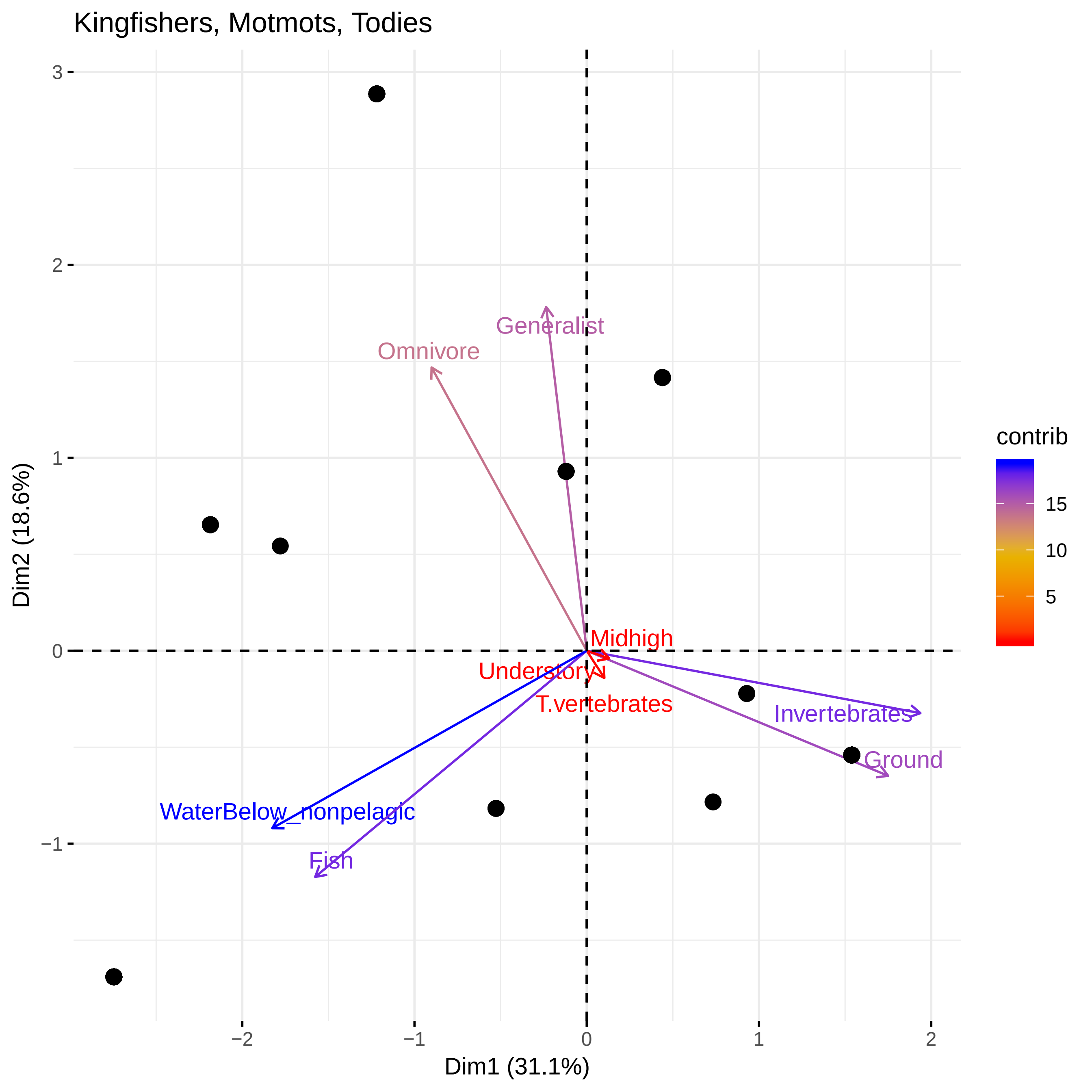
Kingfishers, Motmots, Todies
Generalist
Omnivore
Midhigh
Understory
T.vertebrates
Invertebrates
Ground
WaterBelow_nonpelagic
Fish
contrib
15
10
5
3
2
1
0
−1
−2
−1
0
1
2
Dim1 (31.1%)
Dim2 (18.6%)

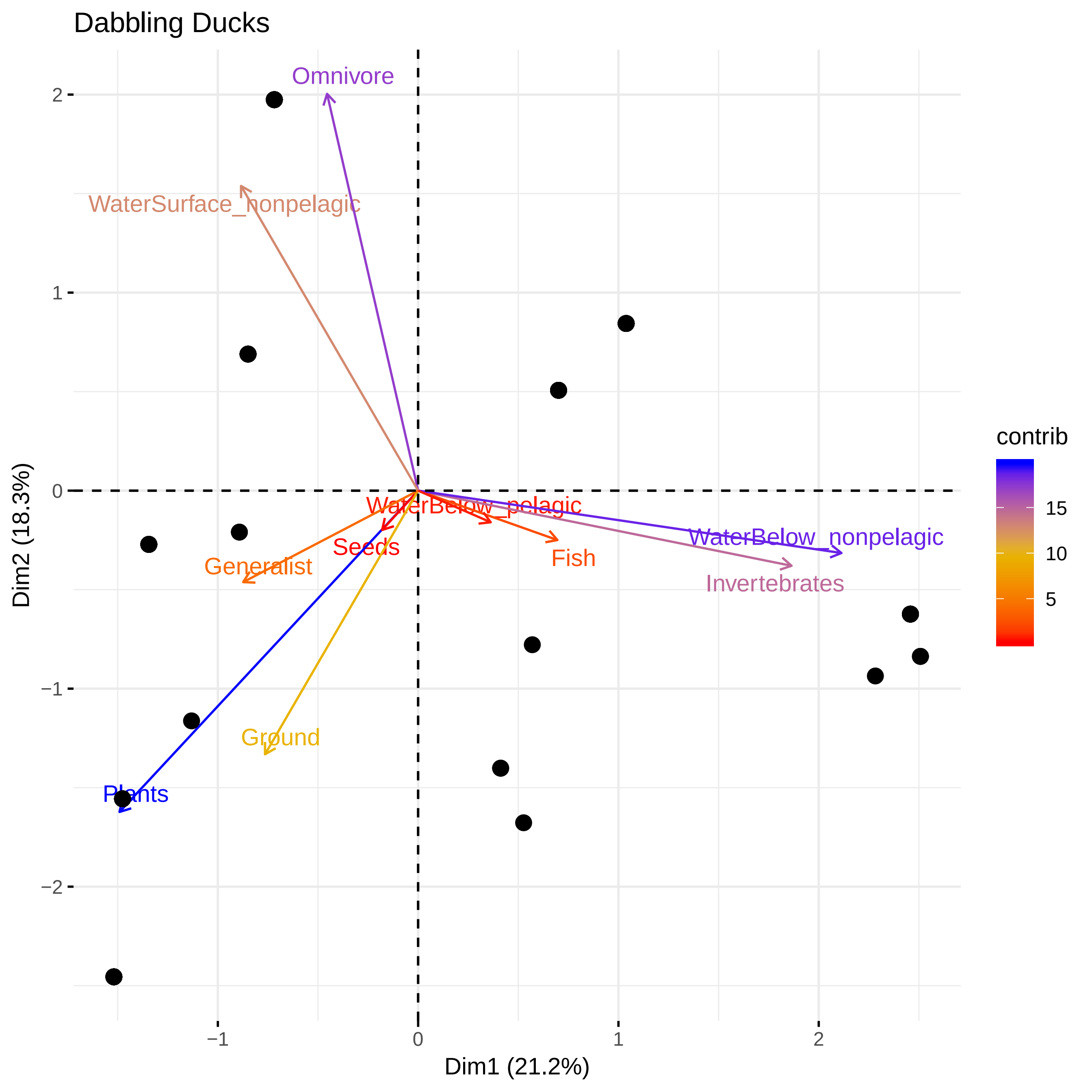

Dabbling Ducks
Omnivore
WaterSurface_nonpelagic
WaterBelow_pelagic
Seeds
Generalist
Fish
WaterBelow_nonpelagic
Invertebrates
Ground
Plants
contrib
15
10
5
2
1
0
−1
−2
Dim2 (18.3%)
Dim1 (21.2%)

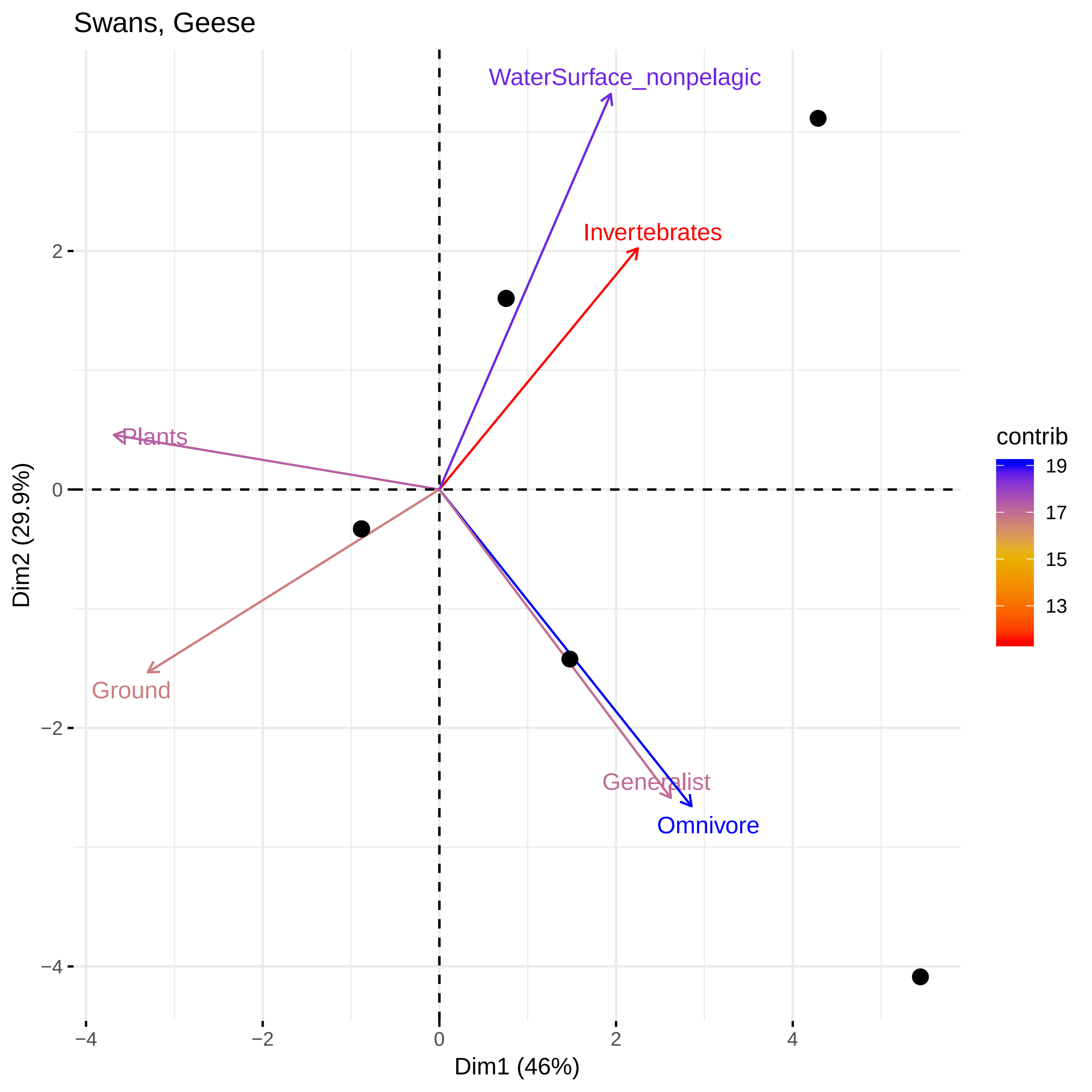
Swans, Geese
WaterSurface_nonpelagic
Invertebrates
Plants
Ground
Generalist
Omnivore
Dim1 (46%)
Dim2 (29.9%)
contrib
19
17
15
13
2
0
−2
−4
−4
−2
0
2
4

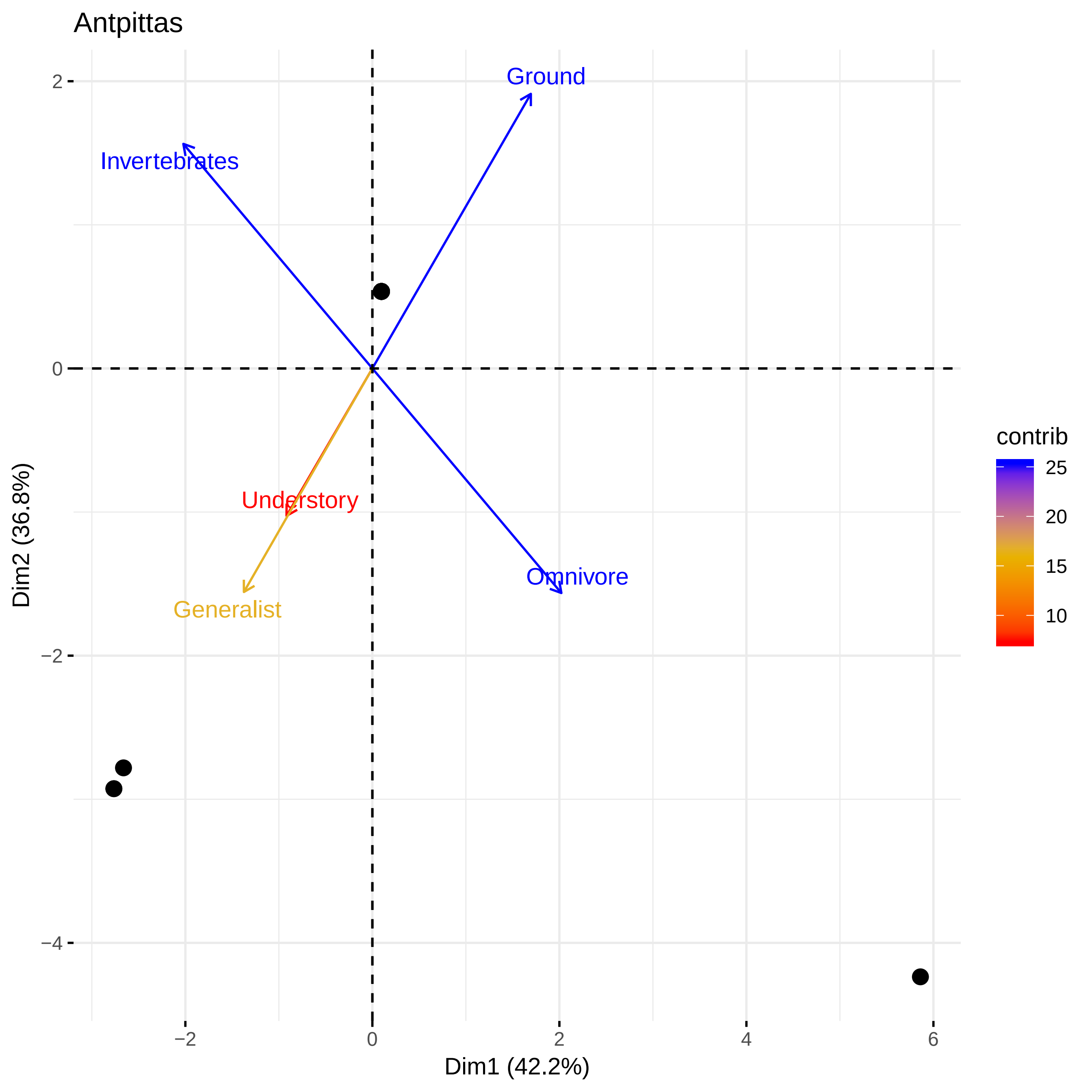

Antpittas
Ground
Invertebrates
Understory
Omnivore
Generalist
contrib
25
20
15
10
2
0
−2
−4
−2
0
2
4
6
Dim1 (42.2%)
Dim2 (36.8%)

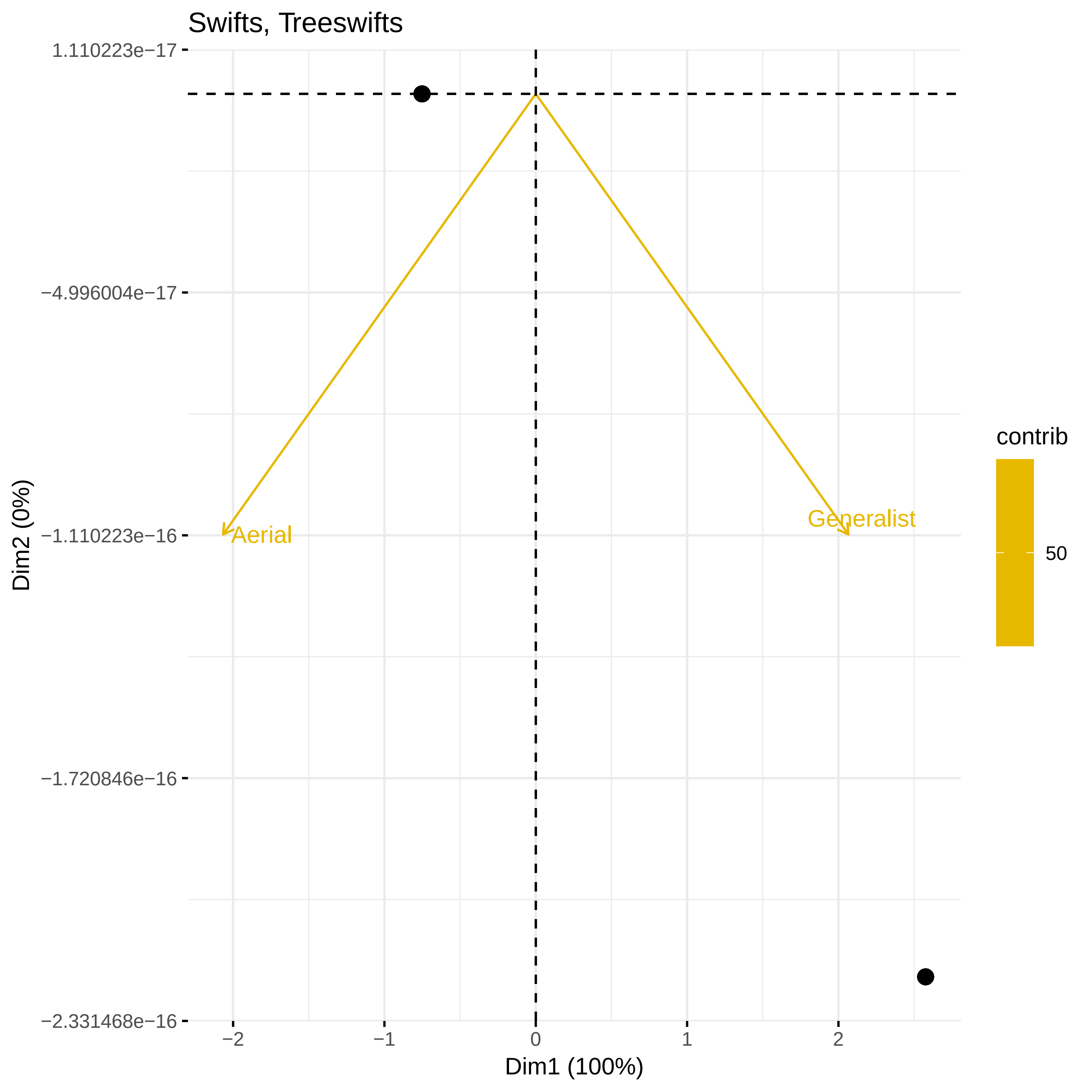

Swifts, Treeswifts
1.110223e−17
−4.996004e−17
−1.110223e−16
−1.720846e−16
−2.331468e−16
Dim2 (0%)
−2
−1
0
1
2
Dim1 (100%)
Aerial
Generalist
contrib
50

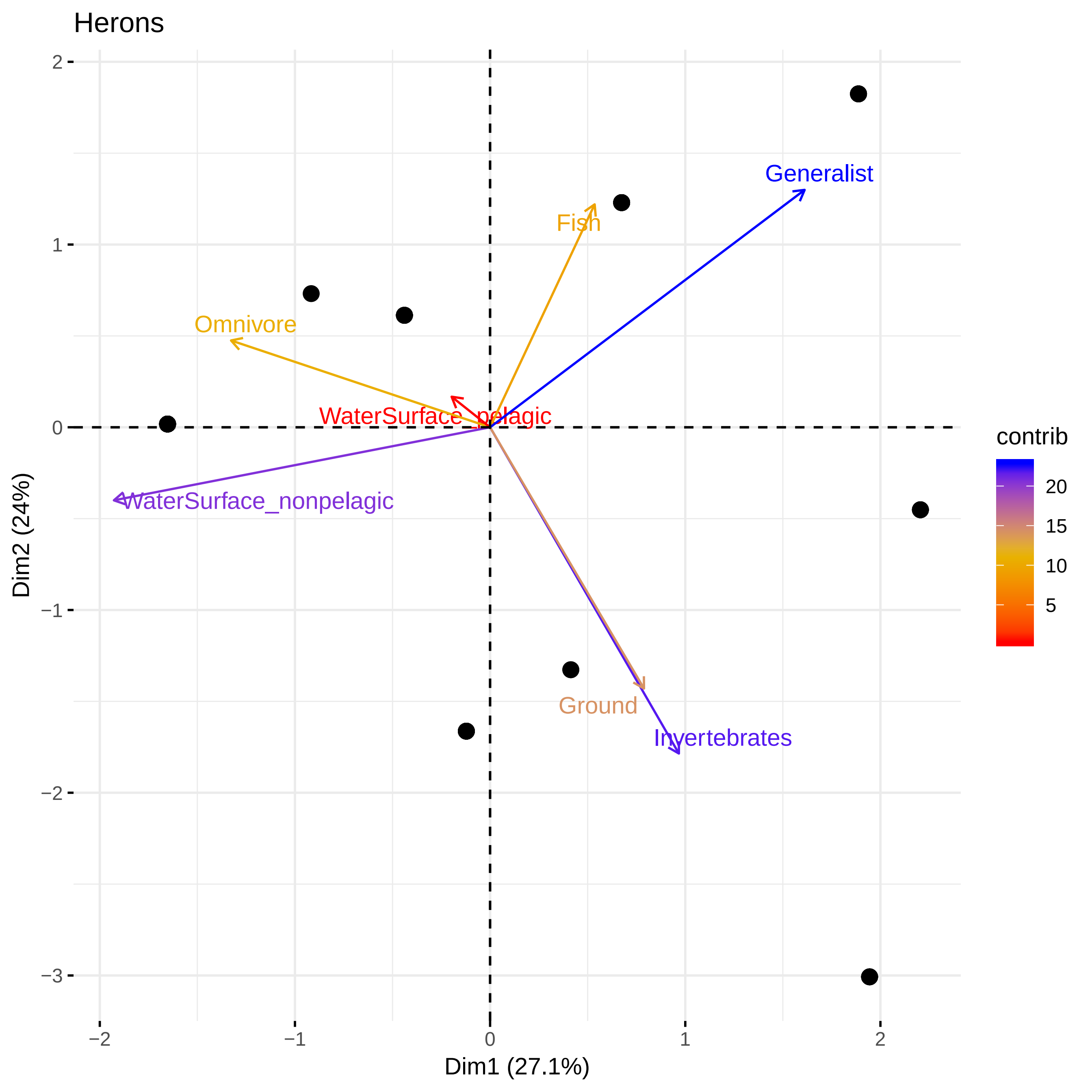
Herons
Generalist
Fish
Omnivore
WaterSurface_pelagic
WaterSurface_nonpelagic
Ground
Invertebrates
contrib
20
15
10
5
Dim1 (27.1%)
Dim2 (24%)
−2
−1
0
1
2
−3

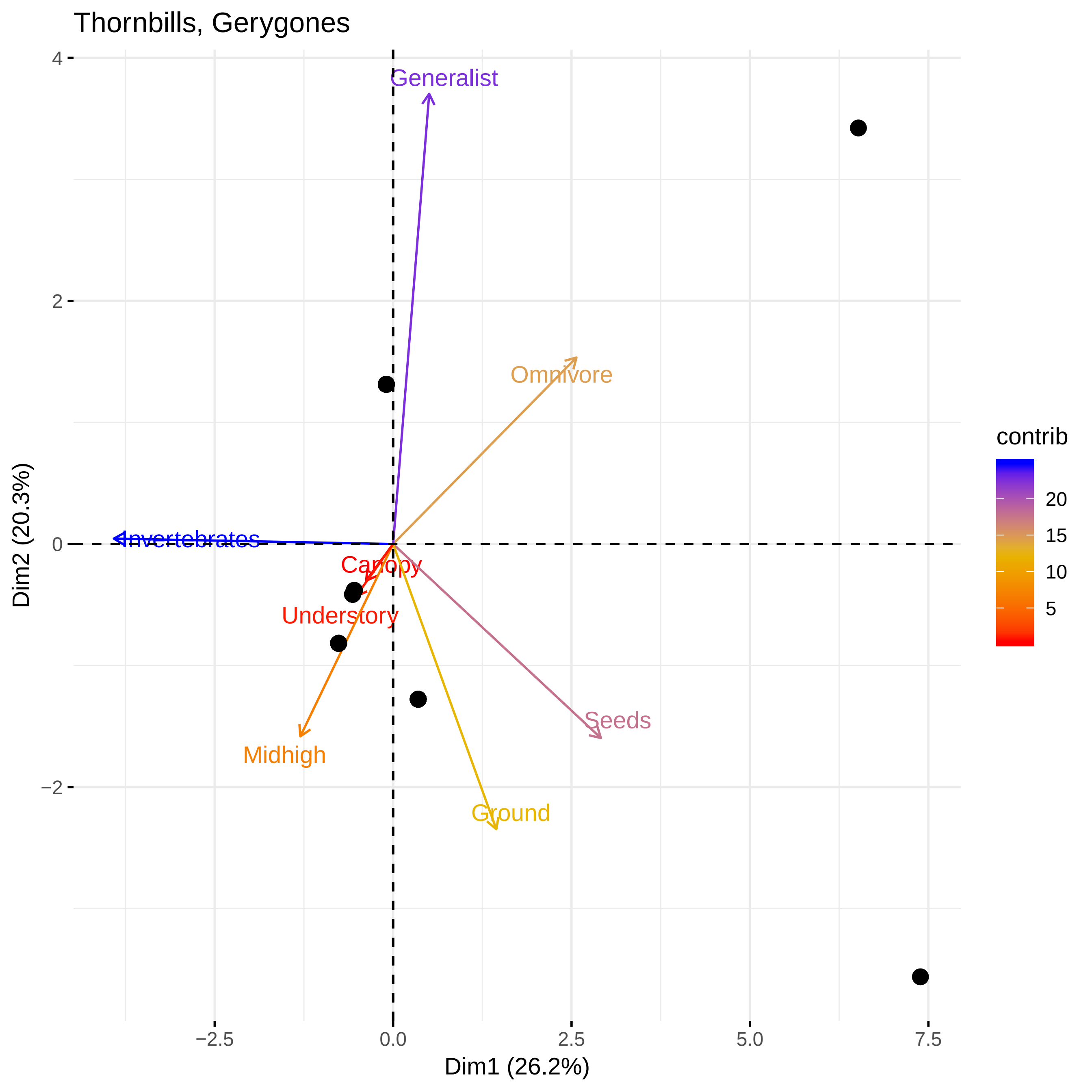

Thornbills, Gerygones
Generalist
Omnivore
Invertebrates
Canopy
Understory
Midhigh
Seeds
Ground
contrib
20
15
10
5
4
2
0
−2
−2.5
0.0
2.5
5.0
7.5
Dim1 (26.2%)
Dim2 (20.3%)

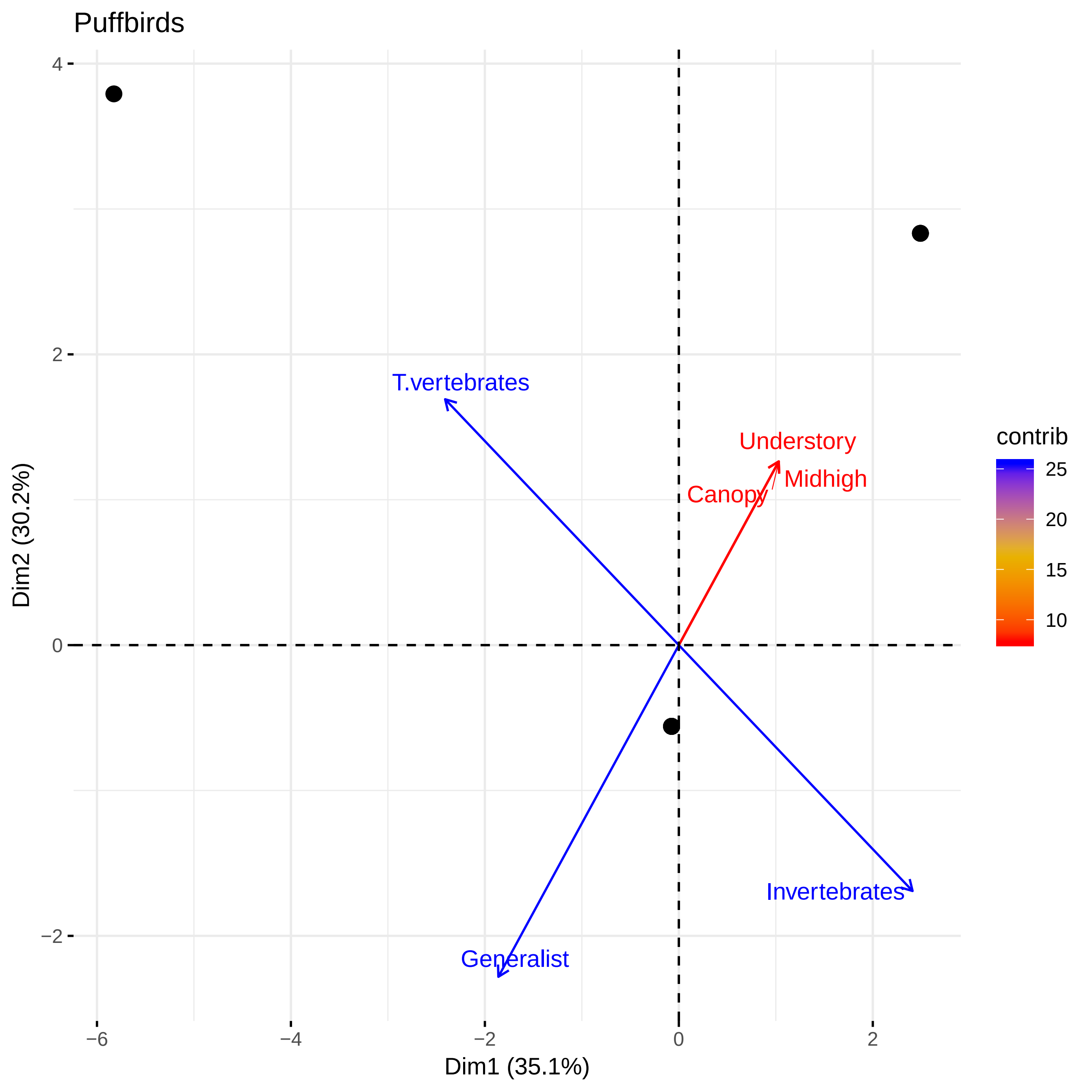
Puffbirds
T.vertebrates
Understory
Midhigh
Canopy
Invertebrates
Generalist
contrib
25
20
15
10
4
2
0
−2
−6
−4
−2
0
2
Dim1 (35.1%)
Dim2 (30.2%)

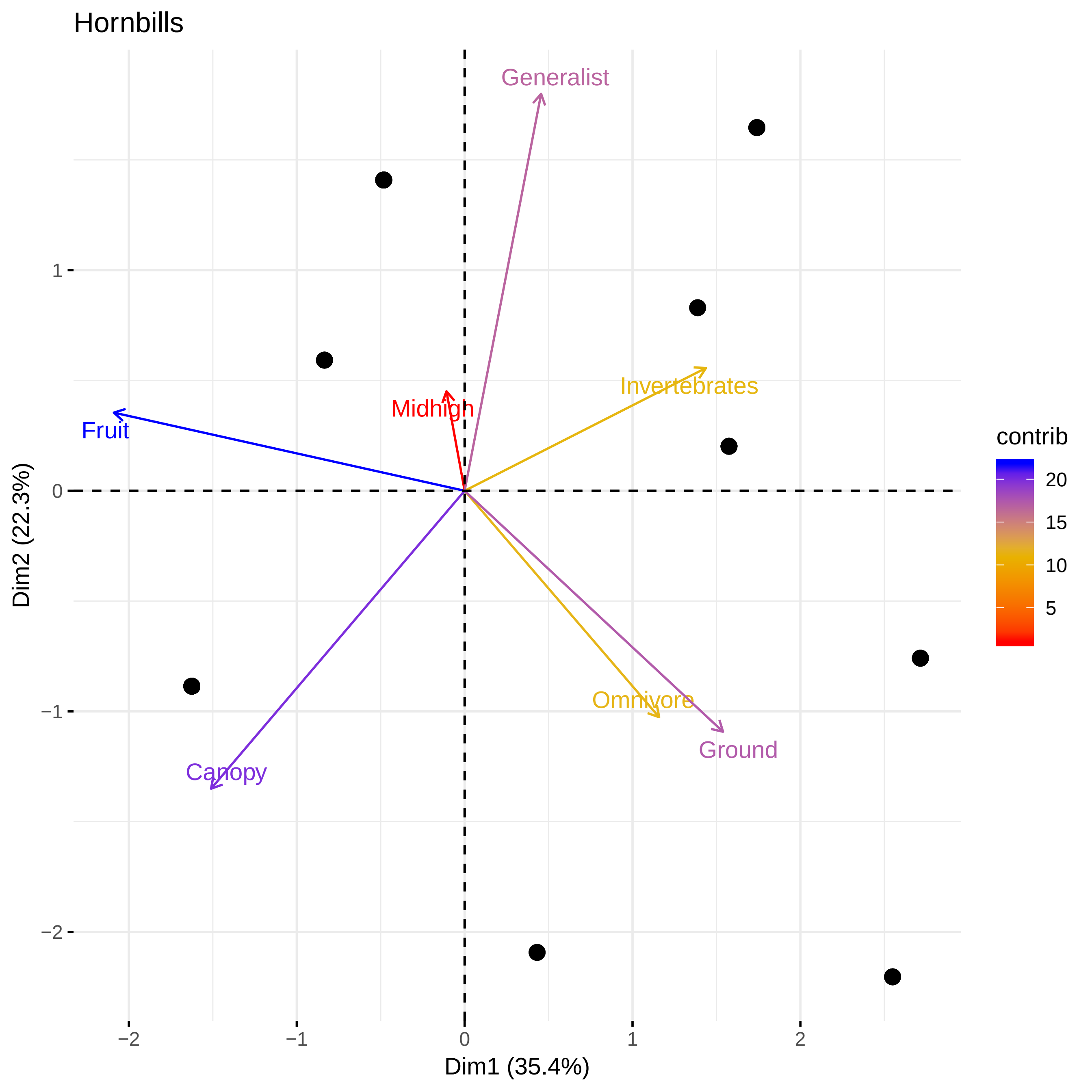

Hornbills
Generalist
Invertebrates
Midhigh
Fruit
Omnivore
Ground
Canopy
contrib
20
15
10
5
1
0
−1
−2
−2
−1
0
1
2
Dim1 (35.4%)
Dim2 (22.3%)

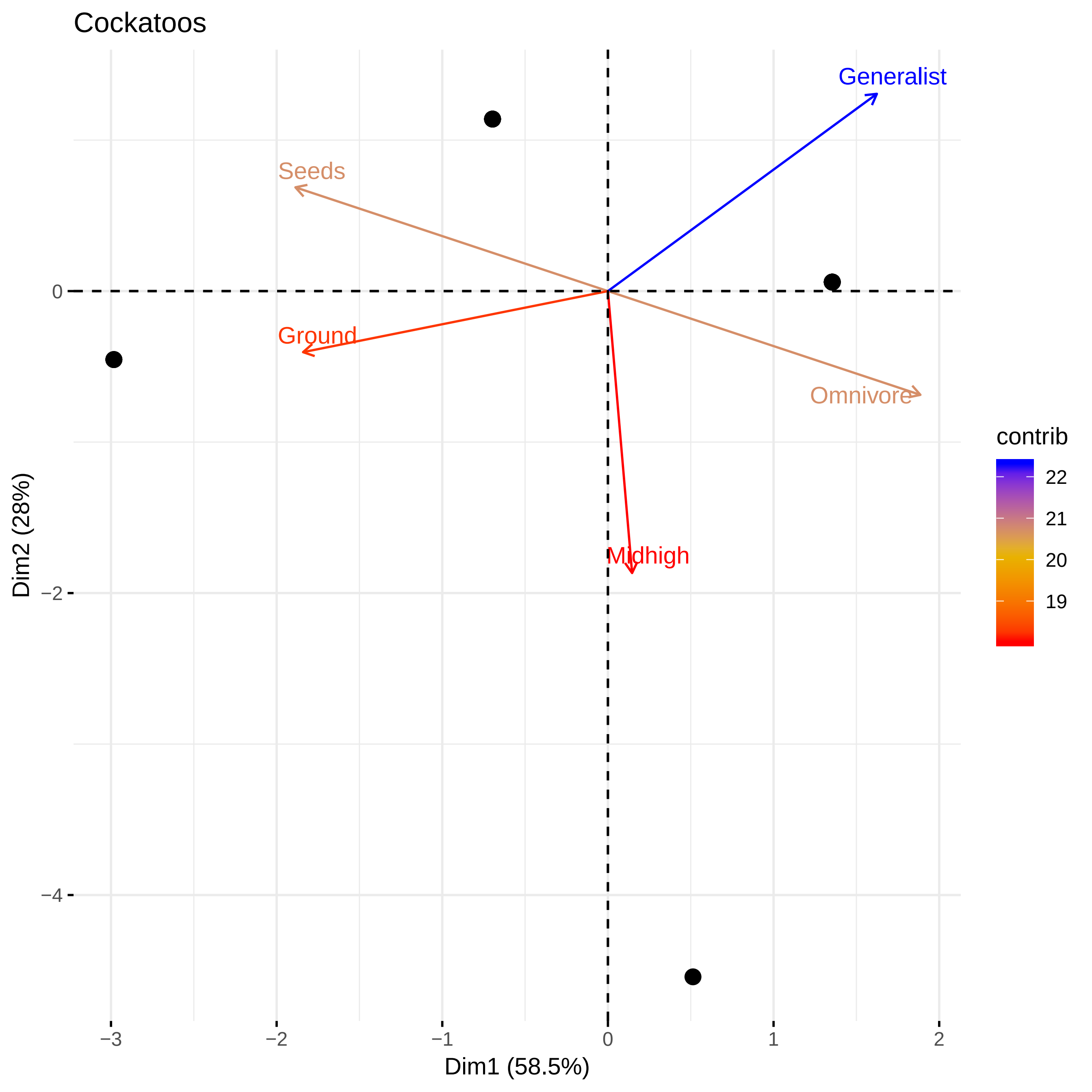

Cockatoos
Generalist
Seeds
Ground
Omnivore
Midhigh
contrib
22
21
20
19
0
−2
−4
−3
−2
−1
0
1
2
Dim1 (58.5%)
Dim2 (28%)

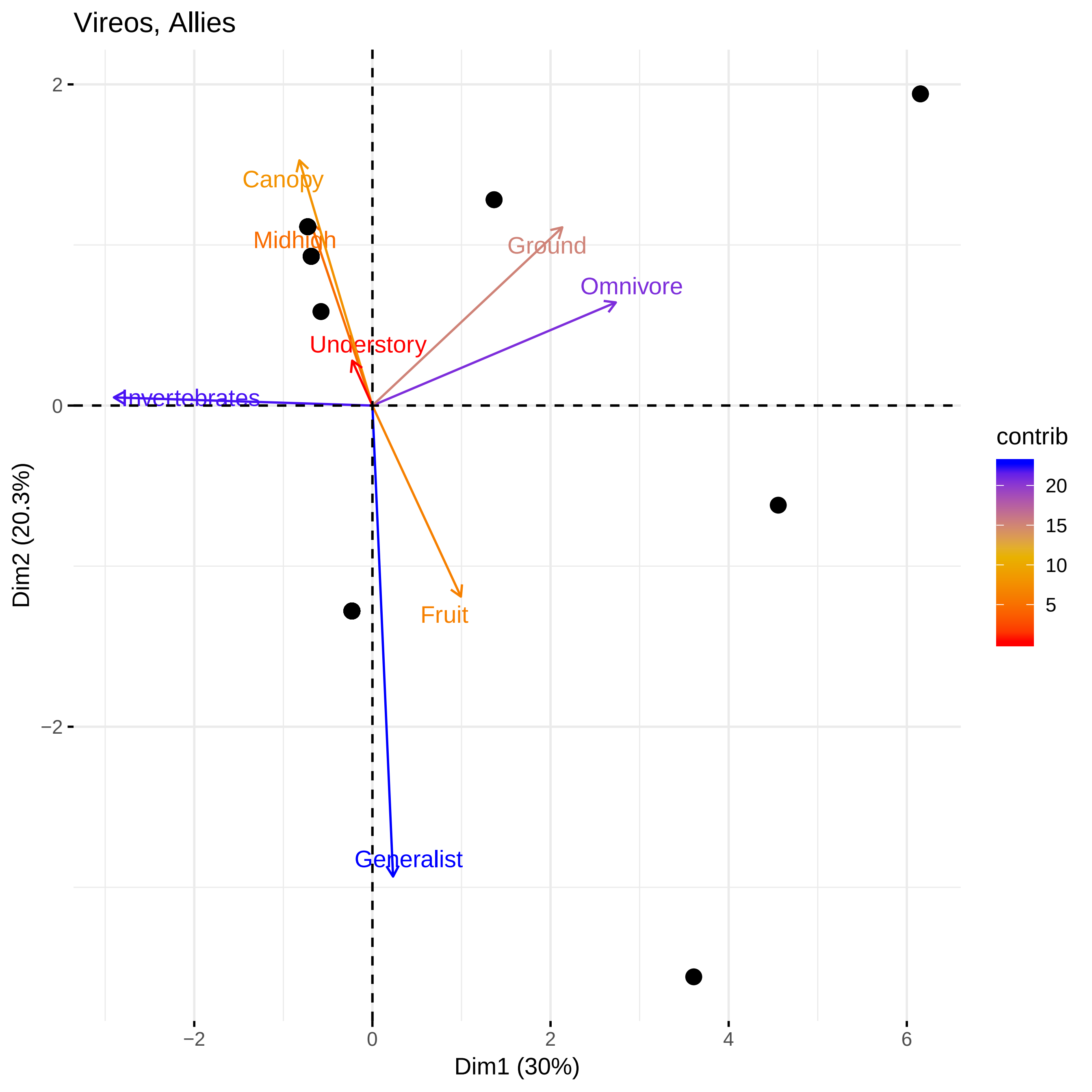
Vireos, Allies
Canopy
Midhigh
Ground
Omnivore
Understory
Invertebrates
Fruit
Generalist
contrib
20
15
10
5
2
0
−2
−2
0
2
4
6
Dim1 (30%)
Dim2 (20.3%)

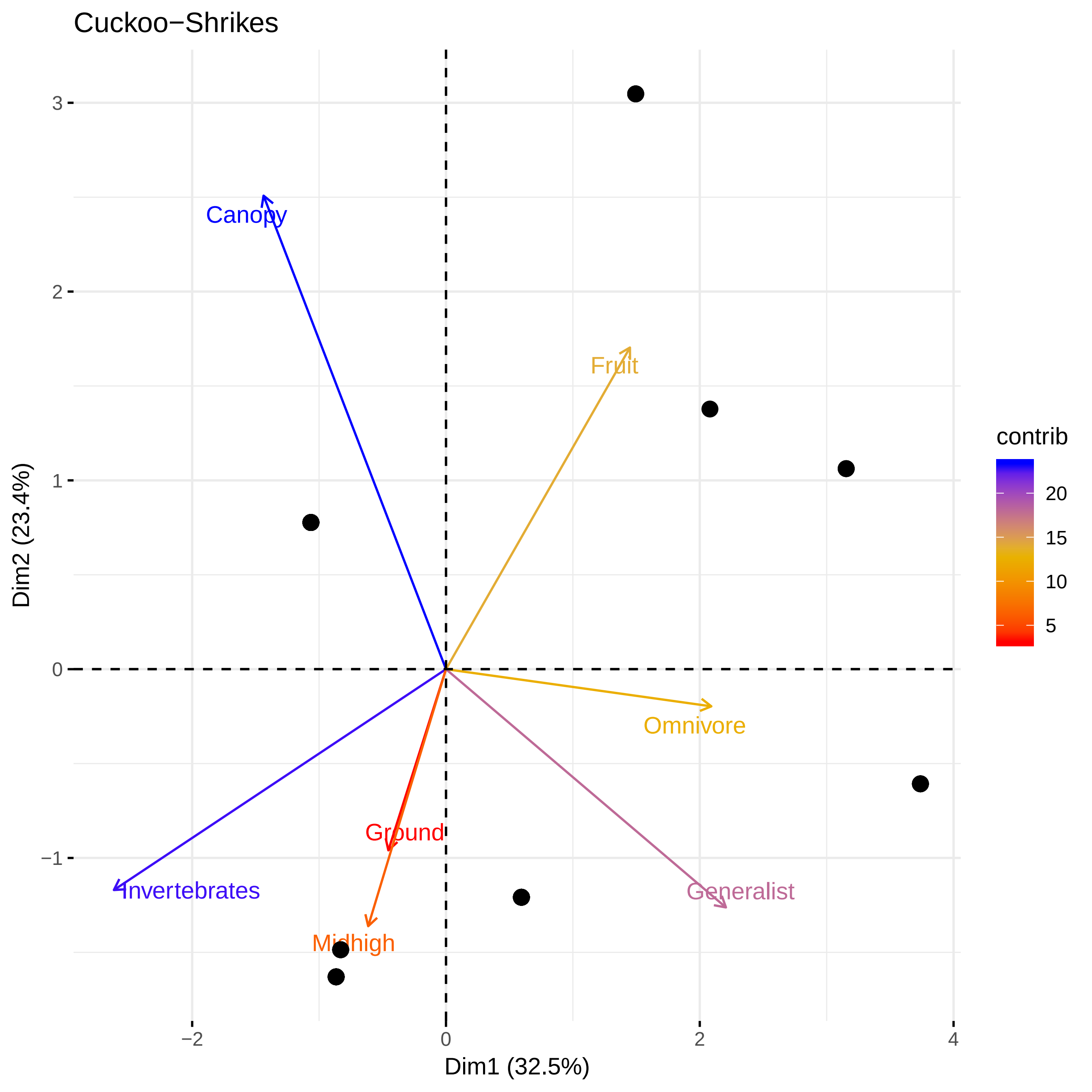
Cuckoo−Shrikes
Canopy
Fruit
Omnivore
Ground
Invertebrates
Generalist
Midhigh
contrib
20
15
10
5
Dim2 (23.4%)
Dim1 (32.5%)
3
2
1
0
−1
−2
0
2
4

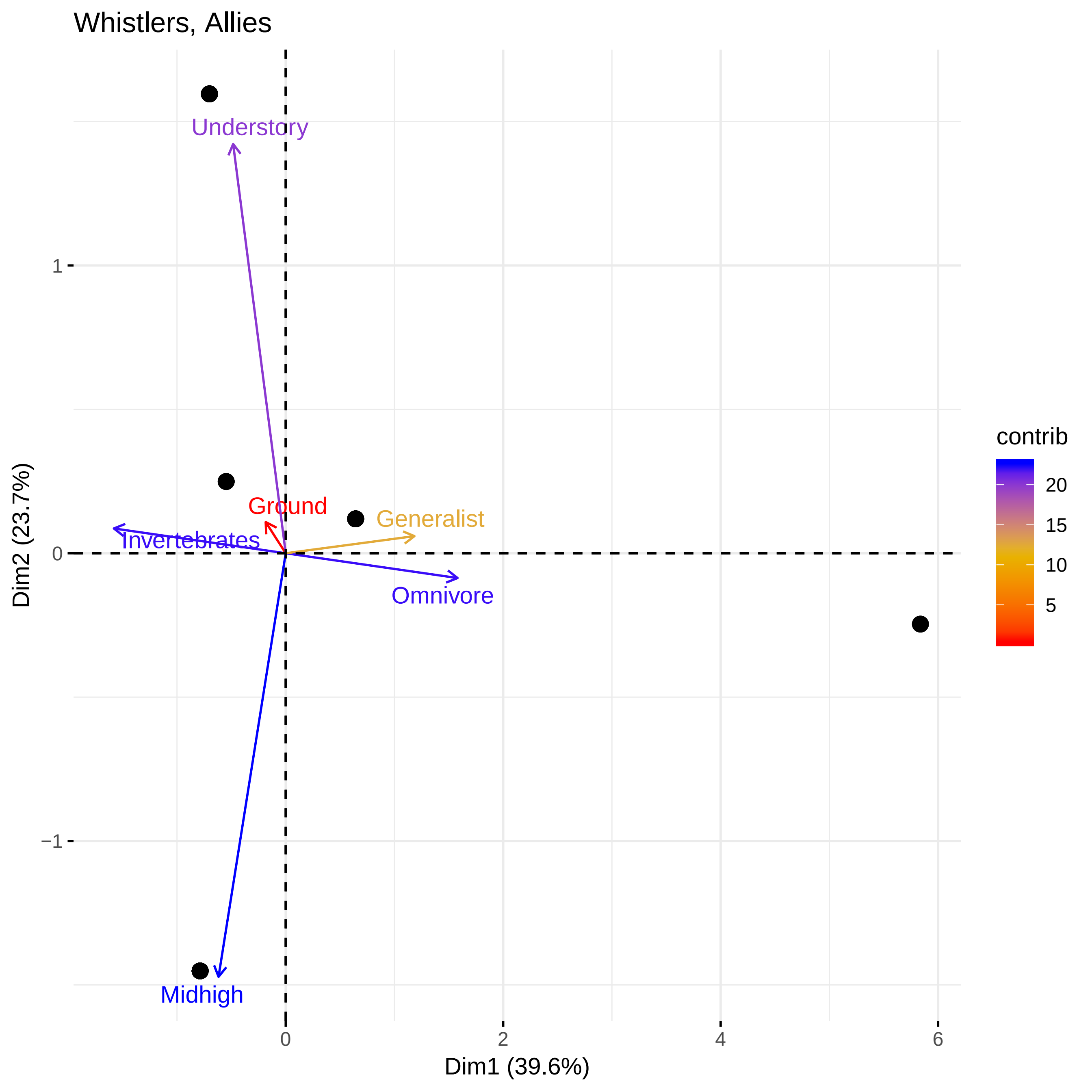

Whistlers, Allies
Understory
Ground
Generalist
Invertebrates
Omnivore
Midhigh
1
0
−1
0
2
4
6
Dim1 (39.6%)
Dim2 (23.7%)
contrib
20
15
10
5

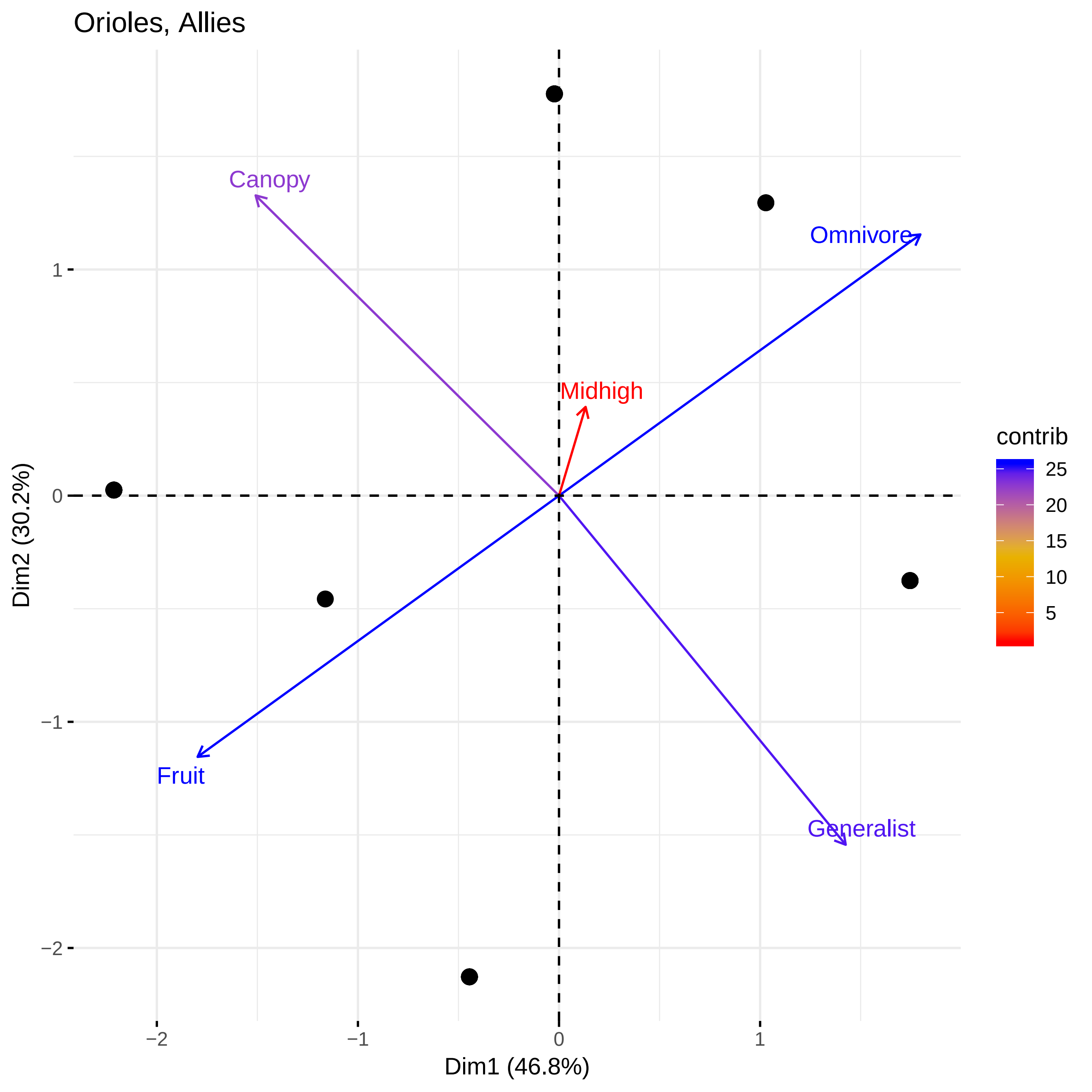

Orioles, Allies
Canopy
Omnivore
Midhigh
Fruit
Generalist
contrib
25
20
15
10
5
Dim1 (46.8%)
Dim2 (30.2%)
−2
−1
0
1

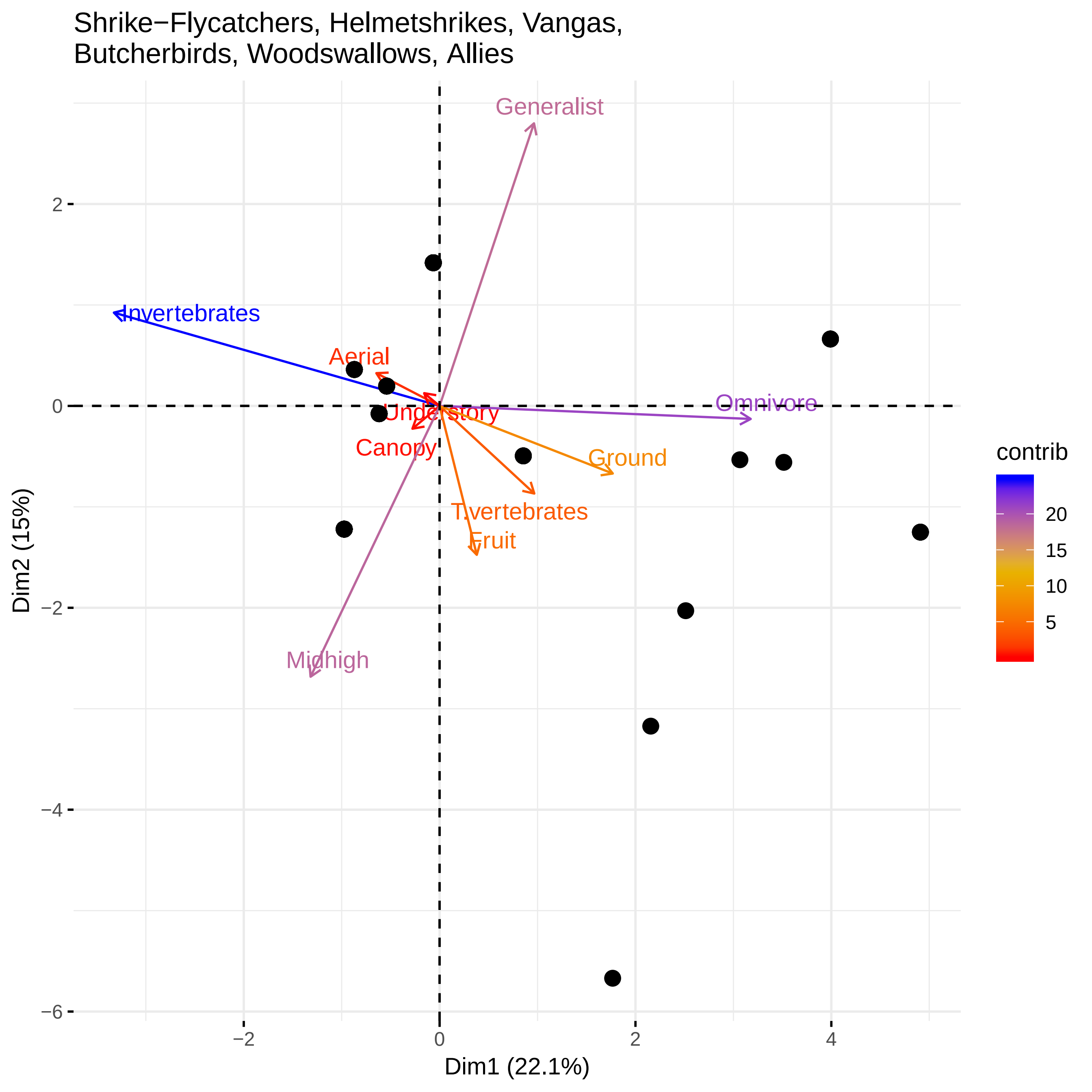

Shrike−Flycatchers, Helmetshrikes, Vangas,
Butcherbirds, Woodswallows, Allies
Generalist
Invertebrates
Aerial
Understory
Canopy
Omnivore
Ground
T.vertebrates
Fruit
Midhigh
contrib
20
15
10
5
Dim2 (15%)
Dim1 (22.1%)
2
0
−2
−4
−6
−2
0
2
4

Waders, Allies

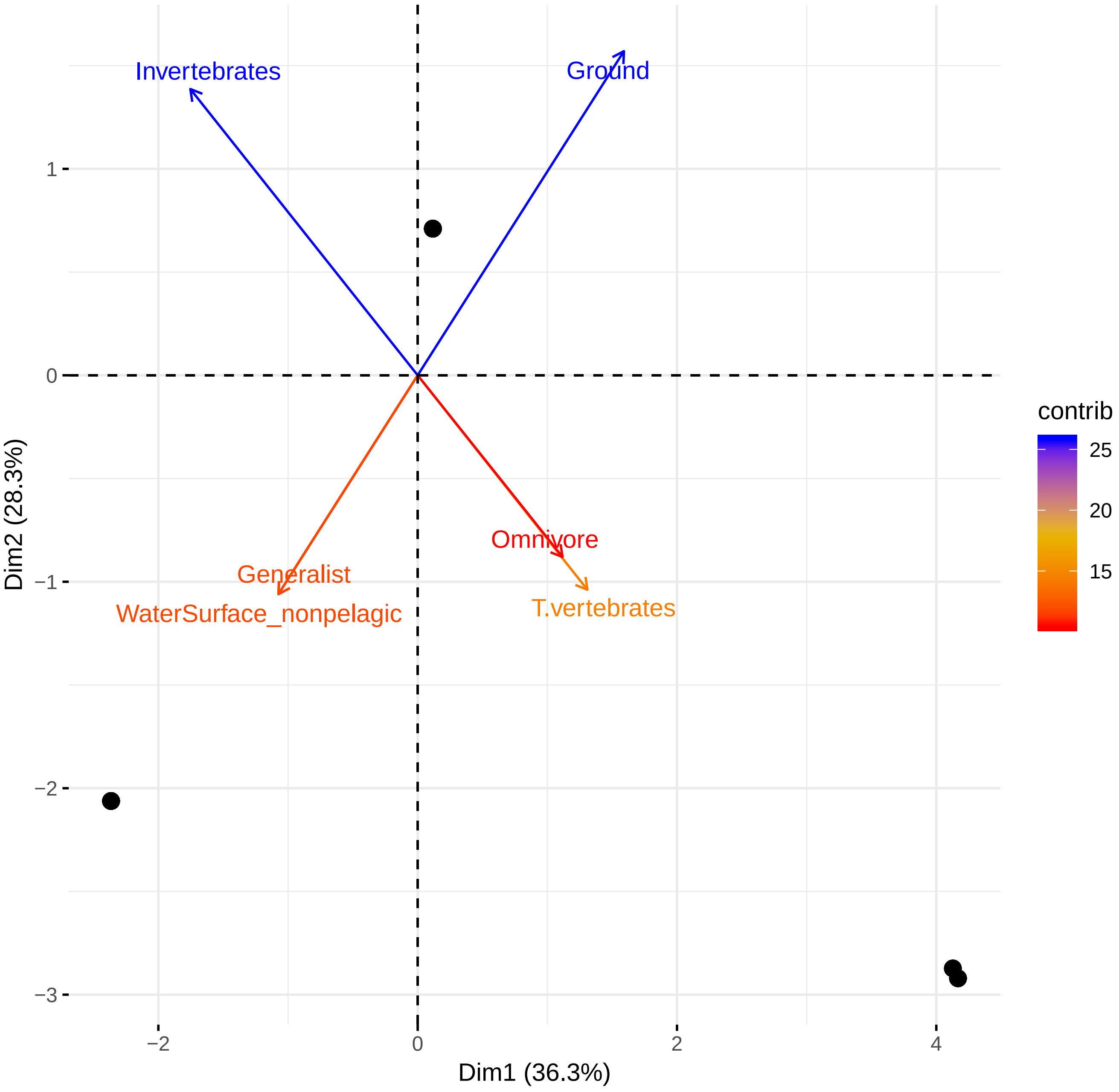

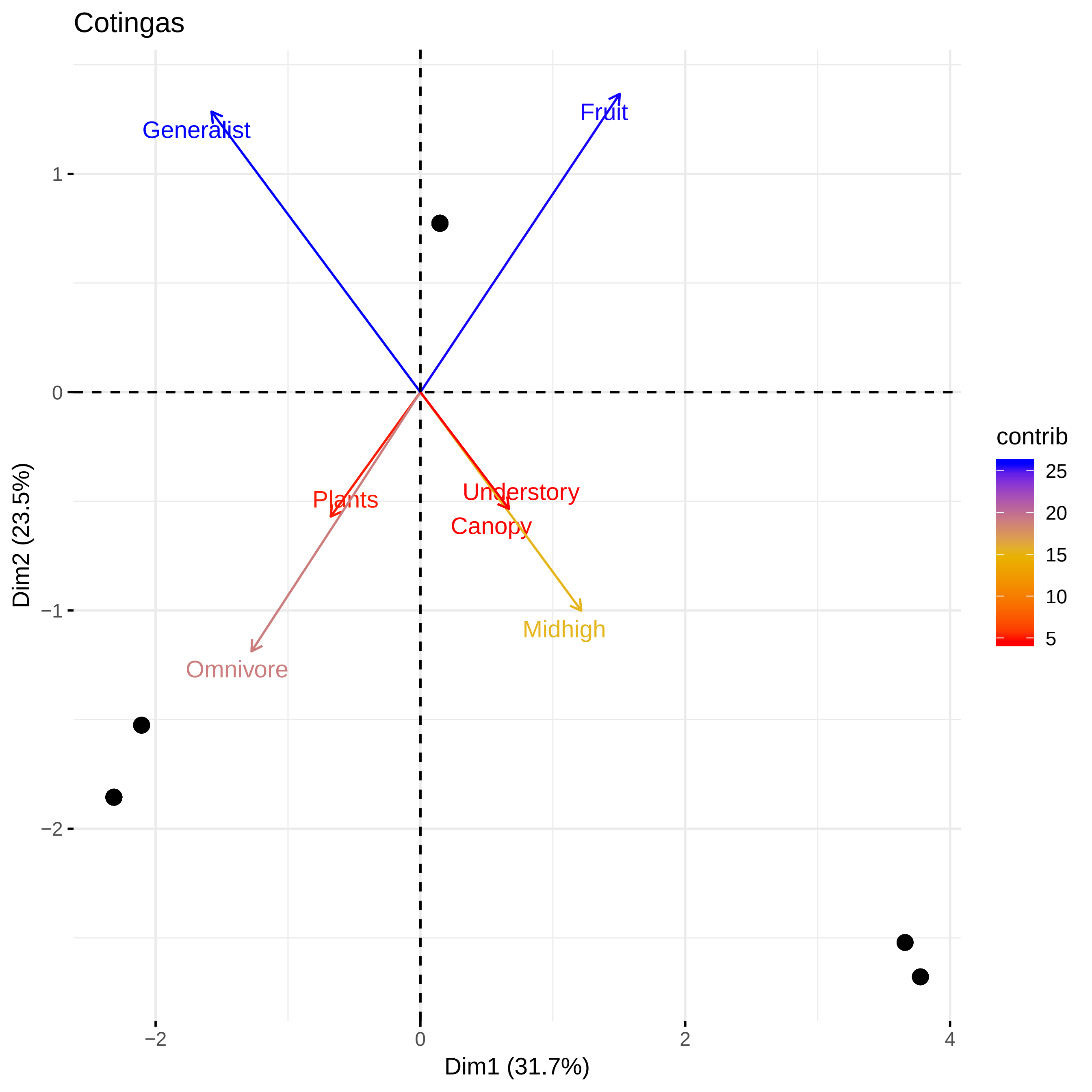
Cotingas
Generalist
Fruit
Plants
Understory
Canopy
Midhigh
Omnivore
1
0
−1
−2
−2
0
2
4
Dim1 (31.7%)
Dim2 (23.5%)
contrib
25
20
15
10
5

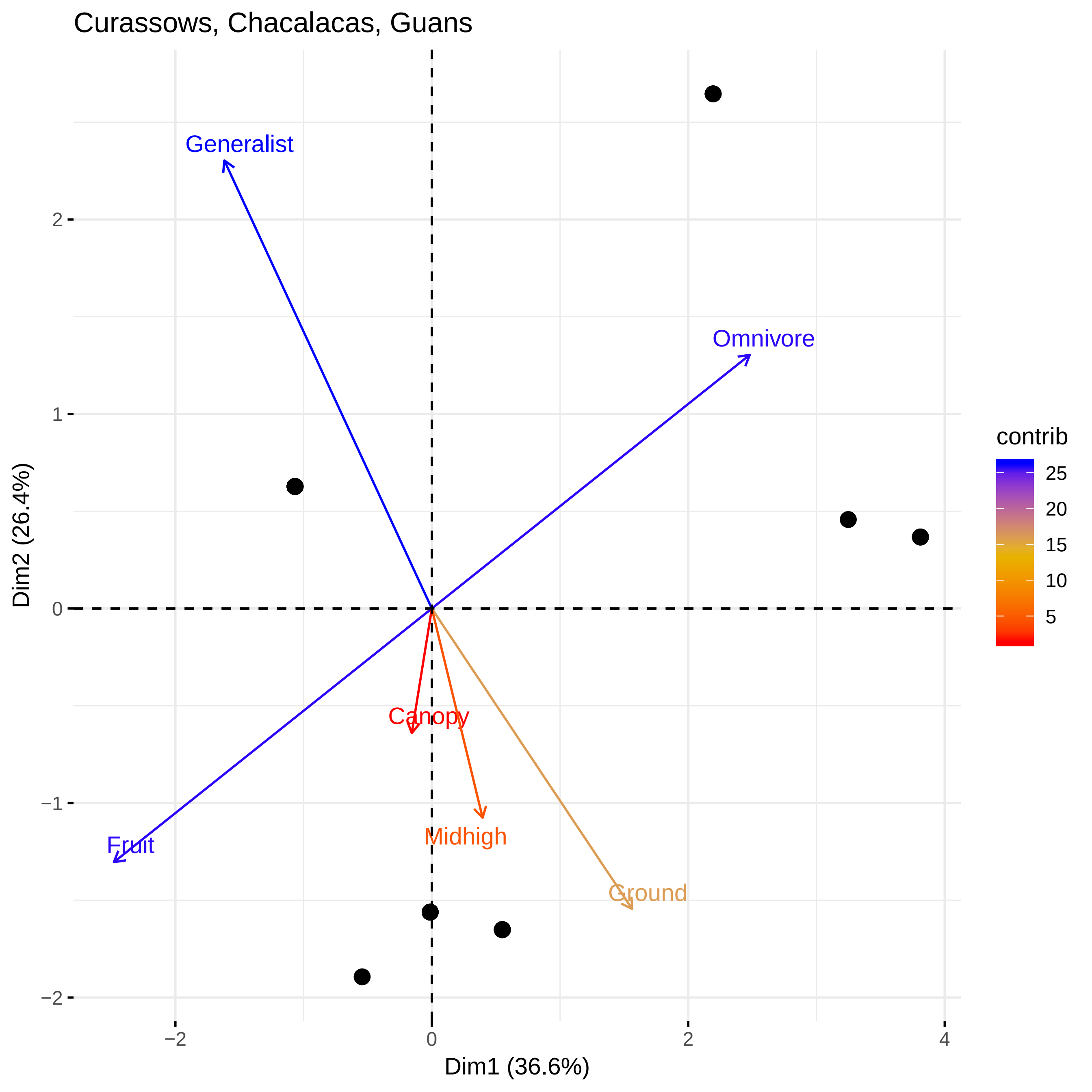
Curassows, Chacalacas, Guans
Generalist
Omnivore
Fruit
Canopy
Midhigh
Ground
contrib
25
20
15
10
5
Dim2 (26.4%)
Dim1 (36.6%)
2
1
0
−1
−2
−2
0
2
4

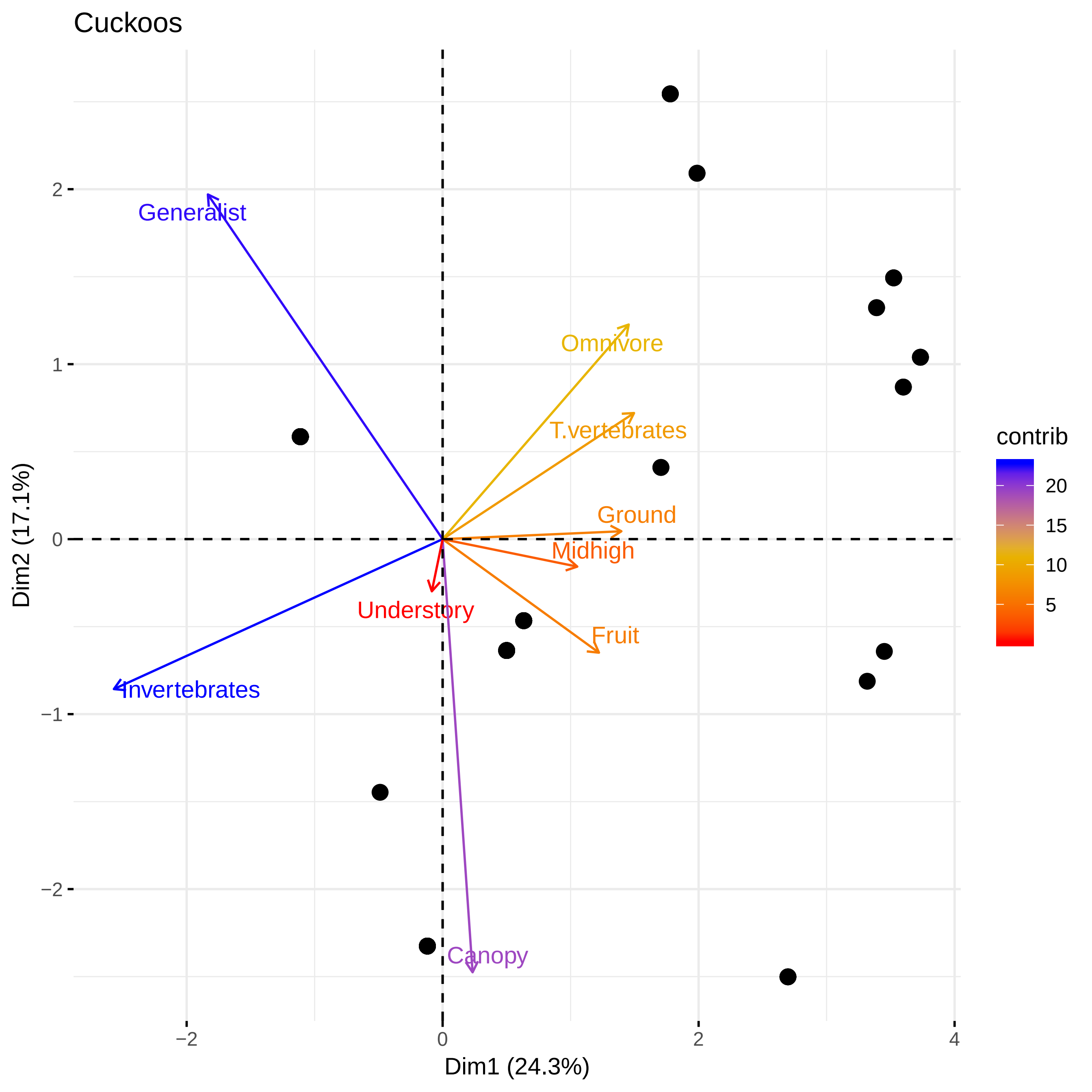
Cuckoos
Generalist
Omnivore
T.vertebrates
Ground
Midhigh
Understory
Fruit
Invertebrates
Canopy
contrib
20
15
10
5
2
1
0
−1
−2
−2
0
2
4
Dim1 (24.3%)
Dim2 (17.1%)

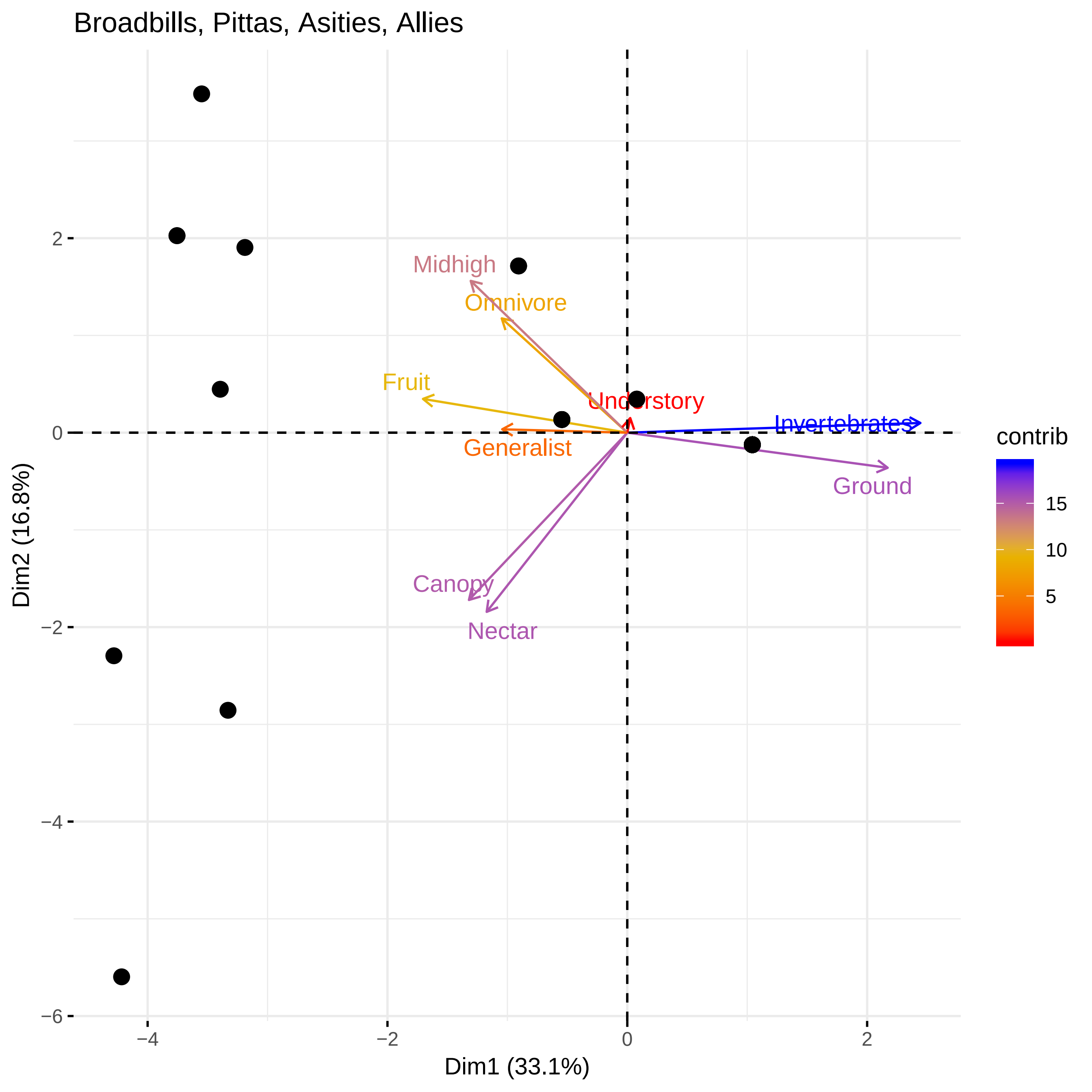

Broadbills, Pittas, Asities, Allies
Midhigh
Omnivore
Fruit
Understory
Invertebrates
Generalist
Ground
Canopy
Nectar
contrib
15
10
5
2
0
−2
−4
−6
−4
−2
0
2
Dim1 (33.1%)
Dim2 (16.8%)

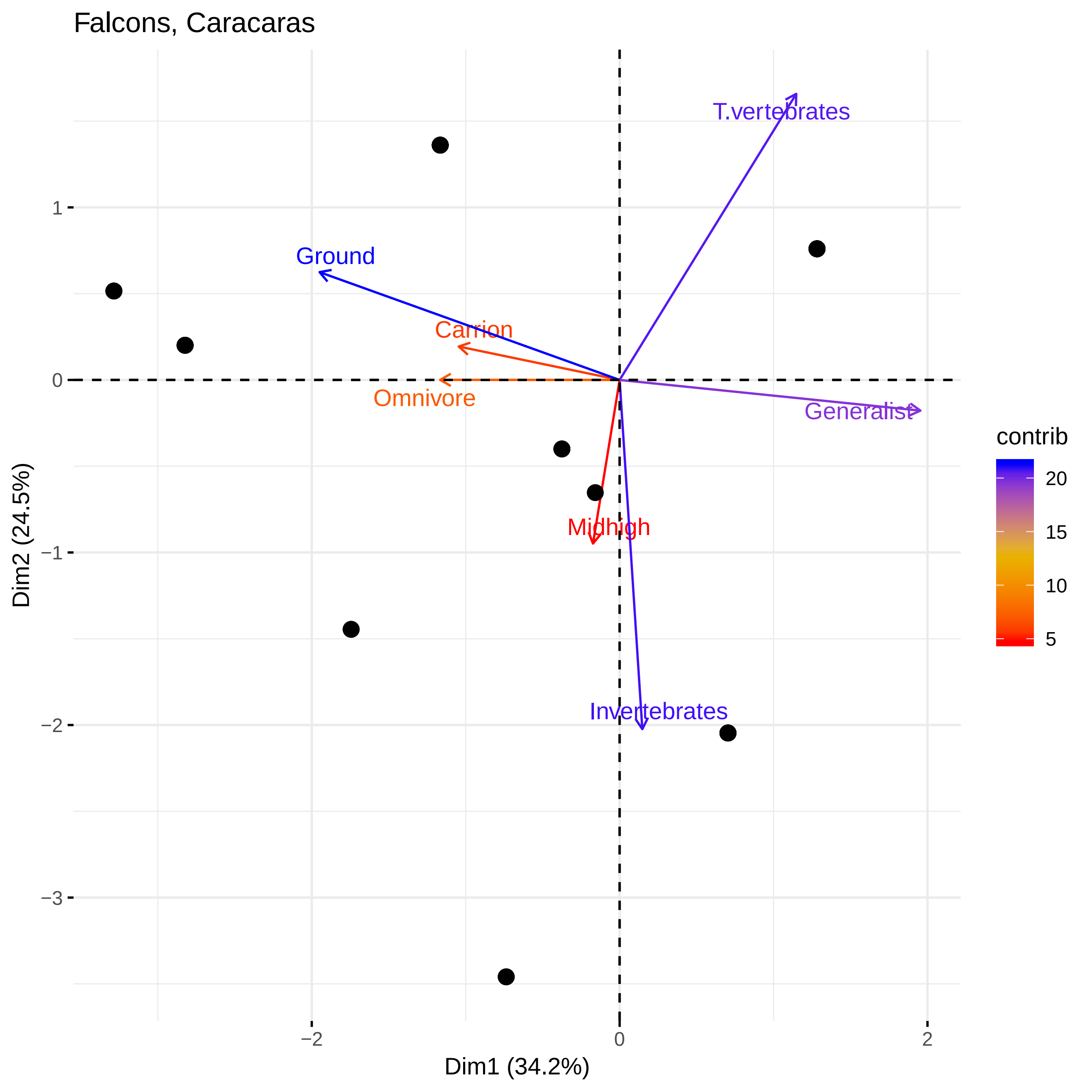
Falcons, Caracaras
T.vertebrates
Ground
Carrion
Omnivore
Generalist
Midhigh
Invertebrates
contrib
20
15
10
5
1
0
−1
−2
−3
−2
0
2
Dim1 (34.2%)
Dim2 (24.5%)

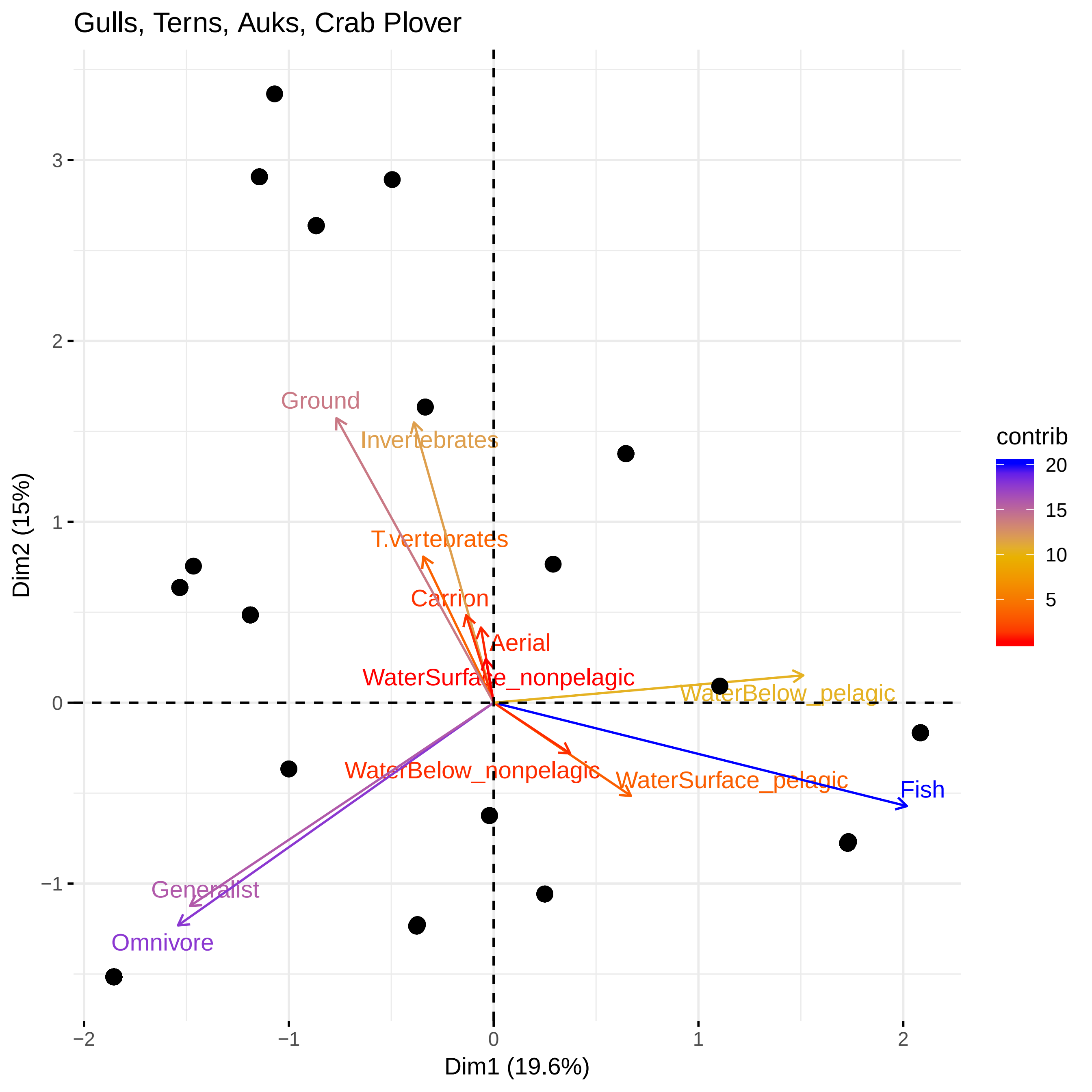

Gulls, Terns, Auks, Crab Plover
Ground
Invertebrates
T.vertebrates
Carrion
Aerial
WaterSurface_nonpelagic
WaterBelow_pelagic
WaterBelow_nonpelagic
WaterSurface_pelagic
Fish
Generalist
Omnivore
contrib
20
15
10
5
3
2
1
0
−1
−2
Dim1 (19.6%)
Dim2 (15%)

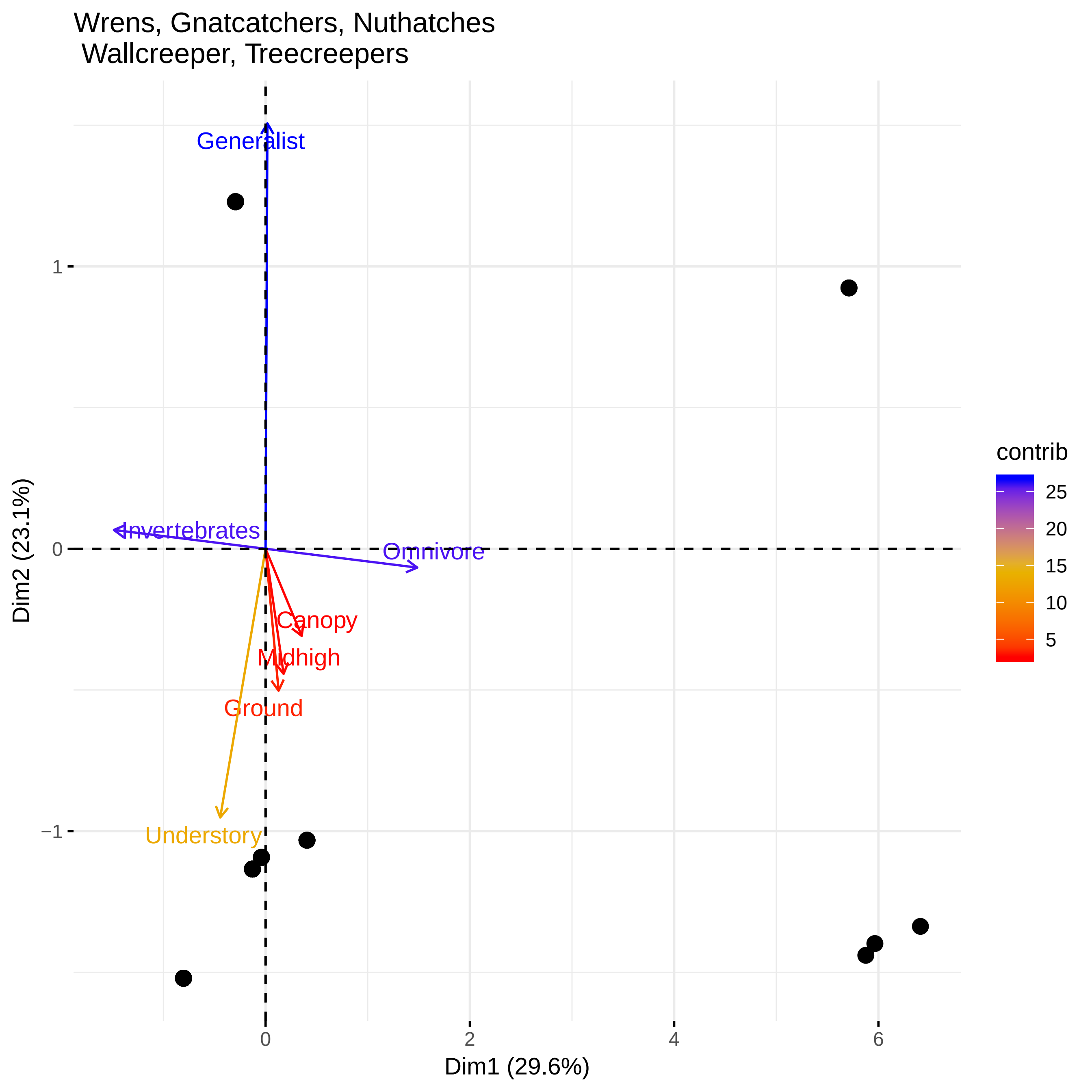
Wrens, Gnatcatchers, Nuthatches
Wallcreeper, Treecreepers
Generalist
Invertebrates
Omnivore
Canopy
Midhigh
Ground
Understory
Dim1 (29.6%)
Dim2 (23.1%)
0
2
4
6
1
0
−1
contrib
25
20
15
10
5

Mockingbirds, Thrashers,
Philippine Creepers, Starlings

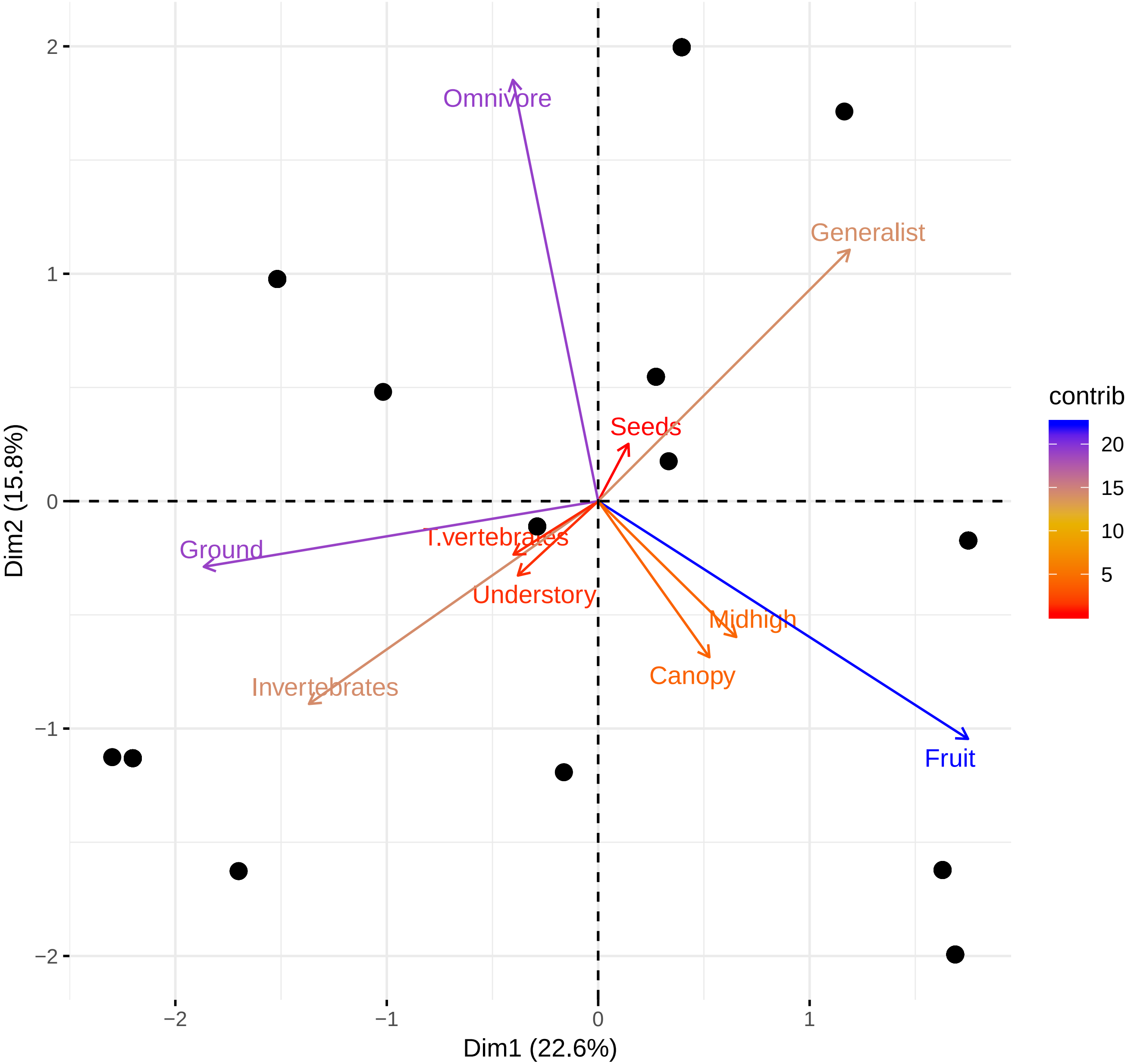

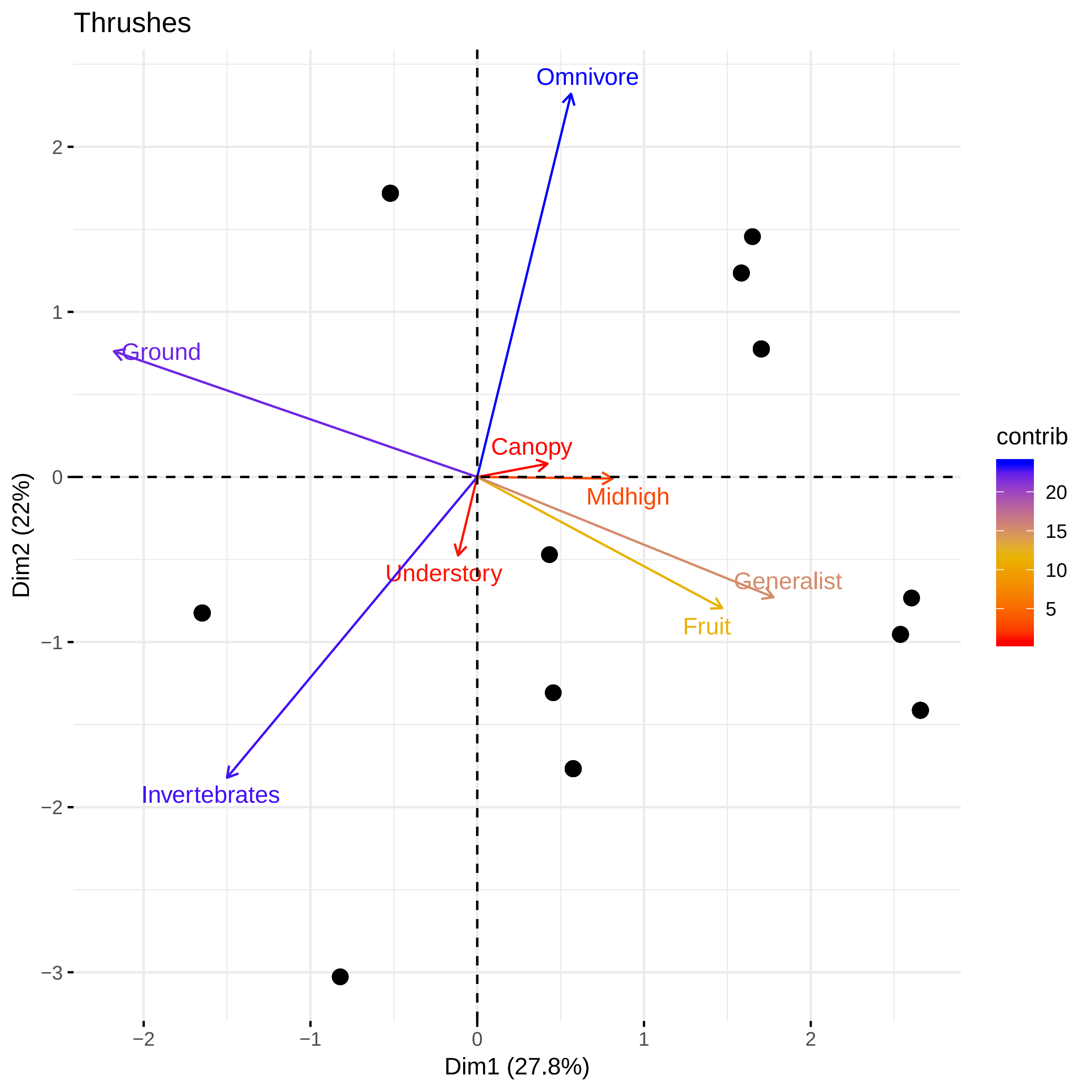

Thrushes
Omnivore
Ground
Canopy
Midhigh
Understory
Generalist
Fruit
Invertebrates
contrib
20
15
10
5
Dim2 (22%)
Dim1 (27.8%)
2
1
0
−1
−2
−3
−2
−1
0
1
2

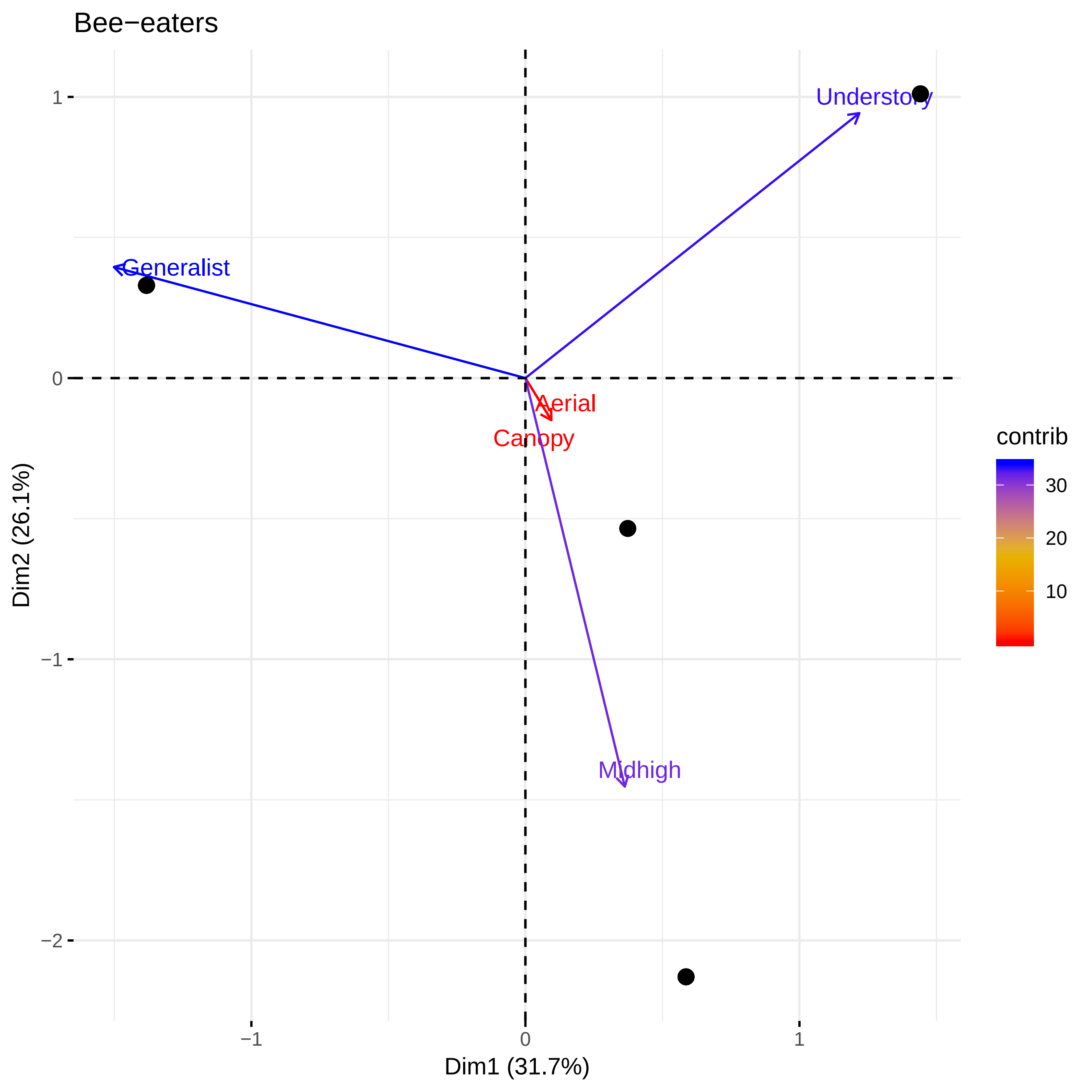

Bee−eaters
Understory
Generalist
Aerial
Canopy
Midhigh
contrib
30
20
10
1
0
−1
−2
−1
0
1
Dim1 (31.7%)
Dim2 (26.1%)

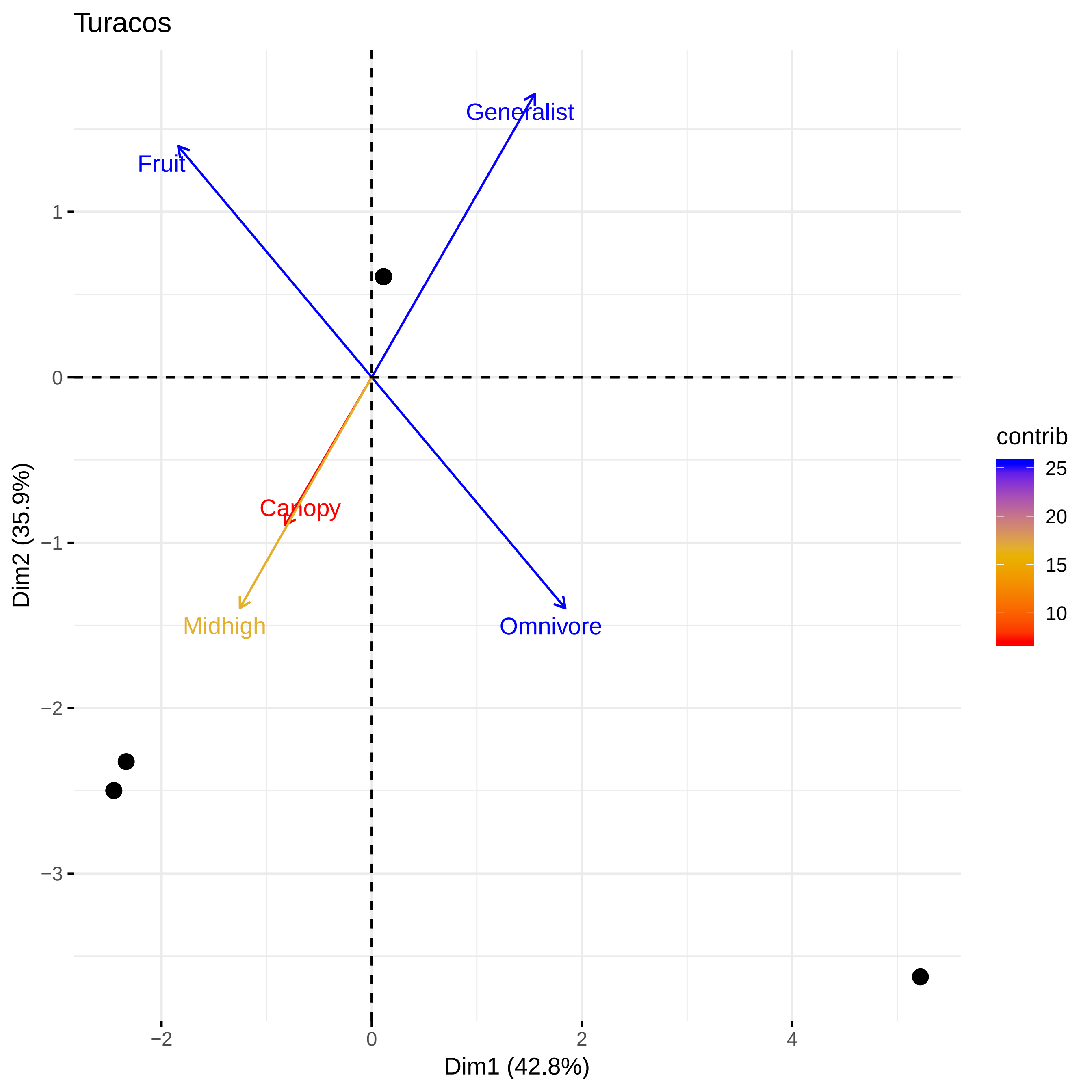
Turacos
Generalist
Fruit
Canopy
Midhigh
Omnivore
contrib
25
20
15
10
1
0
−1
−2
−3
−2
0
2
4
Dim1 (42.8%)
Dim2 (35.9%)

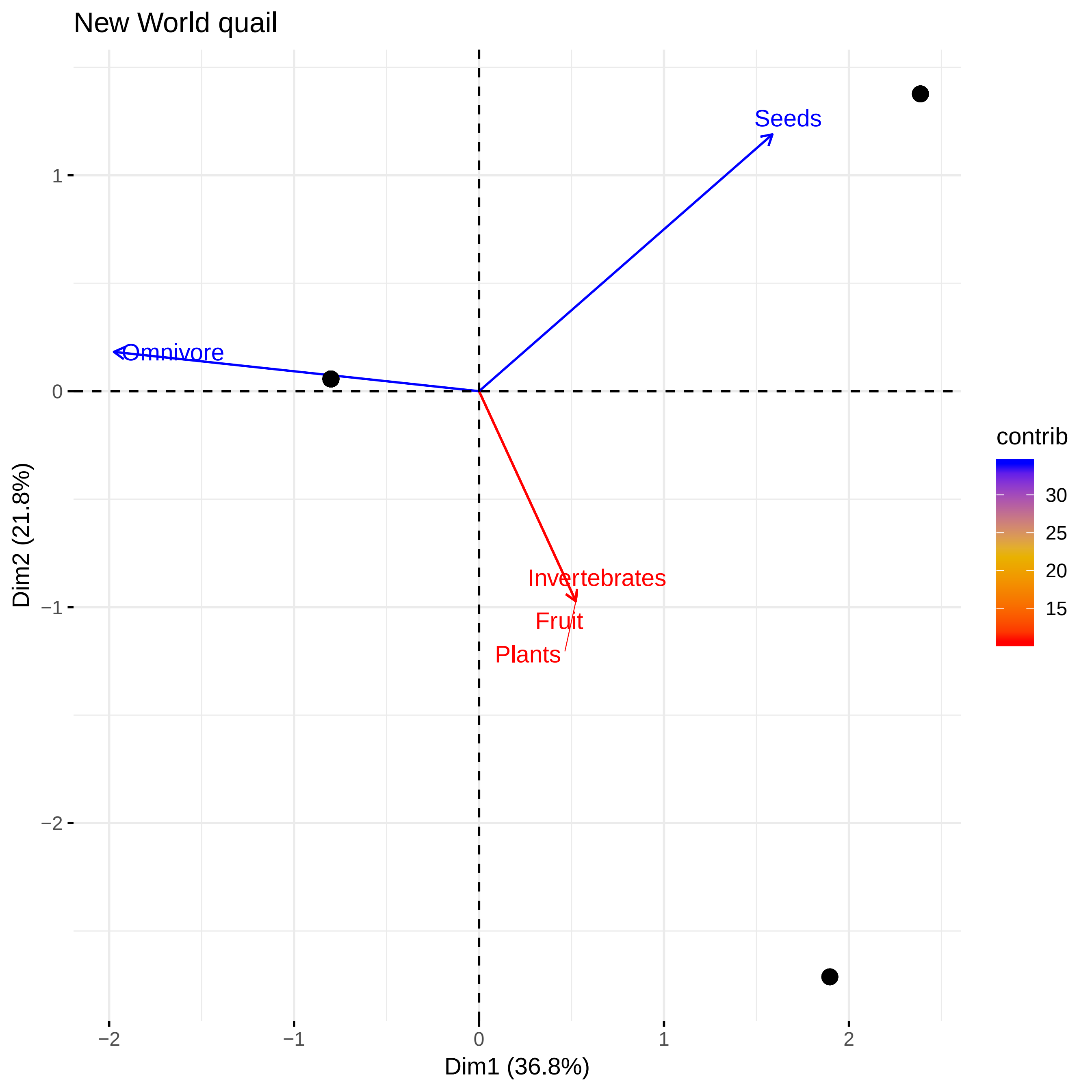

New World quail
Seeds
Omnivore
Invertebrates
Fruit
Plants
1
0
−1
−2
−2
−1
0
1
2
Dim1 (36.8%)
Dim2 (21.8%)
contrib
30
25
20
15

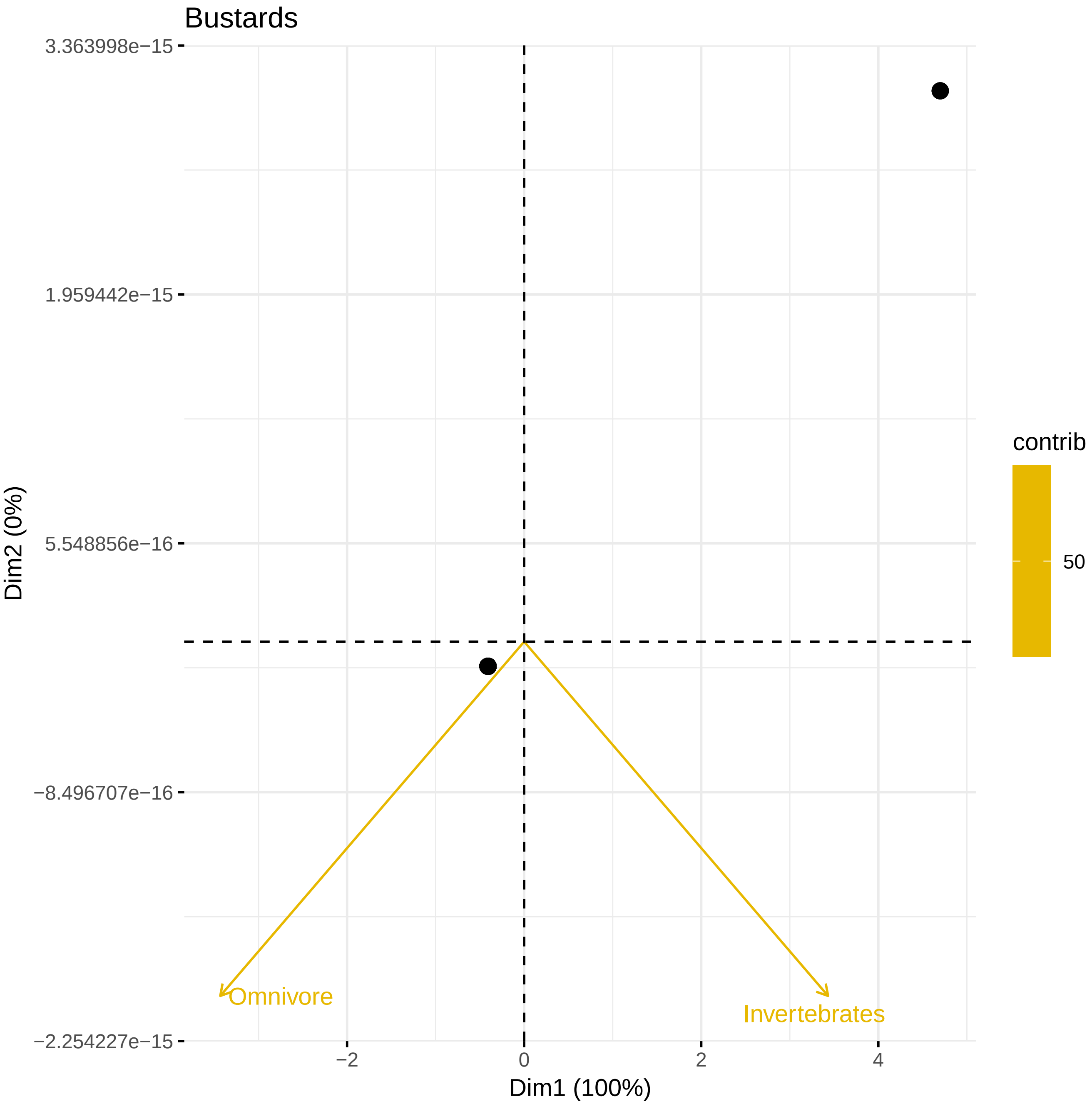
Bustards
3.363998e−15
1.959442e−15
5.548856e−16
−8.496707e−16
−2.254227e−15
Dim2 (0%)
−2
0
2
4
Dim1 (100%)
Omnivore
Invertebrates
contrib
50

Finches, Allies

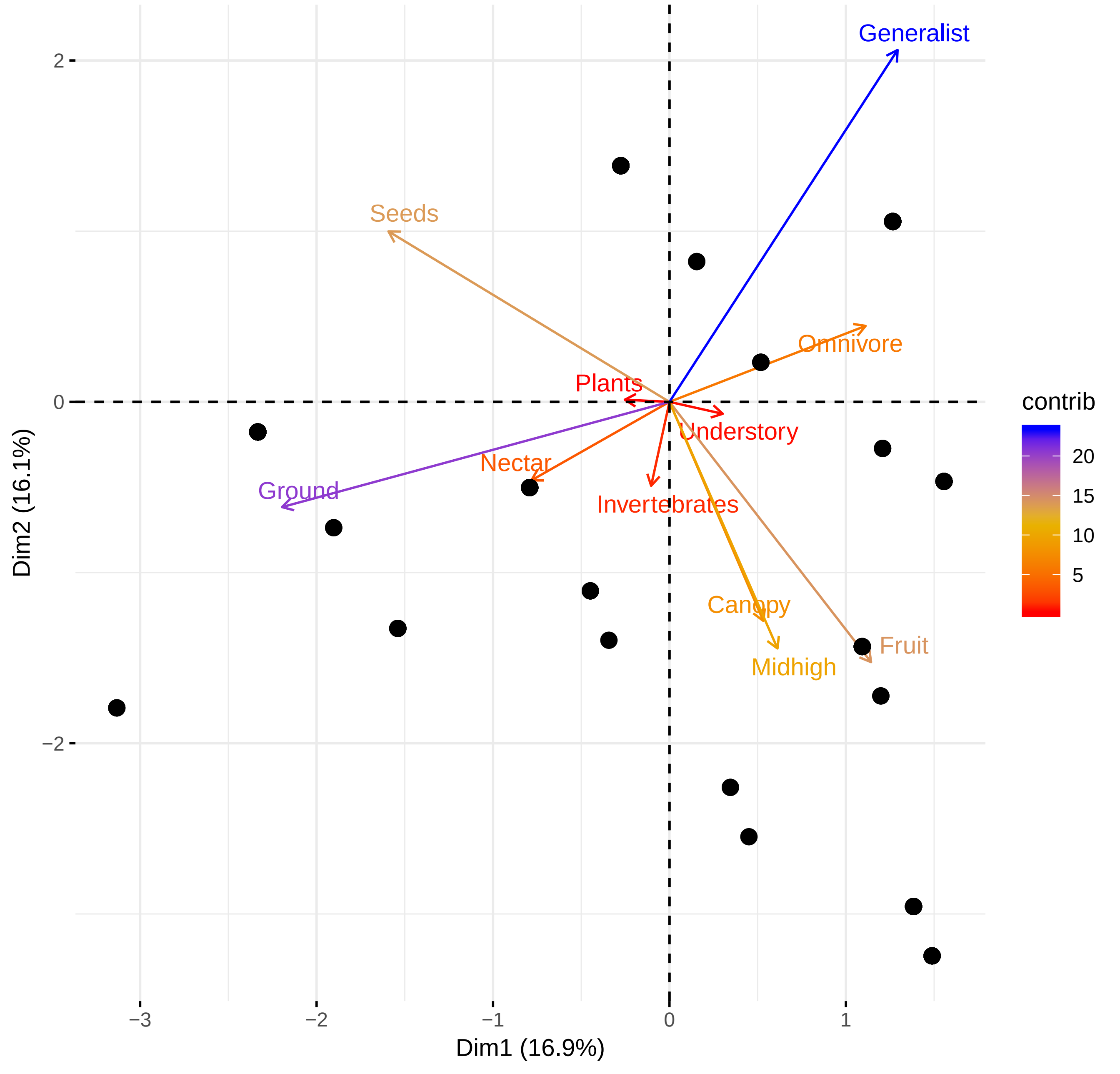

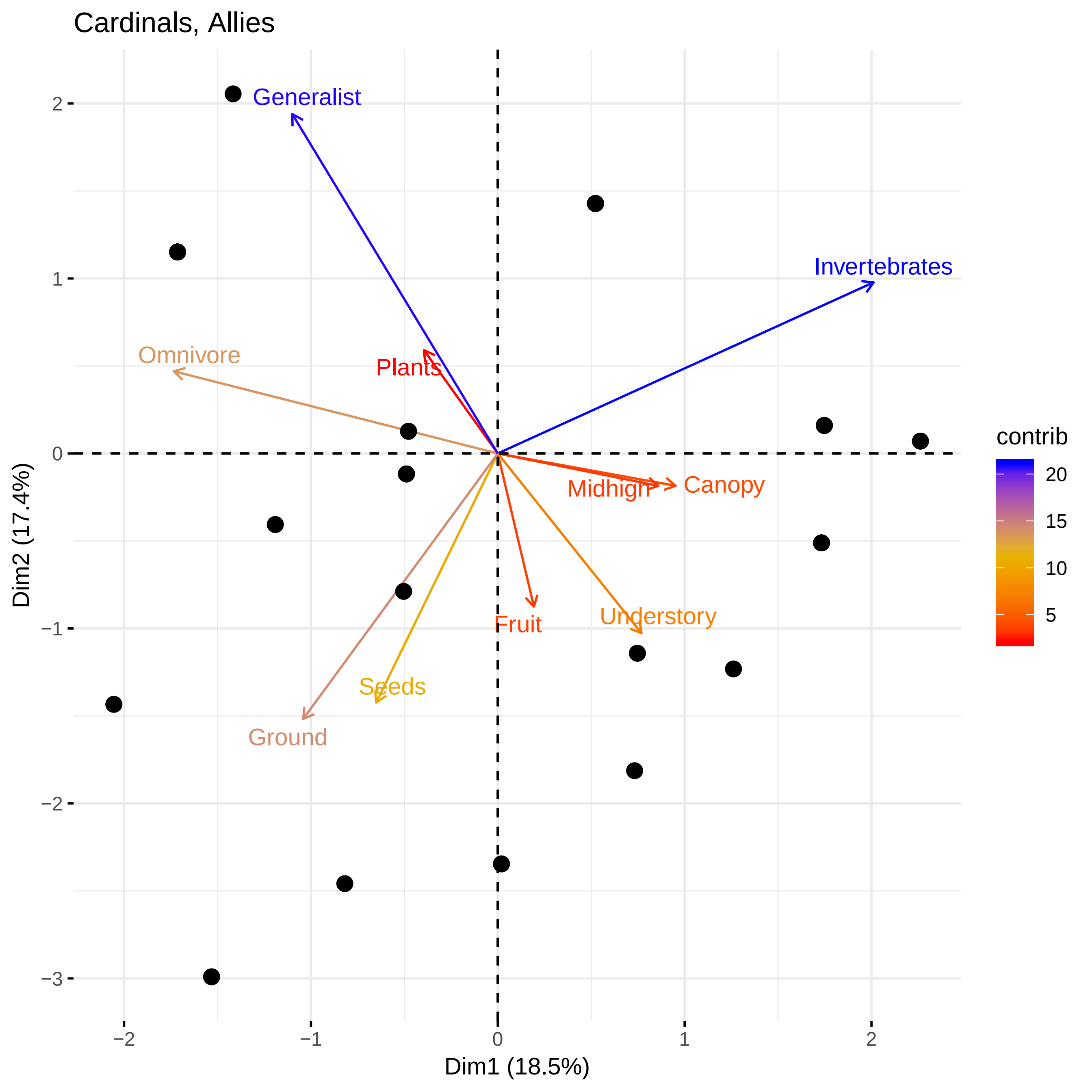
Cardinals, Allies
Generalist
Invertebrates
Omnivore
Plants
Midhigh
Canopy
Fruit
Understory
Seeds
Ground
contrib
20
15
10
5
Dim2 (17.4%)
Dim1 (18.5%)
2
1
0
−1
−2
−3
−2
−1
0
1
2

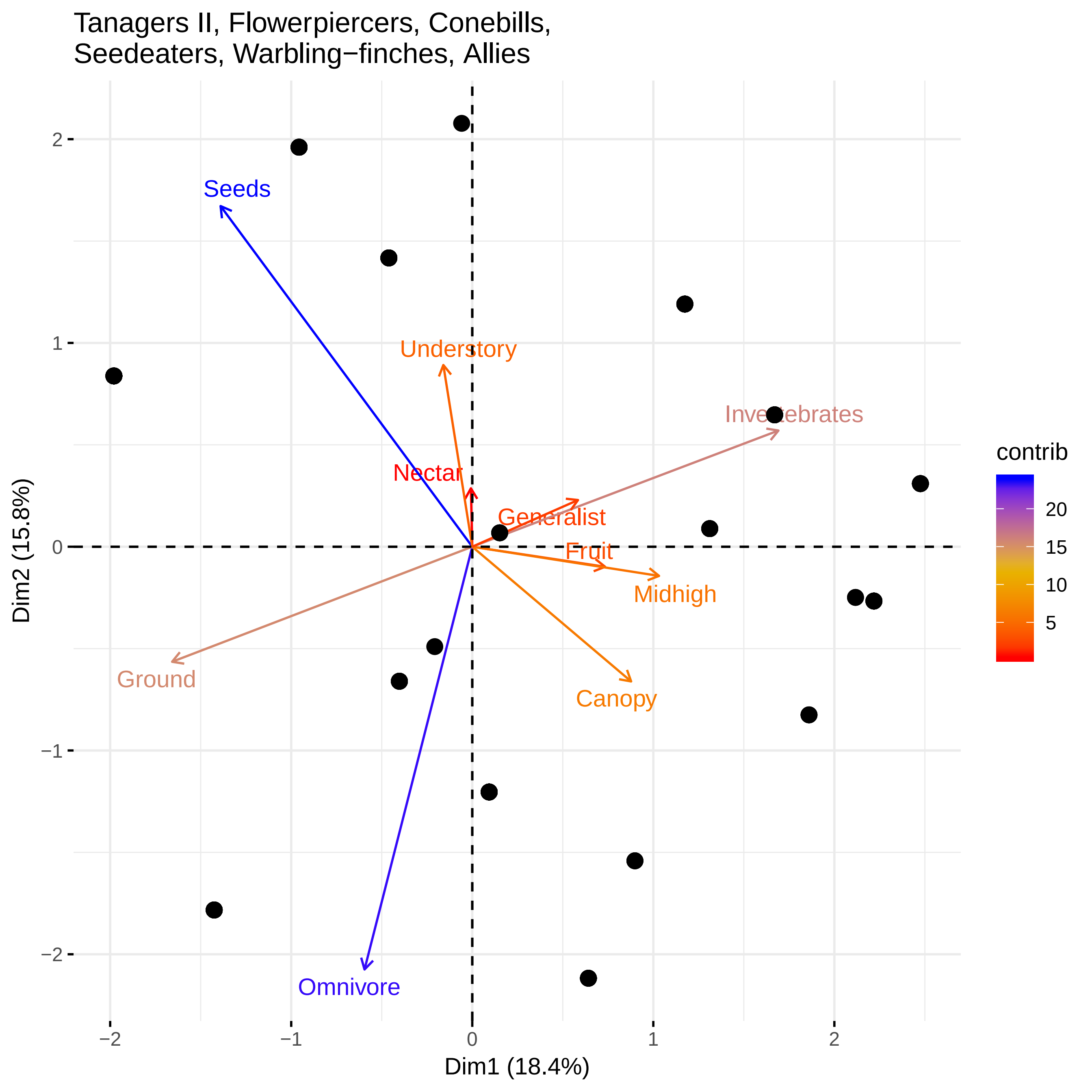
Tanagers II, Flowerpiercers, Conebills,
Seedeaters, Warbling−finches, Allies
Seeds
Understory
Invertebrates
Nectar
Generalist
Fruit
Midhigh
Ground
Canopy
Omnivore
contrib
20
15
10
5
2
1
0
−1
−2
Dim2 (15.8%)
Dim1 (18.4%)

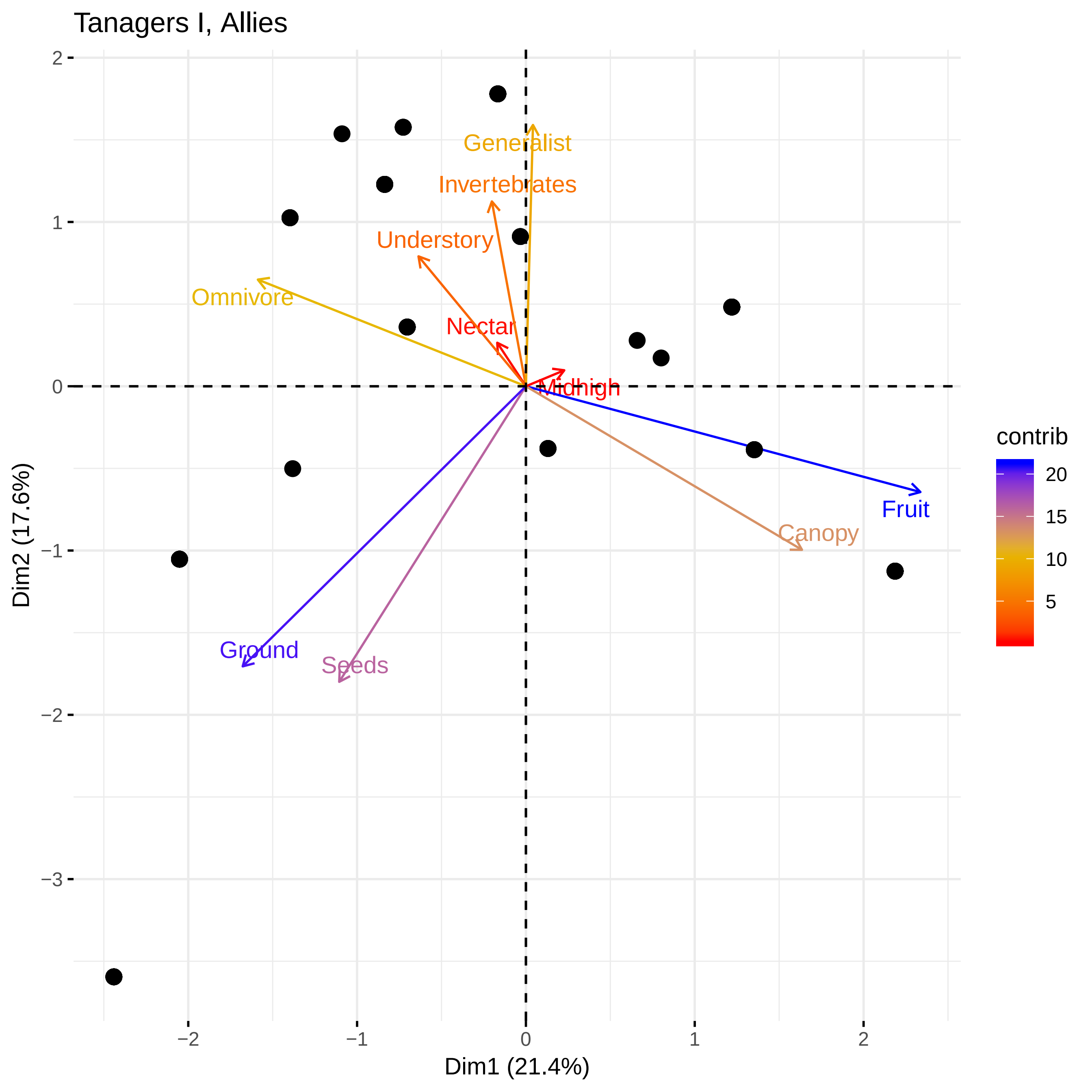

Tanagers I, Allies
Generalist
Invertebrates
Understory
Omnivore
Nectar
Midhigh
Fruit
Canopy
Ground
Seeds
contrib
20
15
10
5
Dim2 (17.6%)
Dim1 (21.4%)
2
1
0
−1
−2
−3

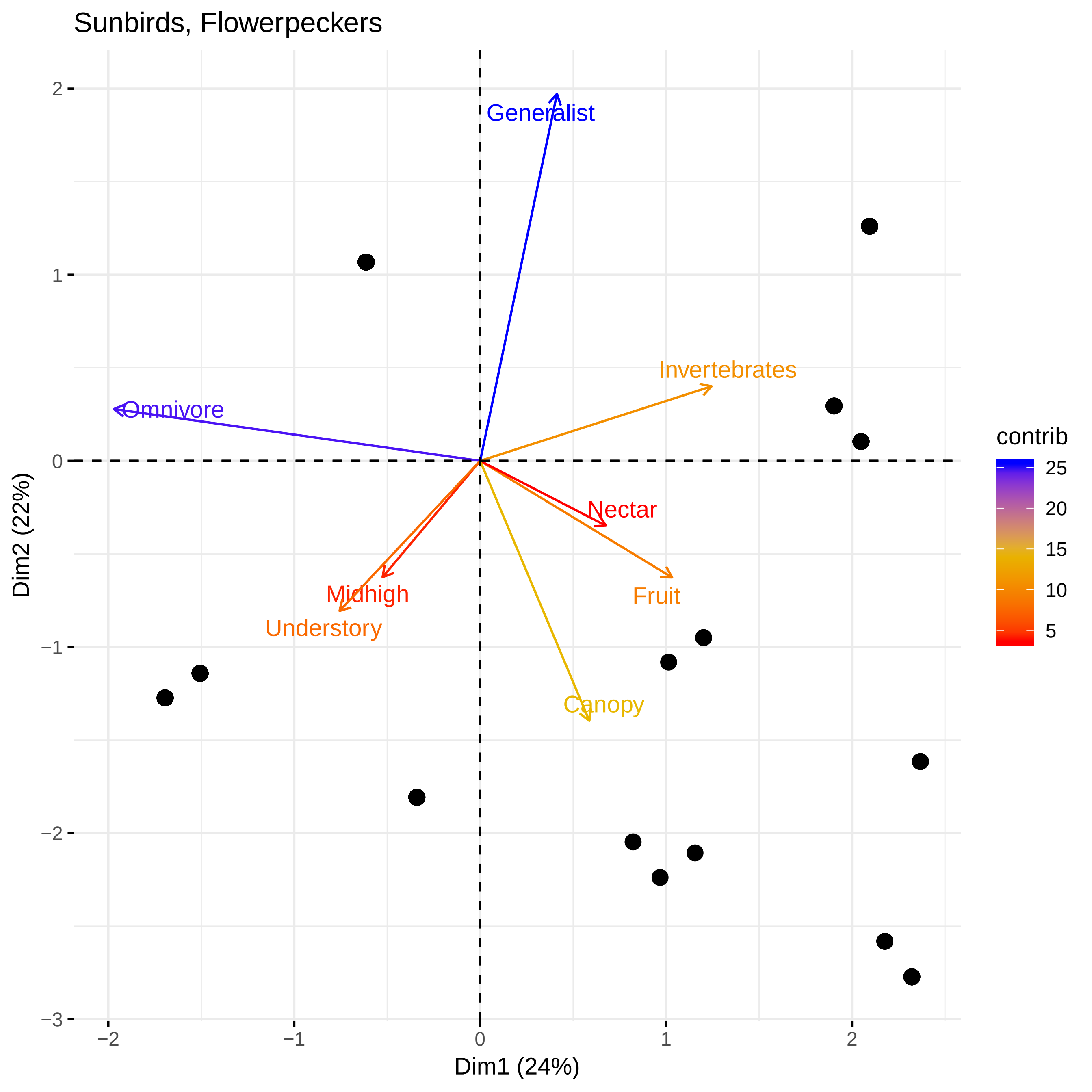

Sunbirds, Flowerpeckers
Generalist
Invertebrates
Omnivore
Nectar
Fruit
Midhigh
Understory
Canopy
contrib
25
20
15
10
5
2
1
0
−1
−2
−3
Dim2 (22%)
−2
−1
0
1
2
Dim1 (24%)

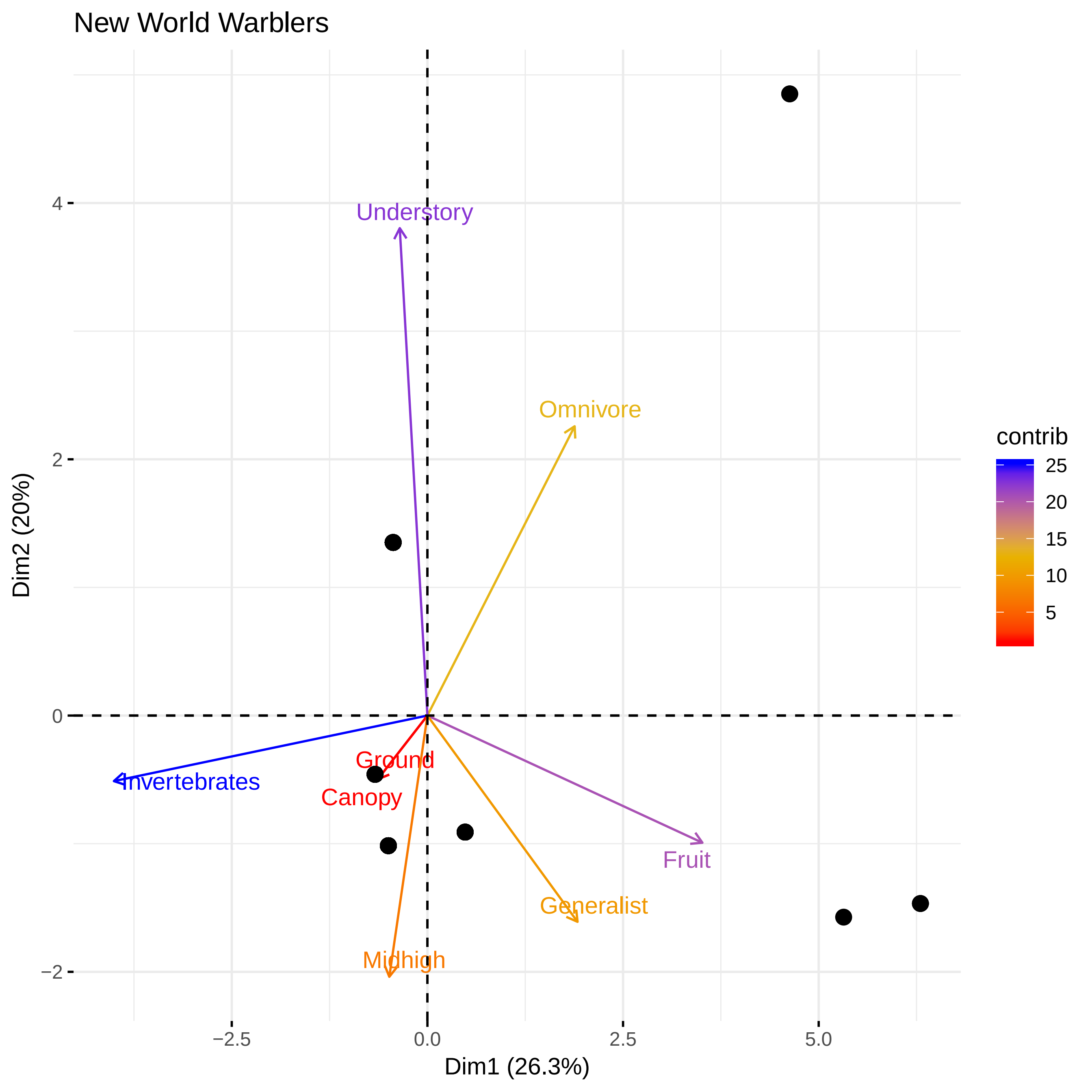

New World Warblers
Understory
Omnivore
Ground
Invertebrates
Canopy
Fruit
Generalist
Midhigh
contrib
25
20
15
10
5
4
2
0
−2
−2.5
0.0
2.5
5.0
Dim1 (26.3%)
Dim2 (20%)

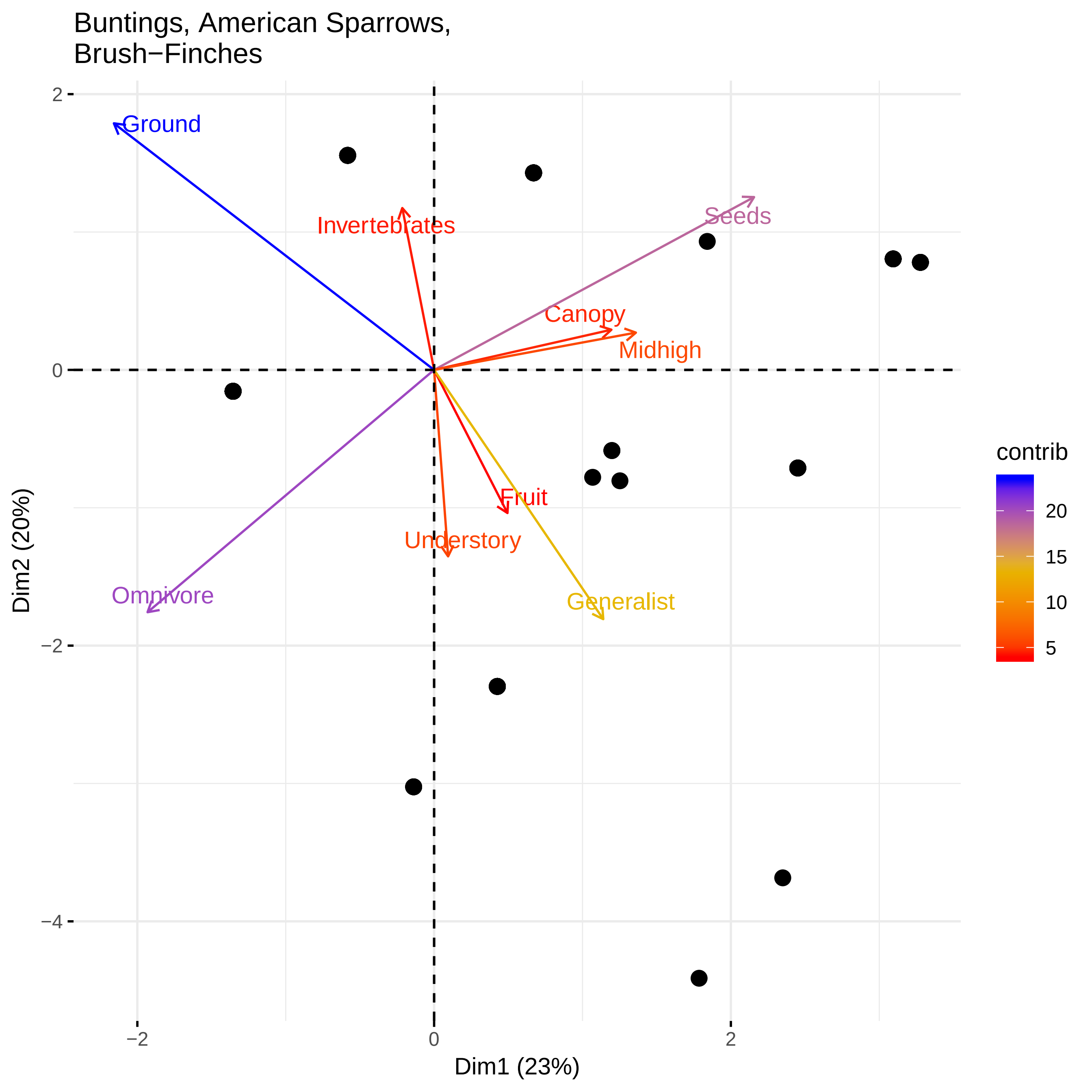

Buntings, American Sparrows,
Brush−Finches
Ground
Invertebrates
Seeds
Canopy
Midhigh
Fruit
Understory
Omnivore
Generalist
2
0
−2
−4
−2
0
2
Dim1 (23%)
Dim2 (20%)
contrib
20
15
10
5

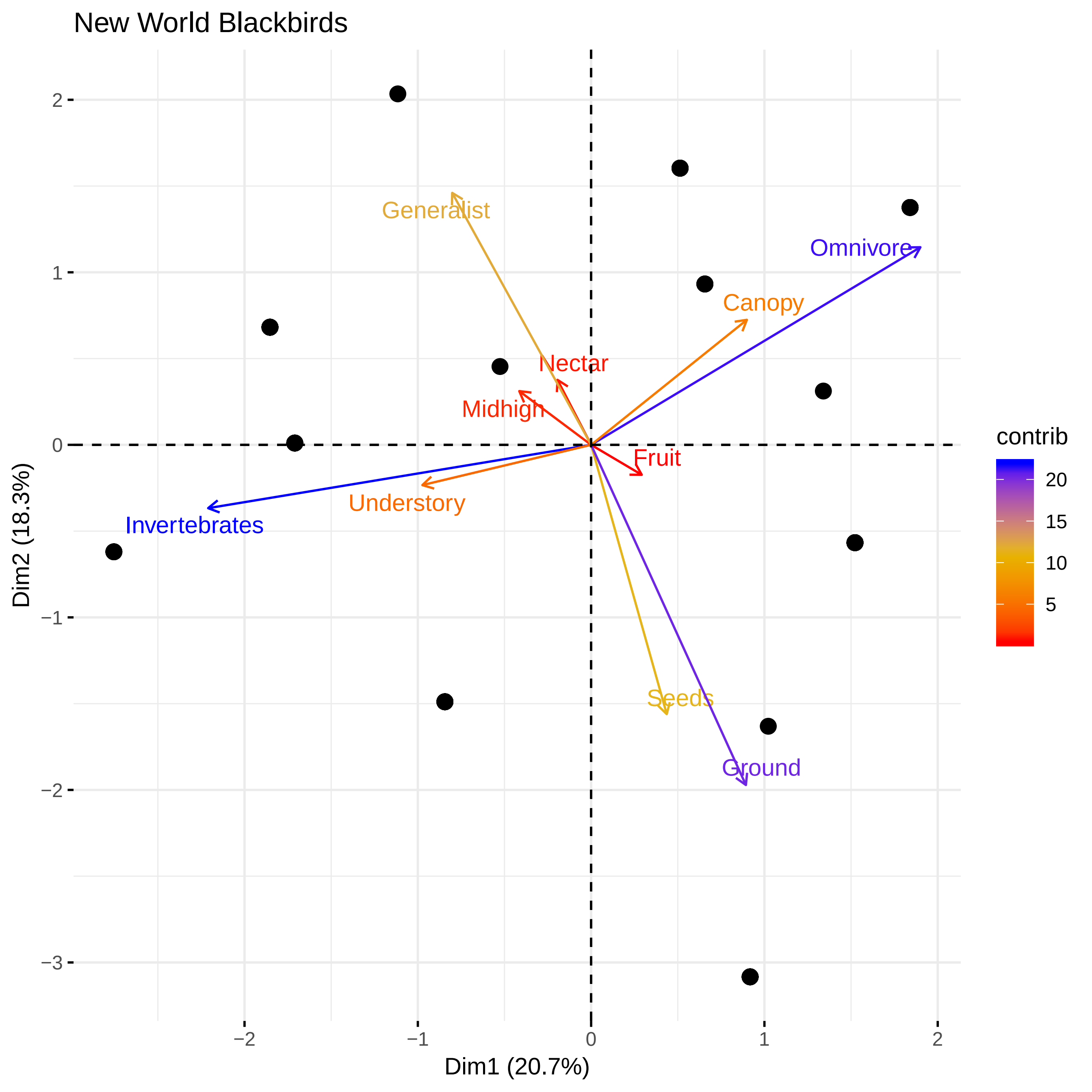

New World Blackbirds
Generalist
Omnivore
Canopy
Nectar
Midhigh
Fruit
Understory
Invertebrates
Seeds
Ground
contrib
20
15
10
5
Dim1 (20.7%)
Dim2 (18.3%)
−2
−1
0
1
2
−3

Waxbills, Allies

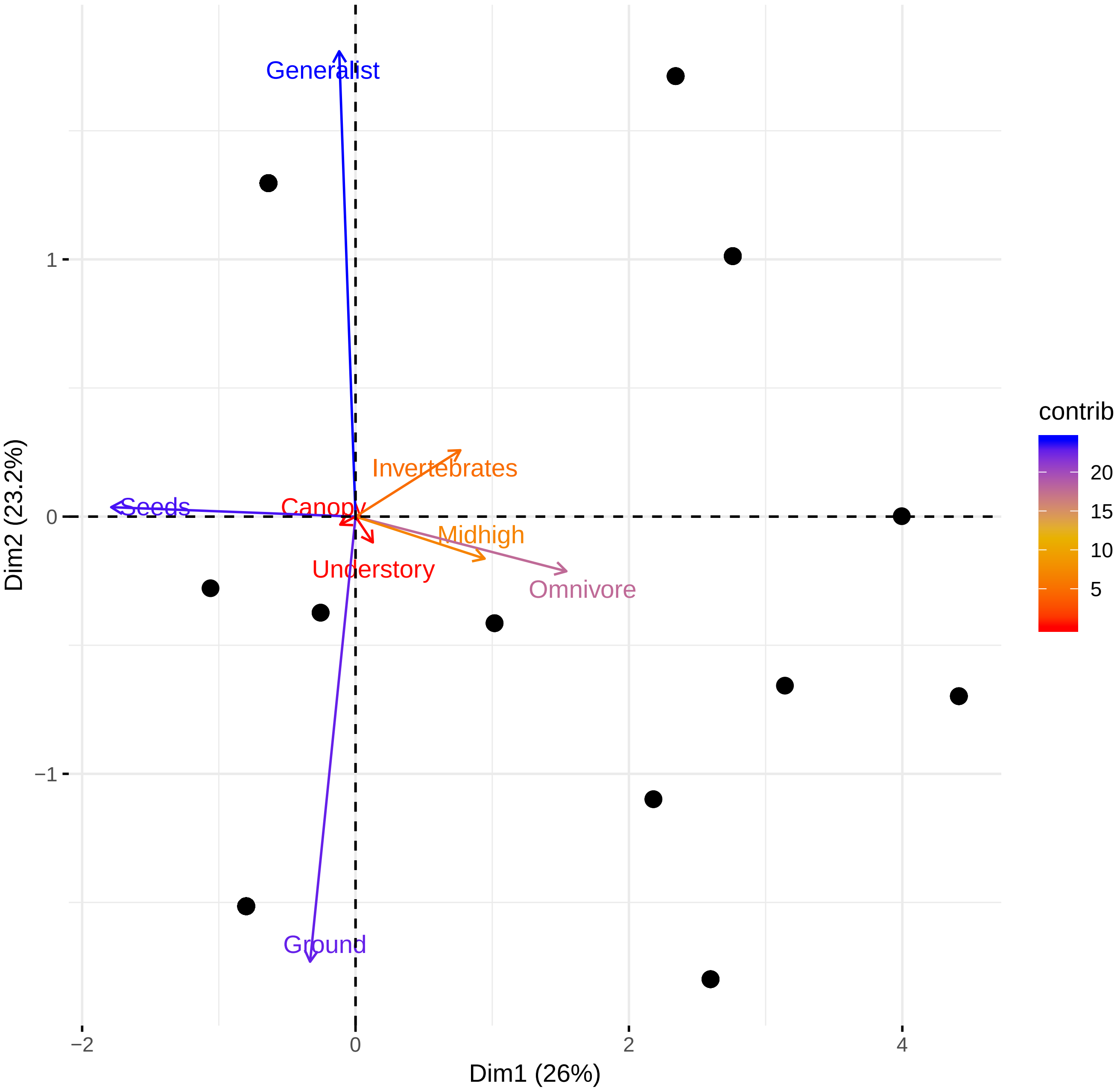

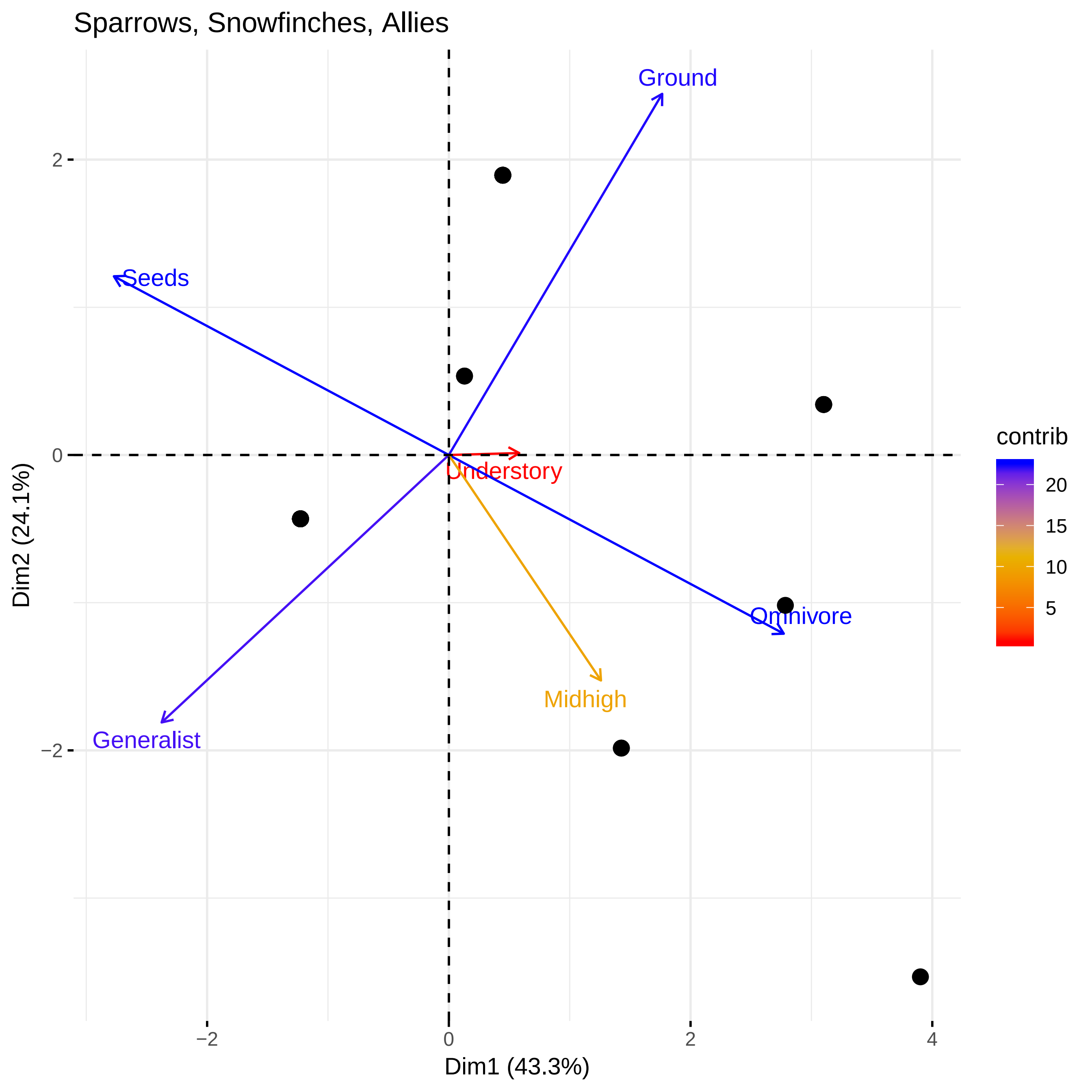
Sparrows, Snowfinches, Allies
Ground
Seeds
Understory
Omnivore
Midhigh
Generalist
contrib
20
15
10
5
2
0
−2
−2
0
2
4
Dim1 (43.3%)
Dim2 (24.1%)

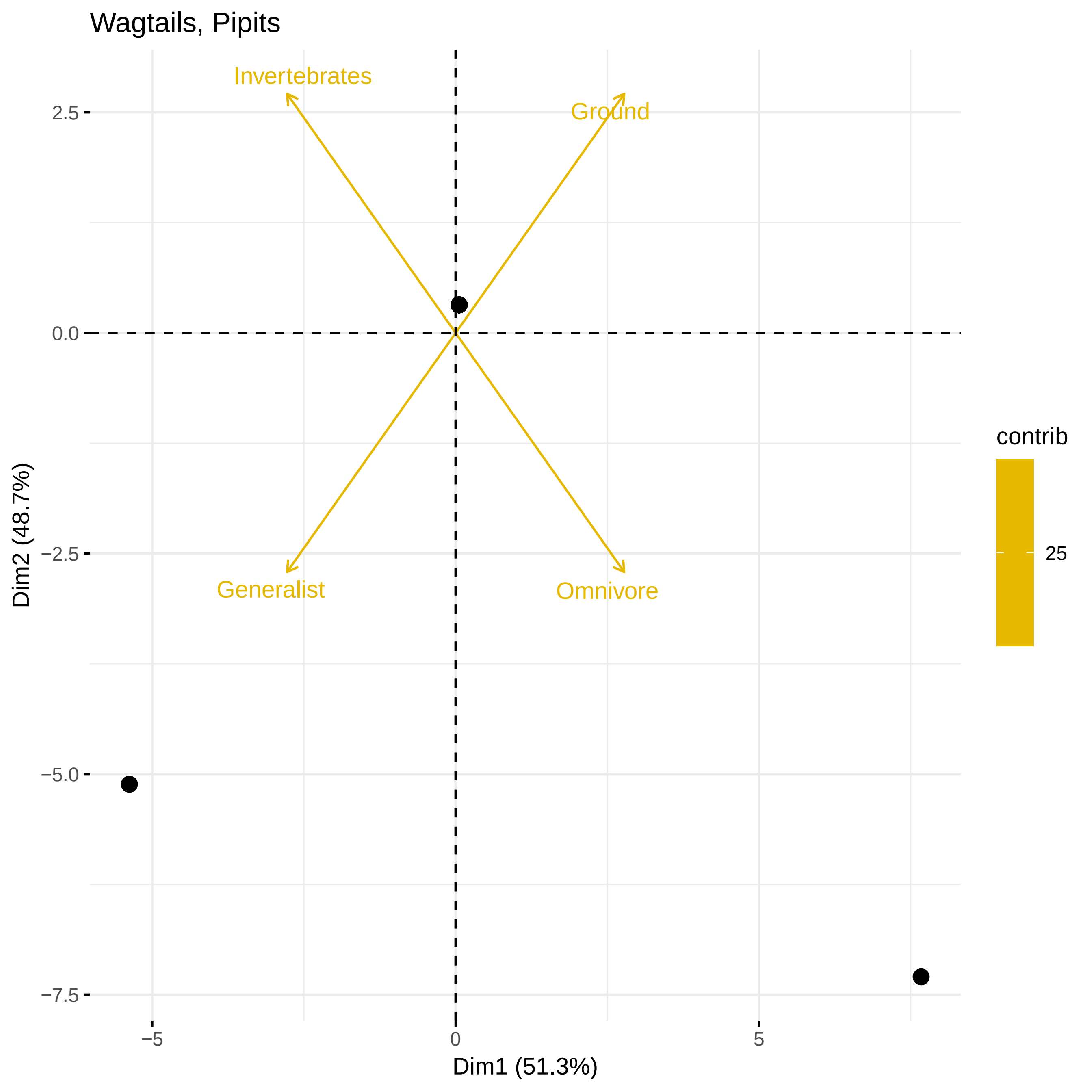
Wagtails, Pipits
Invertebrates
Ground
Generalist
Omnivore
2.5
0.0
−2.5
−5.0
−7.5
−5
0
5
Dim1 (51.3%)
Dim2 (48.7%)
contrib
25

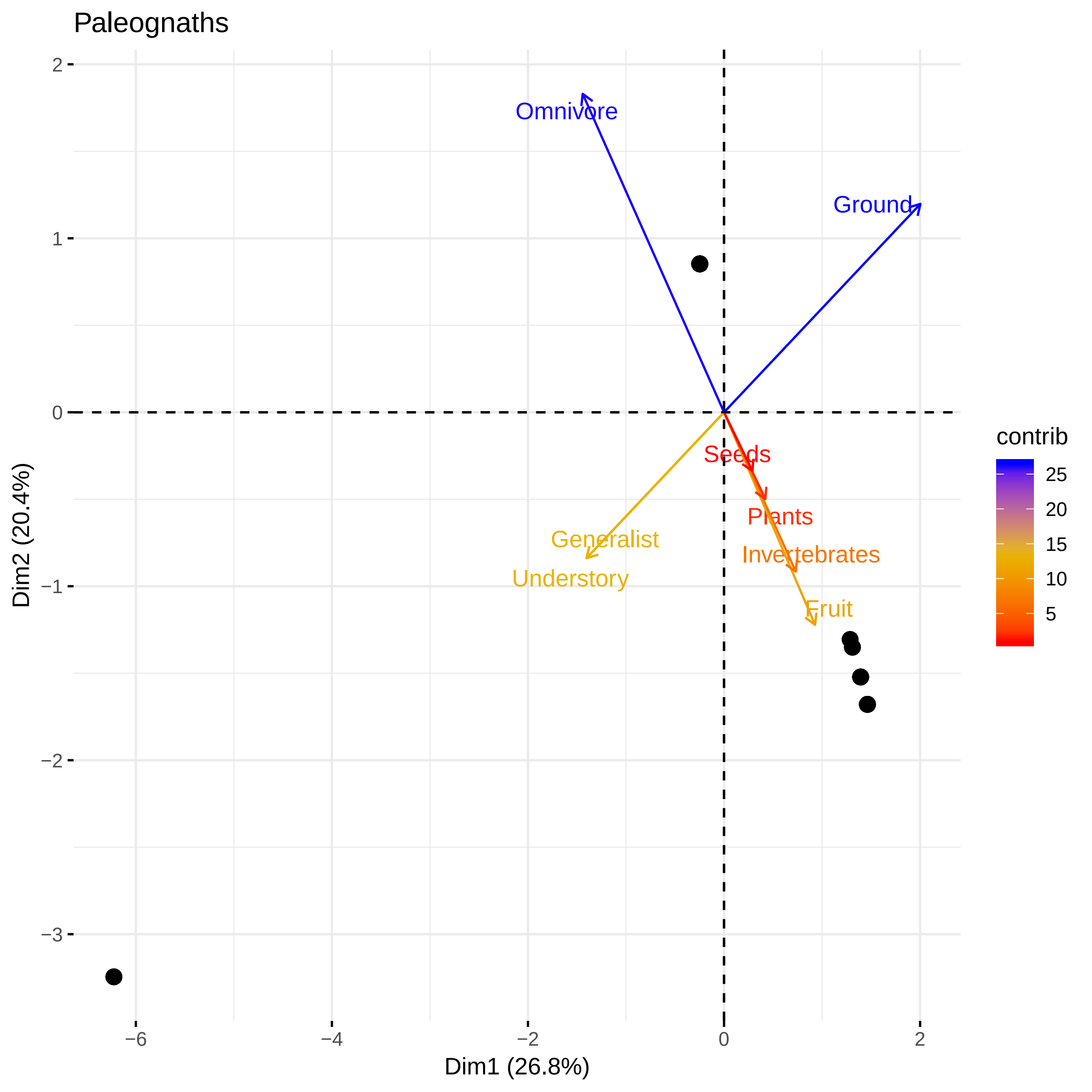
Paleognaths
Omnivore
Ground
Seeds
Plants
Generalist
Understory
Invertebrates
Fruit
contrib
25
20
15
10
5
2
1
0
−1
−2
−3
−6
−4
−2
0
2
Dim1 (26.8%)
Dim2 (20.4%)

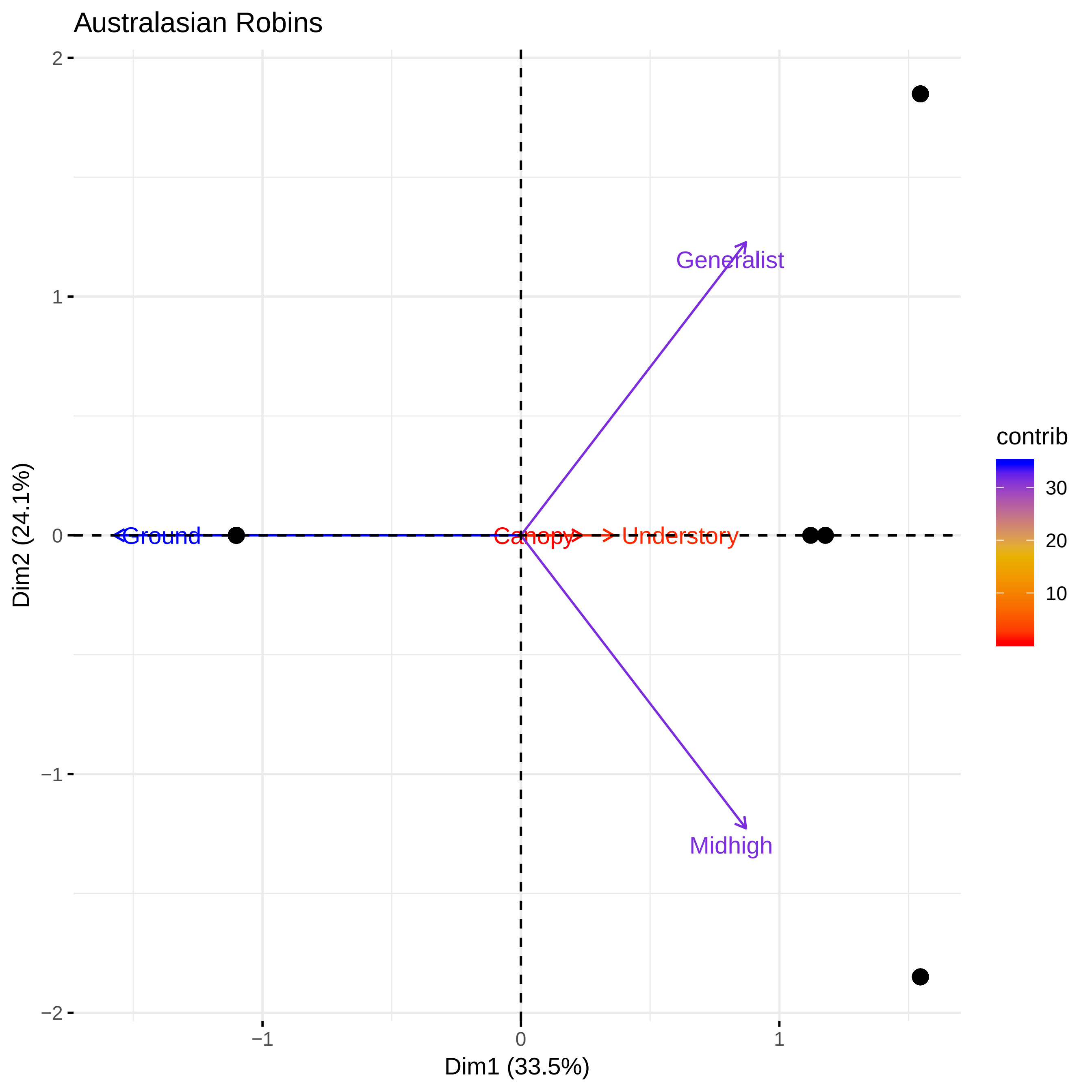

Australasian Robins
Generalist
Ground
Canopy
Understory
Midhigh
contrib
30
20
10
2
1
0
−1
−2
Dim2 (24.1%)
−1
0
1
Dim1 (33.5%)

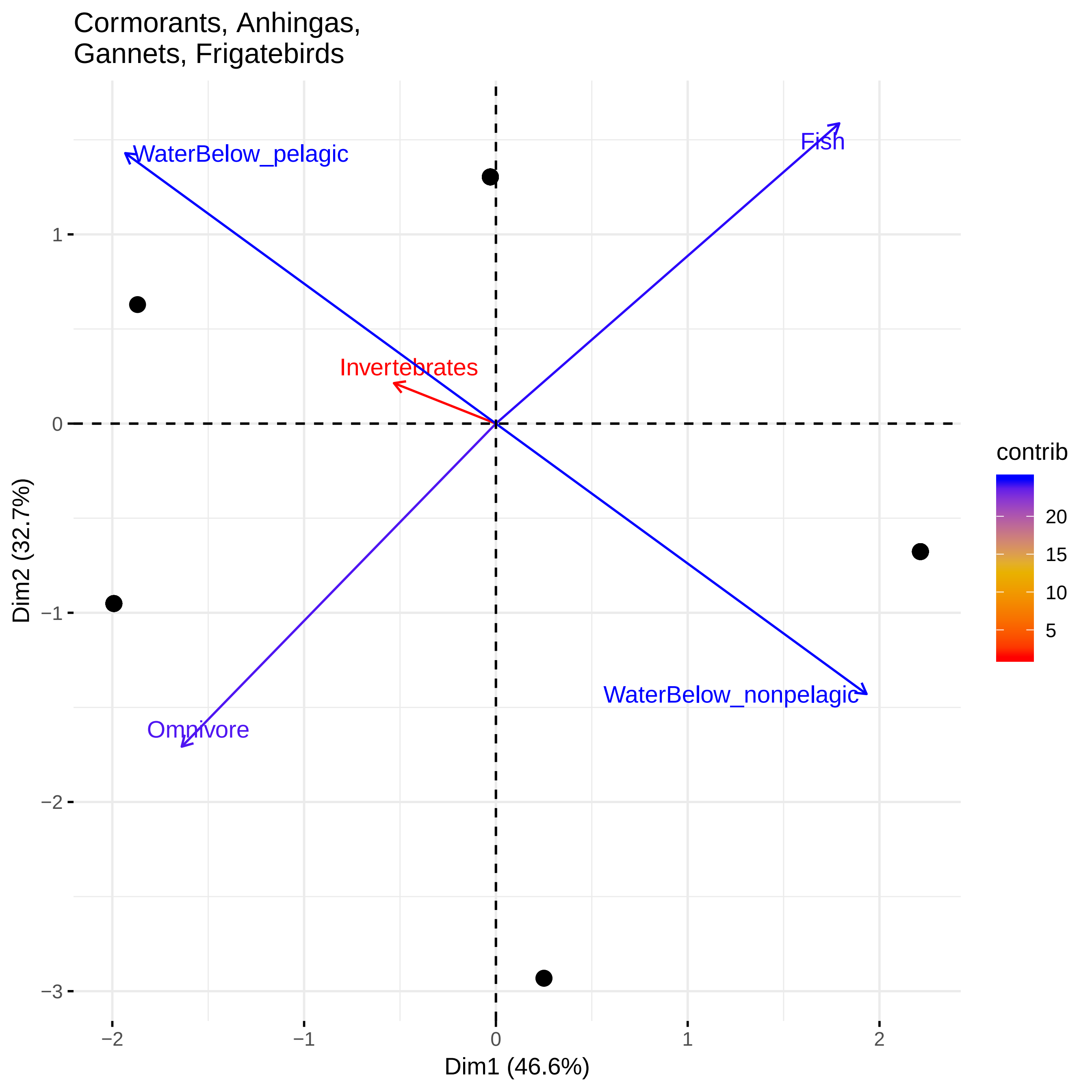

Cormorants, Anhingas,
Gannets, Frigatebirds
WaterBelow_pelagic
Fish
Invertebrates
WaterBelow_nonpelagic
Omnivore
contrib
20
15
10
5
1
0
−1
−2
−3
−2
−1
0
1
2
Dim1 (46.6%)
Dim2 (32.7%)

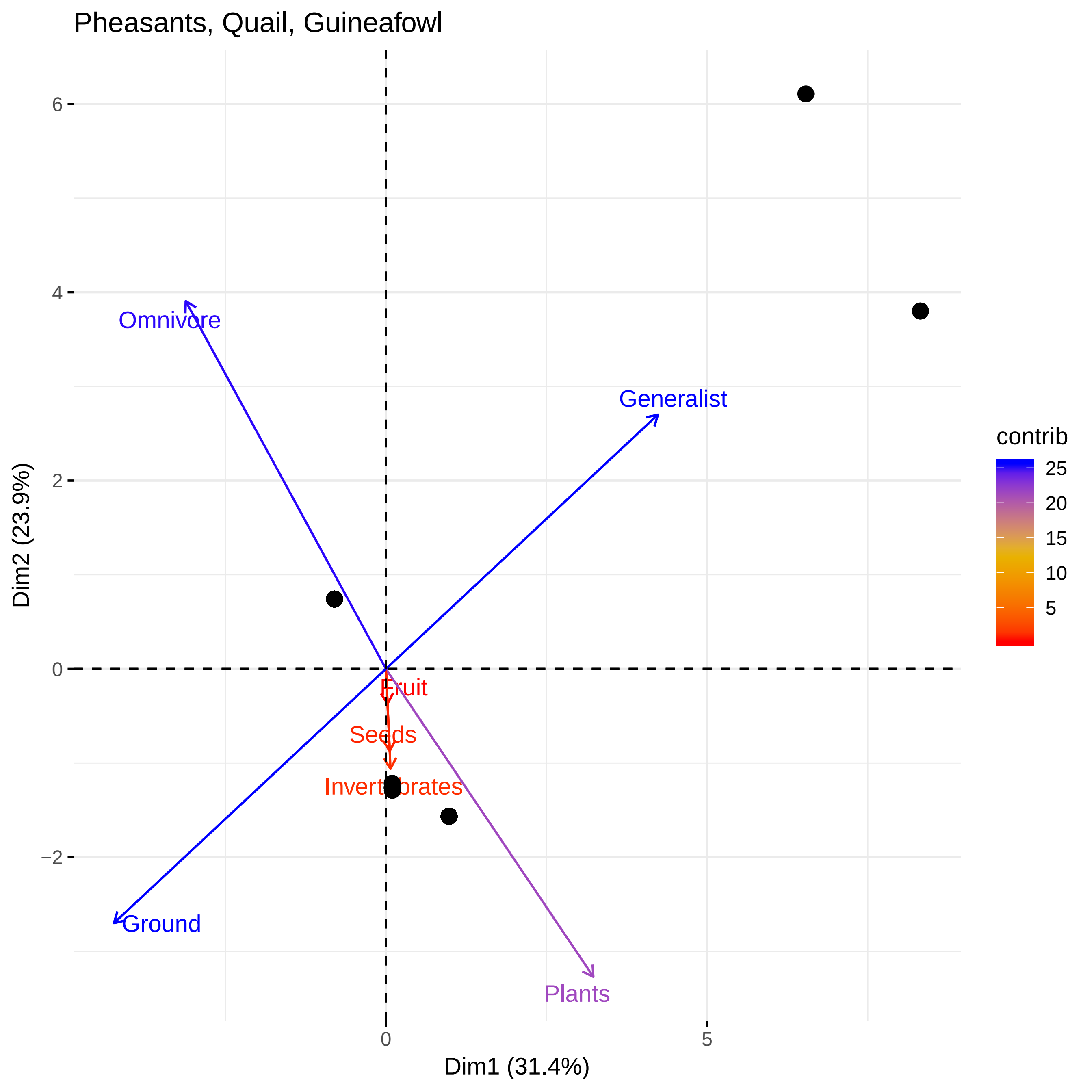

Pheasants, Quail, Guineafowl
Omnivore
Generalist
Fruit
Seeds
Invertebrates
Ground
Plants
contrib
25
20
15
10
5
6
4
2
0
−2
0
5
Dim1 (31.4%)
Dim2 (23.9%)

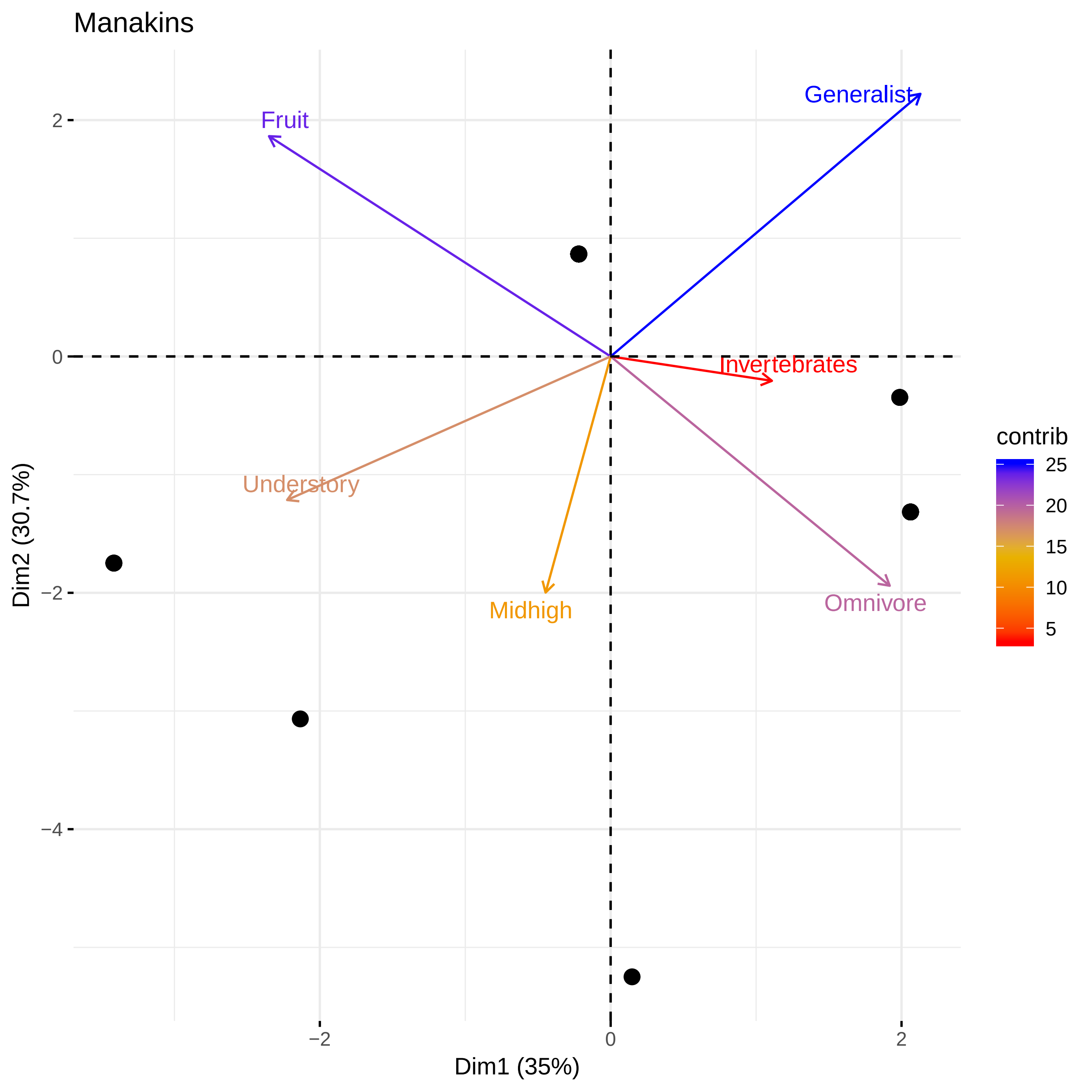
Manakins
Generalist
Fruit
Invertebrates
Understory
Midhigh
Omnivore
contrib
25
20
15
10
5
2
0
−2
−4
Dim2 (30.7%)
Dim1 (35%)

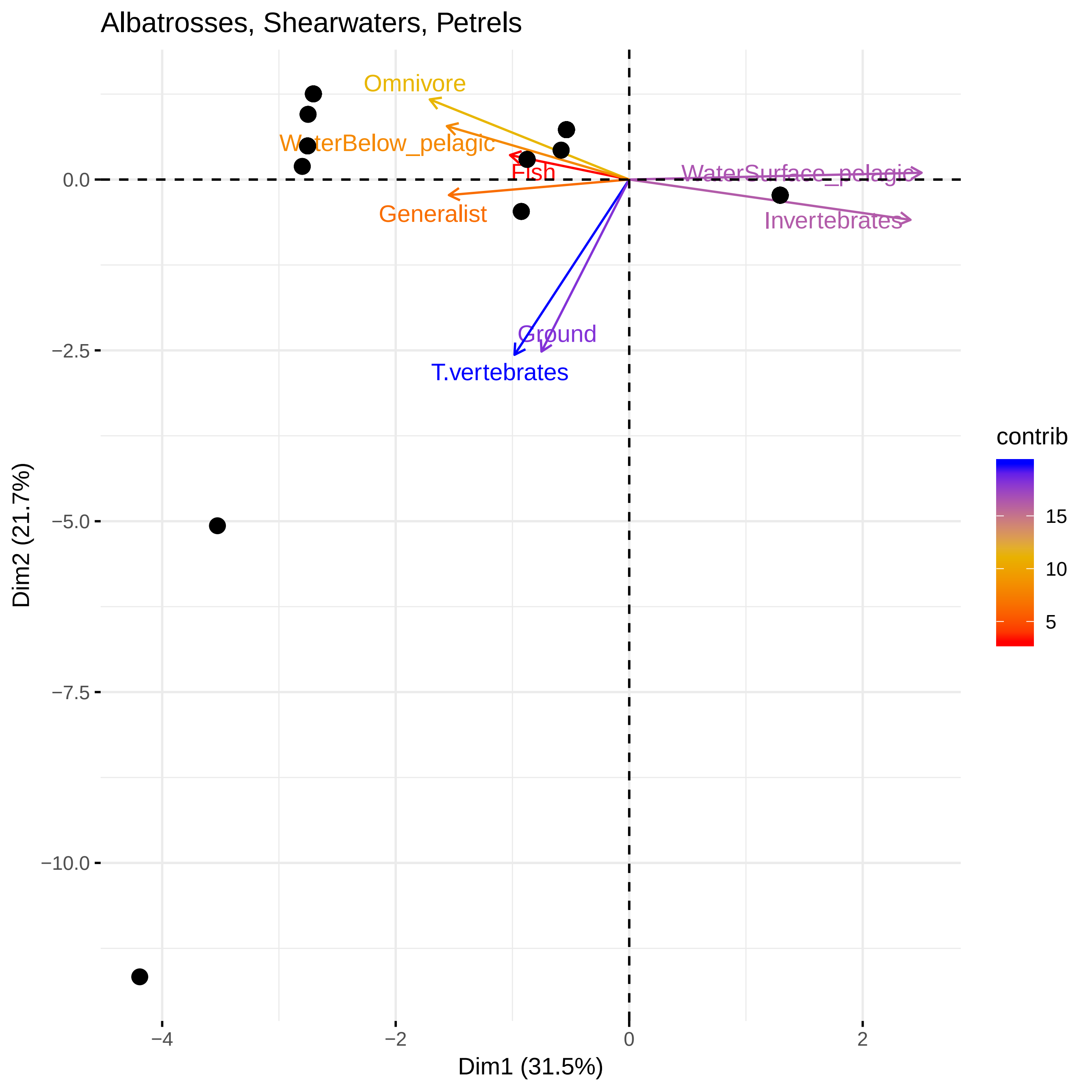

Albatrosses, Shearwaters, Petrels
Omnivore
WaterBelow_pelagic
Fish
Generalist
WaterSurface_pelagic
Invertebrates
Ground
T.vertebrates
contrib
15
10
5
0.0
−2.5
−5.0
−7.5
−10.0
−4
−2
0
2
Dim1 (31.5%)
Dim2 (21.7%)

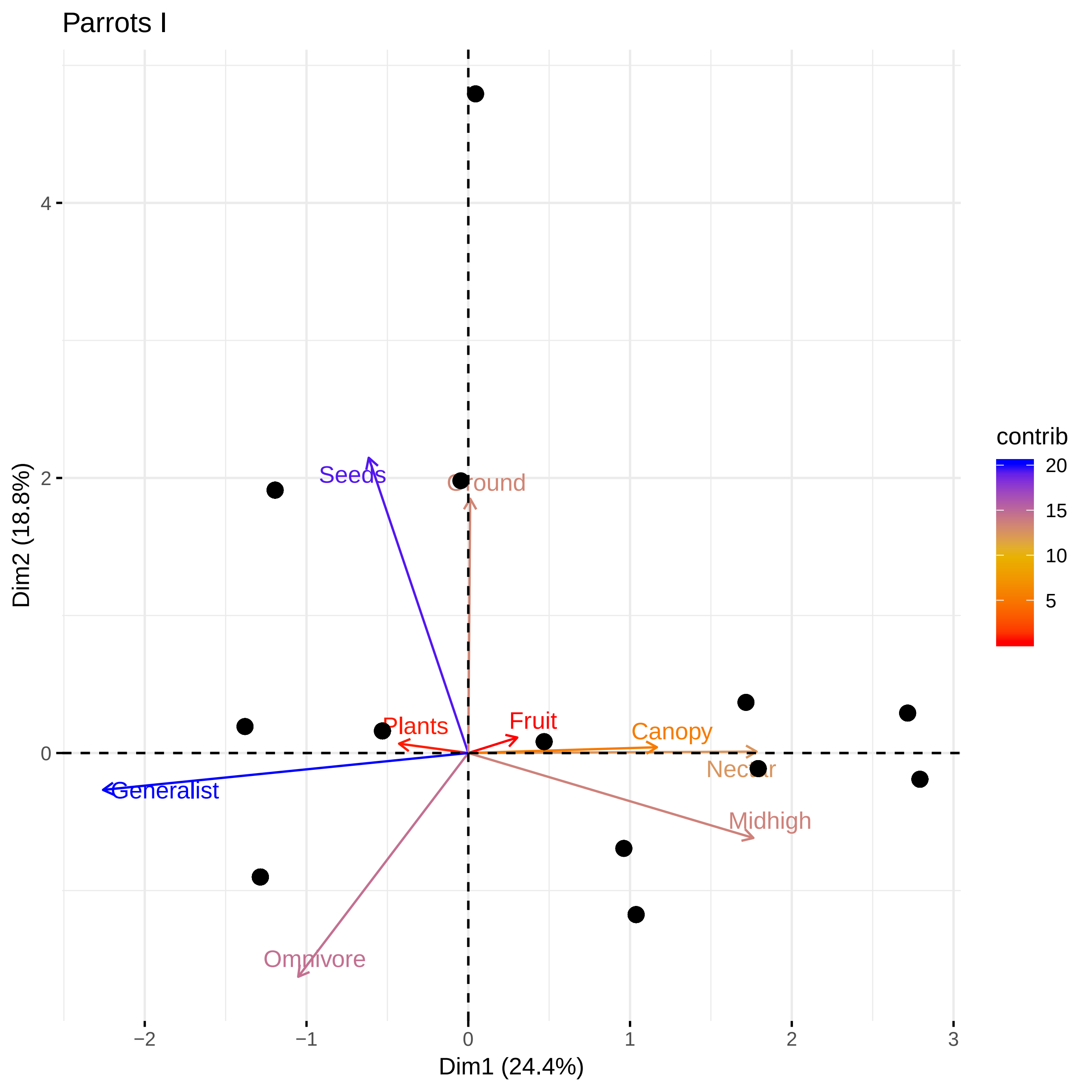
Parrots I
Seeds
Ground
Plants
Fruit
Canopy
Nectar
Generalist
Midhigh
Omnivore
Dim1 (24.4%)
Dim2 (18.8%)
contrib
20
15
10
5
−2
−1
0
1
2
3
4
2
0

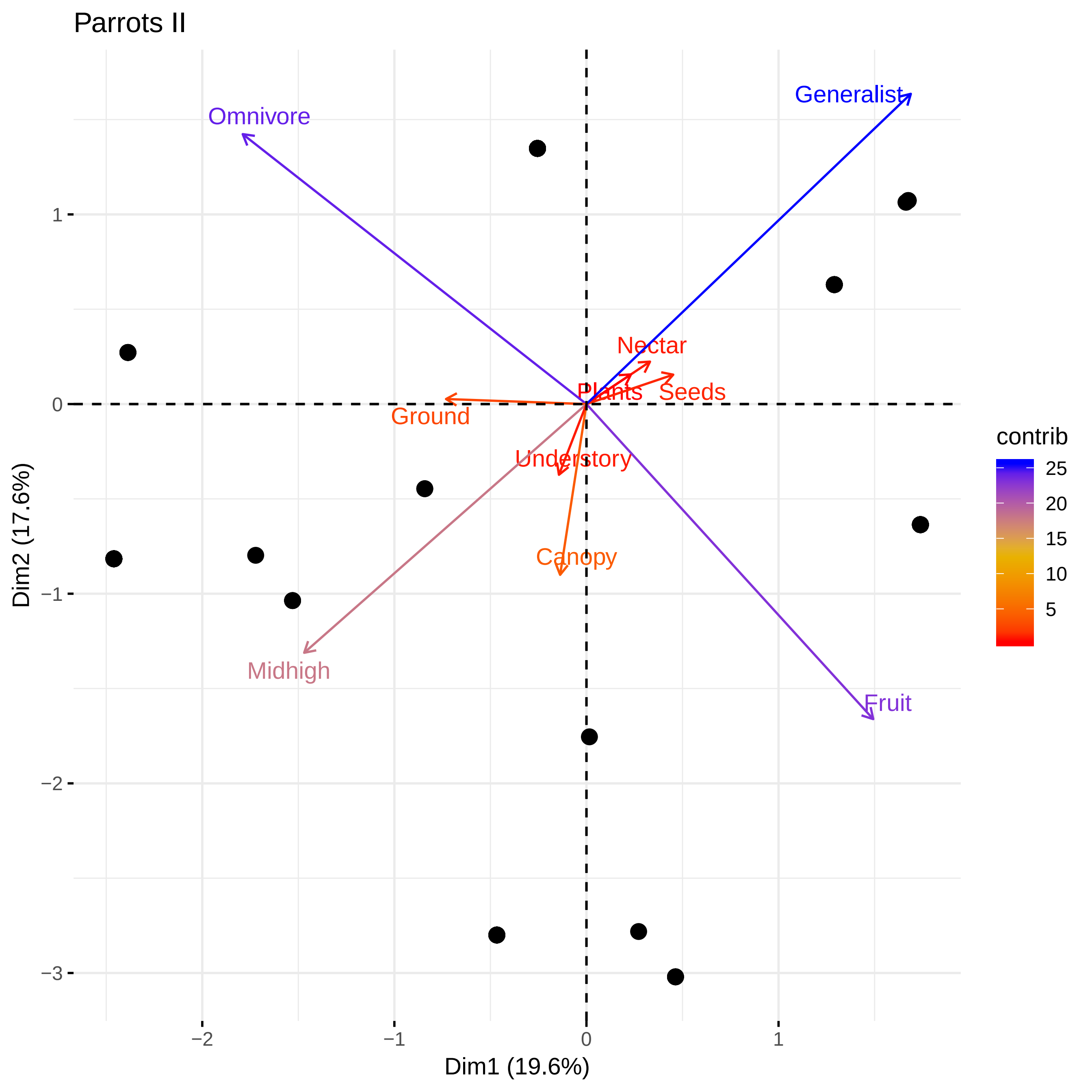

Parrots II
Generalist
Omnivore
Nectar
Plants
Seeds
Ground
Understory
Canopy
Midhigh
Fruit
contrib
25
20
15
10
5
1
0
−1
−2
−3
−2
−1
0
1
Dim1 (19.6%)
Dim2 (17.6%)

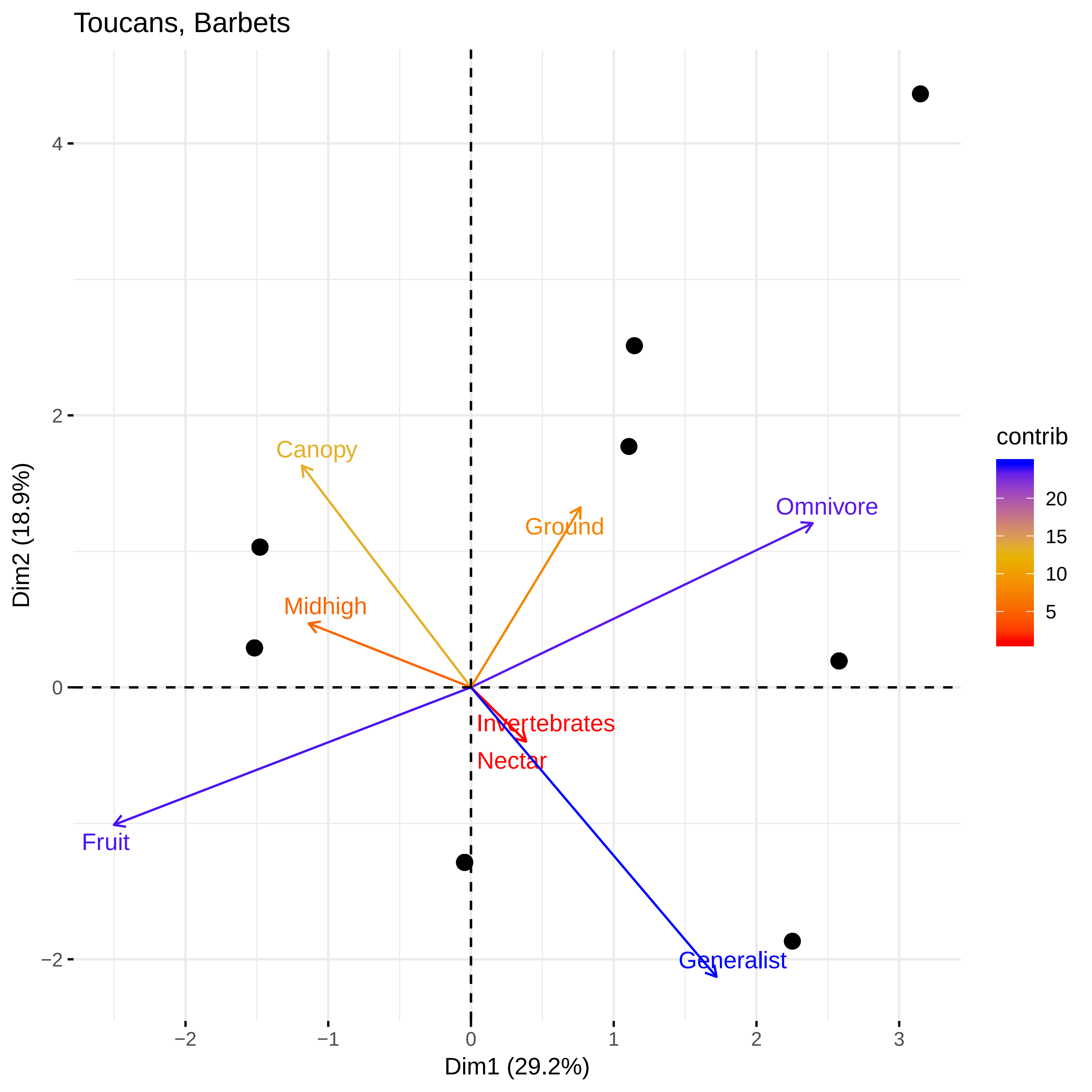
Toucans, Barbets
Canopy
Ground
Omnivore
Midhigh
Invertebrates
Nectar
Fruit
Generalist
contrib
20
15
10
5
4
2
0
−2
−2
−1
0
1
2
3
Dim1 (29.2%)
Dim2 (18.9%)

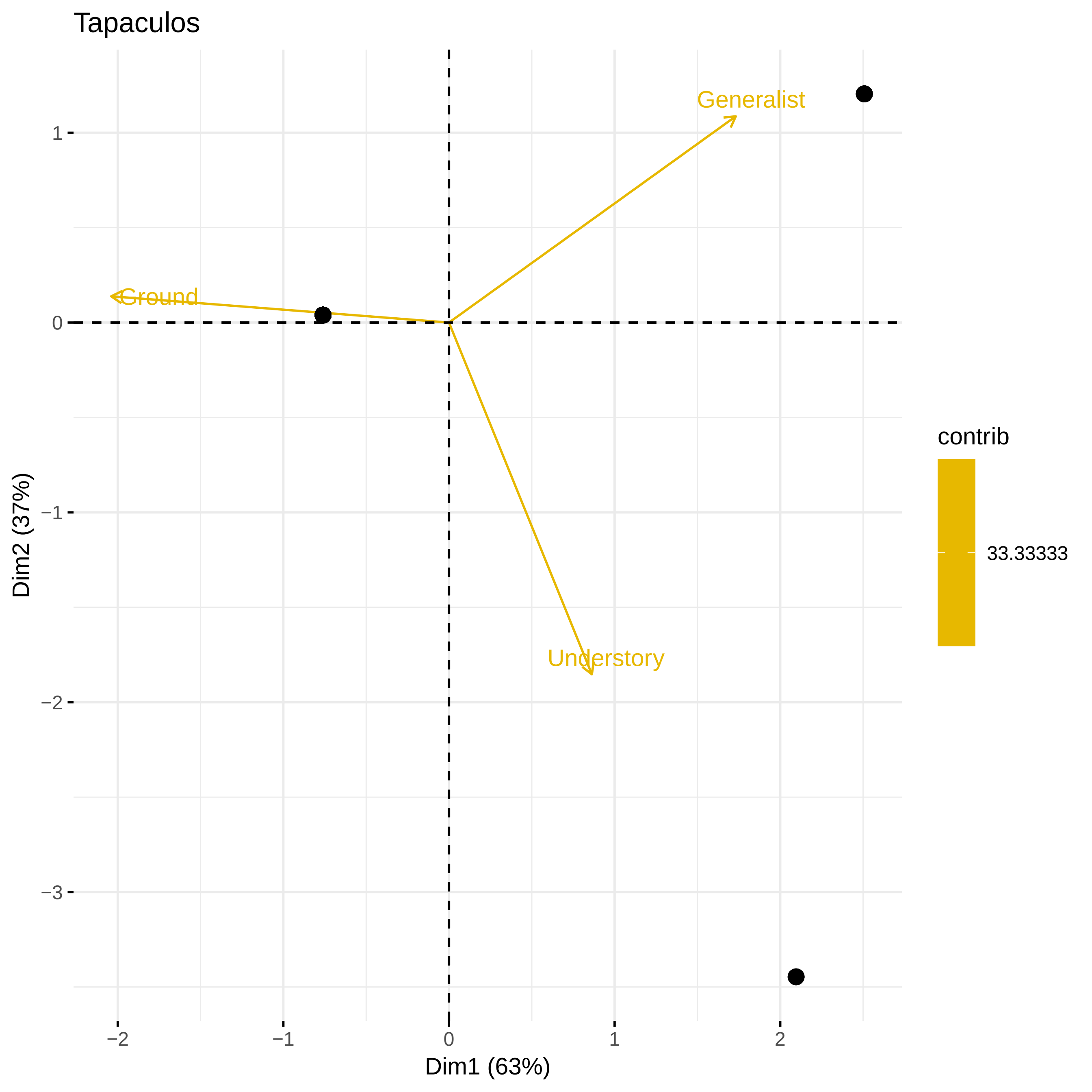
Tapaculos
Generalist
Ground
Understory
contrib
33.33333
Dim1 (63%)
Dim2 (37%)
−2
−1
0
1
2
−3

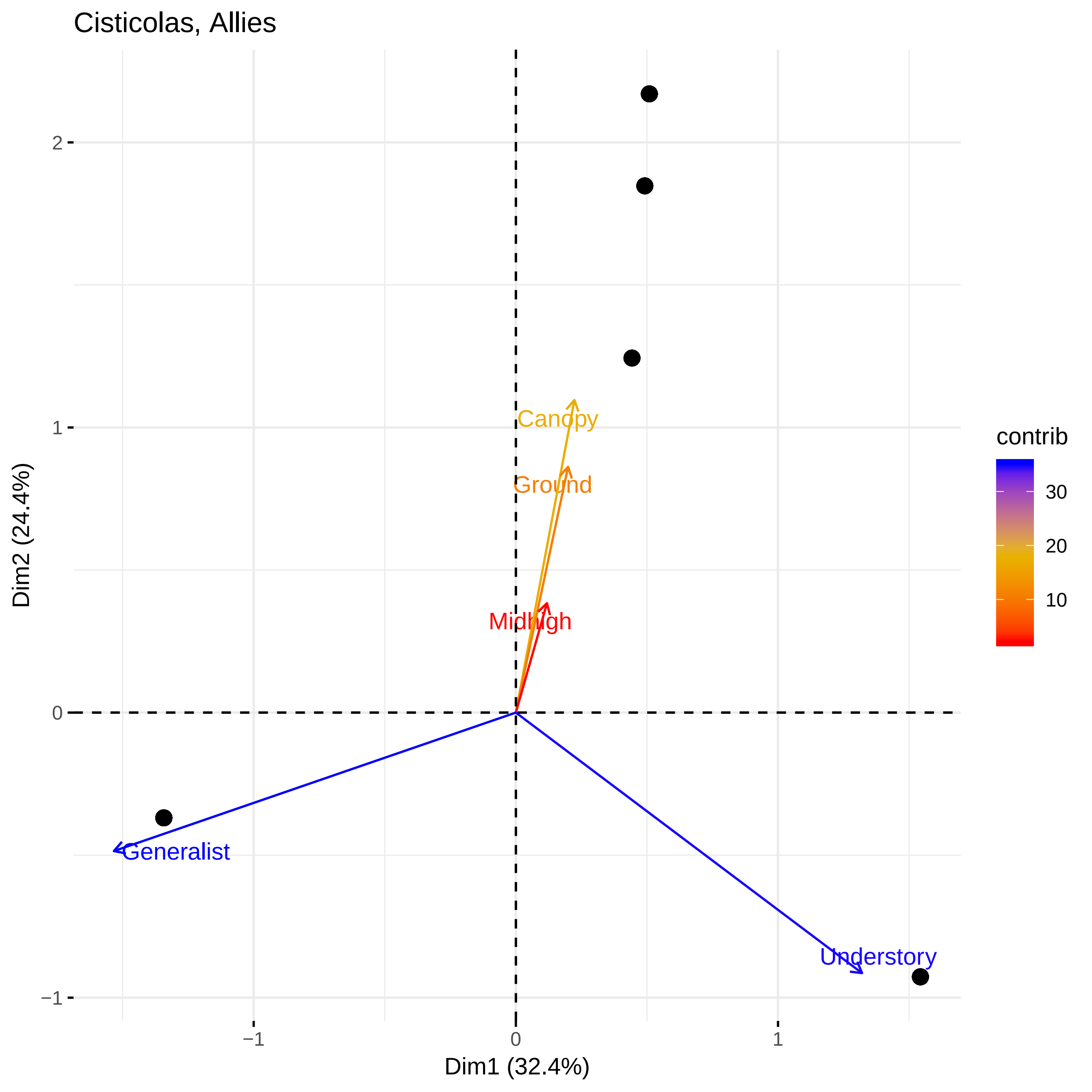
Cisticolas, Allies
Canopy
Ground
Midhigh
Generalist
Understory
contrib
30
20
10
2
1
0
−1
−1
0
1
Dim2 (24.4%)
Dim1 (32.4%)

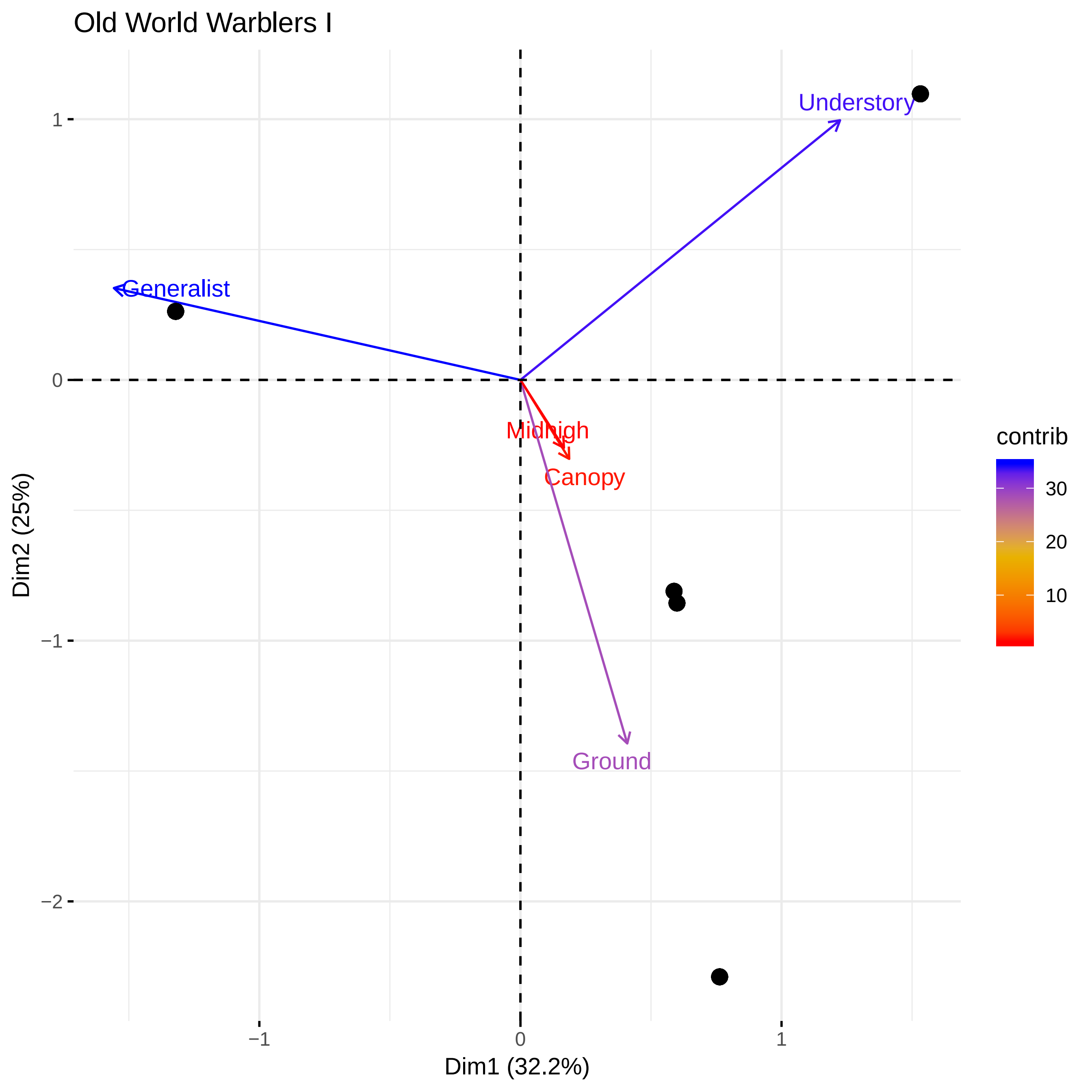

Old World Warblers I
Understory
Generalist
Midhigh
Canopy
Ground
contrib
30
20
10
1
0
−1
−2
−1
0
1
Dim1 (32.2%)
Dim2 (25%)

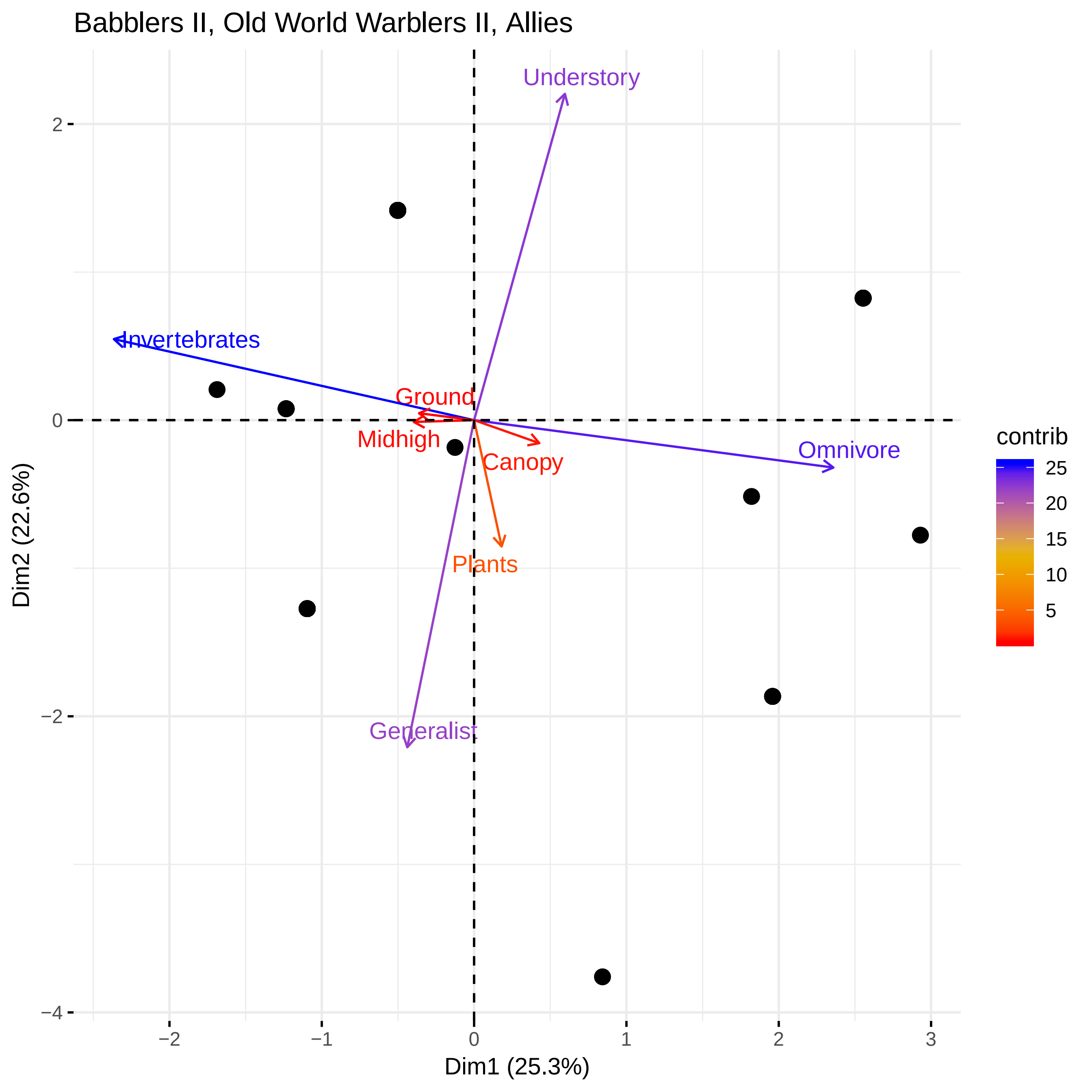

Babblers II, Old World Warblers II, Allies
Understory
Invertebrates
Ground
Midhigh
Canopy
Omnivore
Plants
Generalist
contrib
25
20
15
10
5
2
0
−2
−4
−2
−1
0
1
2
3
Dim1 (25.3%)
Dim2 (22.6%)

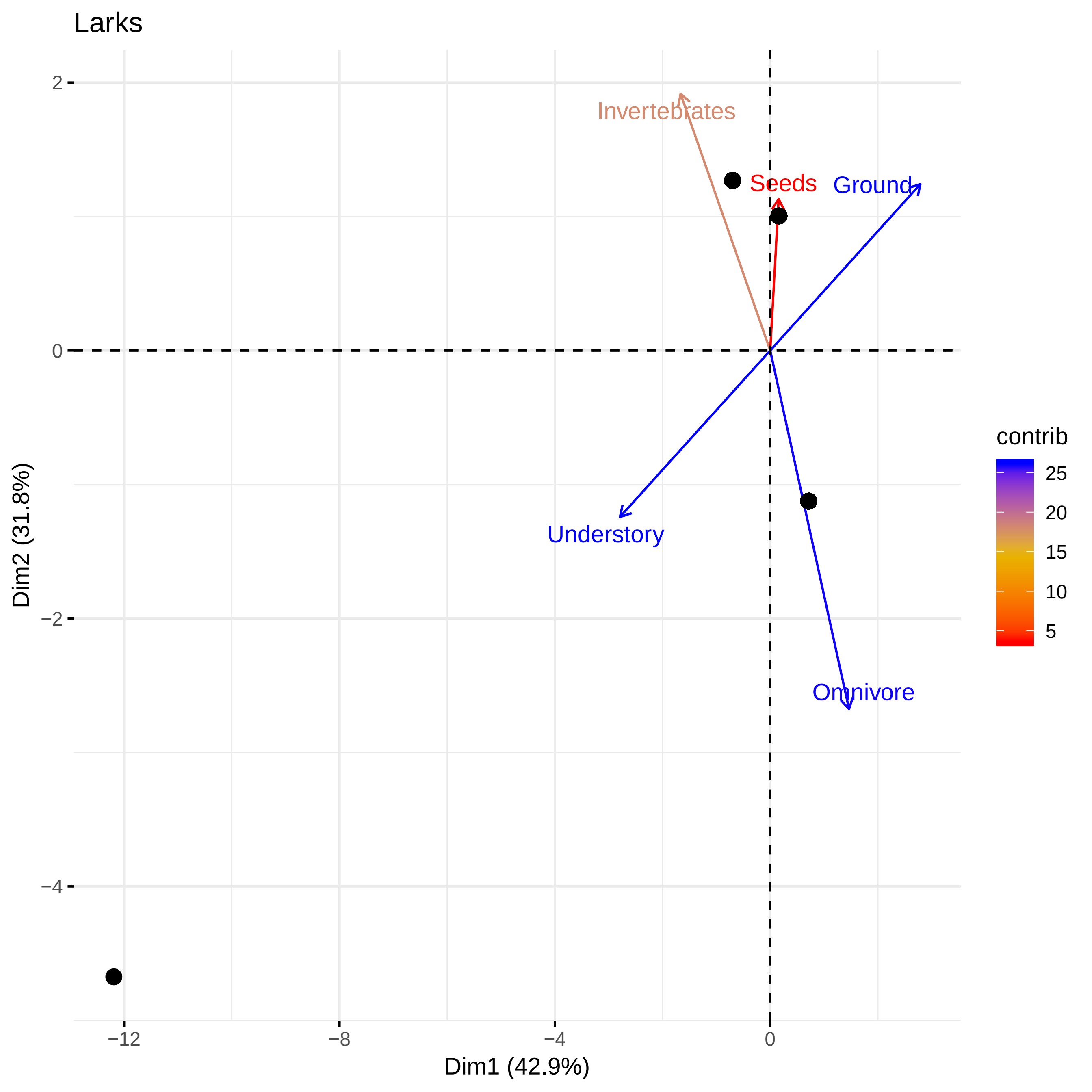

Larks
Invertebrates
Seeds
Ground
Understory
Omnivore
contrib
25
20
15
10
5
2
0
−2
−4
−12
−8
−4
0
Dim1 (42.9%)
Dim2 (31.8%)

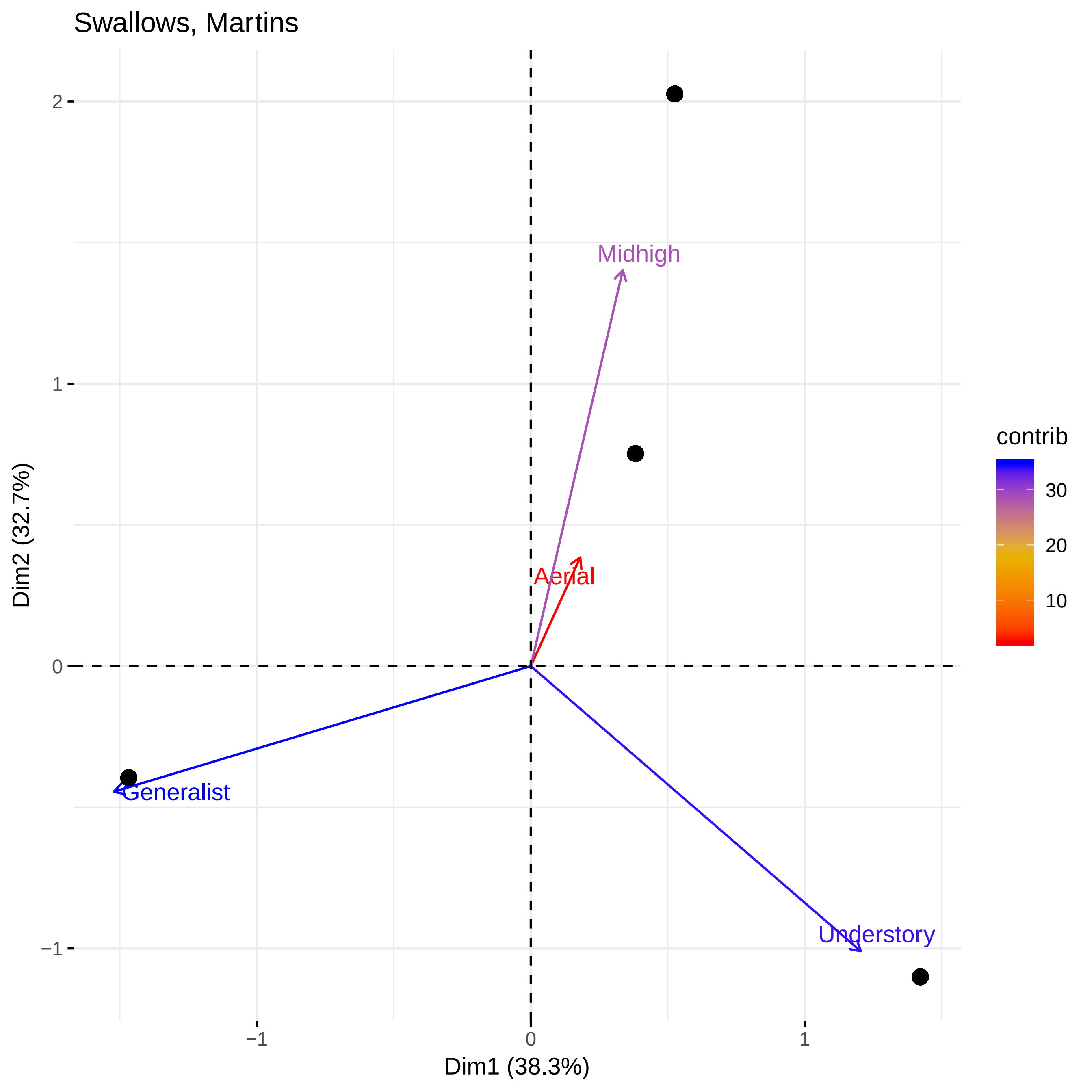

Swallows, Martins
Midhigh
Aerial
Generalist
Understory
contrib
30
20
10
Dim2 (32.7%)
Dim1 (38.3%)
2
1
0
−1
−1
0
1

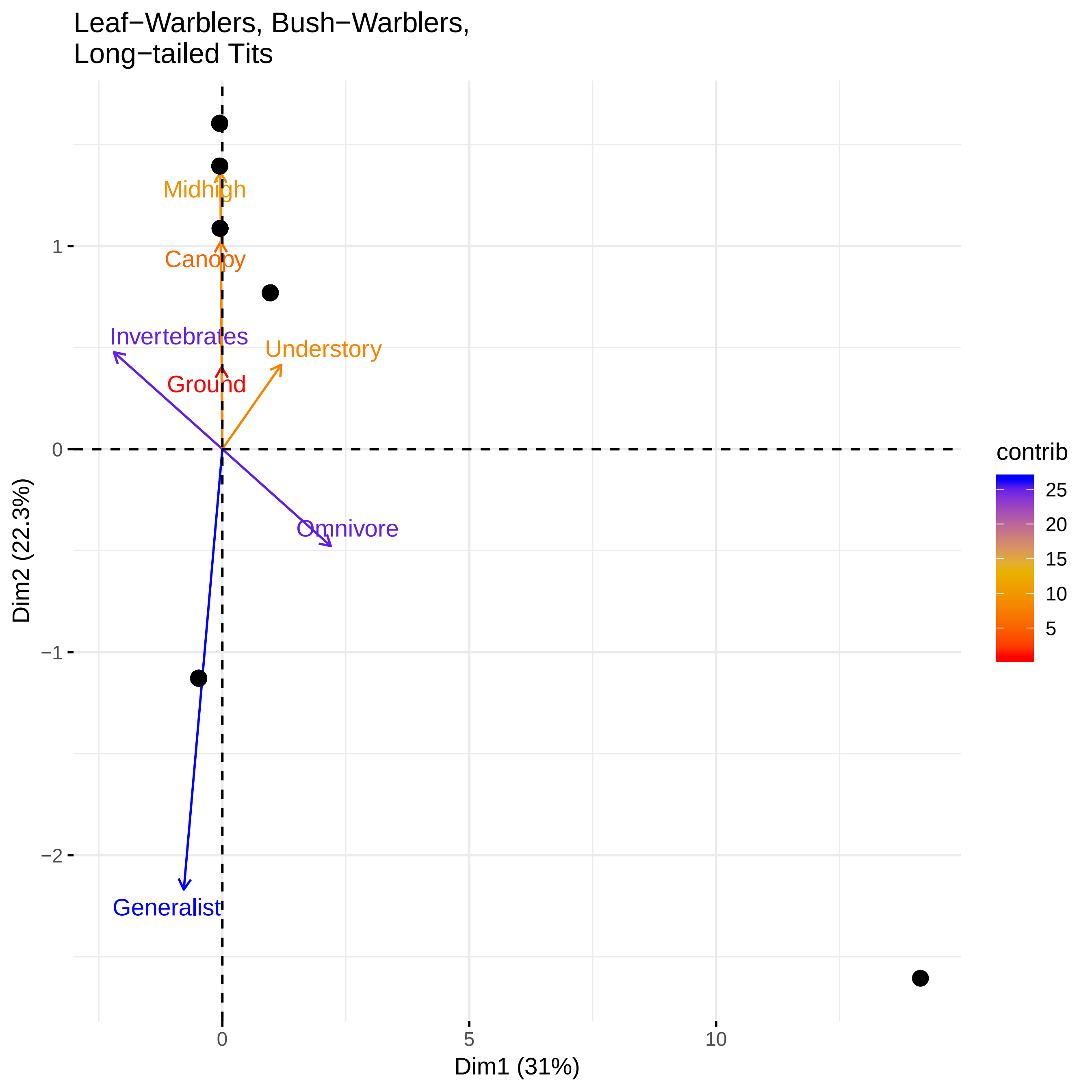
Leaf−Warblers, Bush−Warblers,
Long−tailed Tits
Midhigh
Canopy
Invertebrates
Understory
Ground
Omnivore
Generalist
contrib
25
20
15
10
5
1
0
−1
−2
0
5
10
Dim1 (31%)
Dim2 (22.3%)

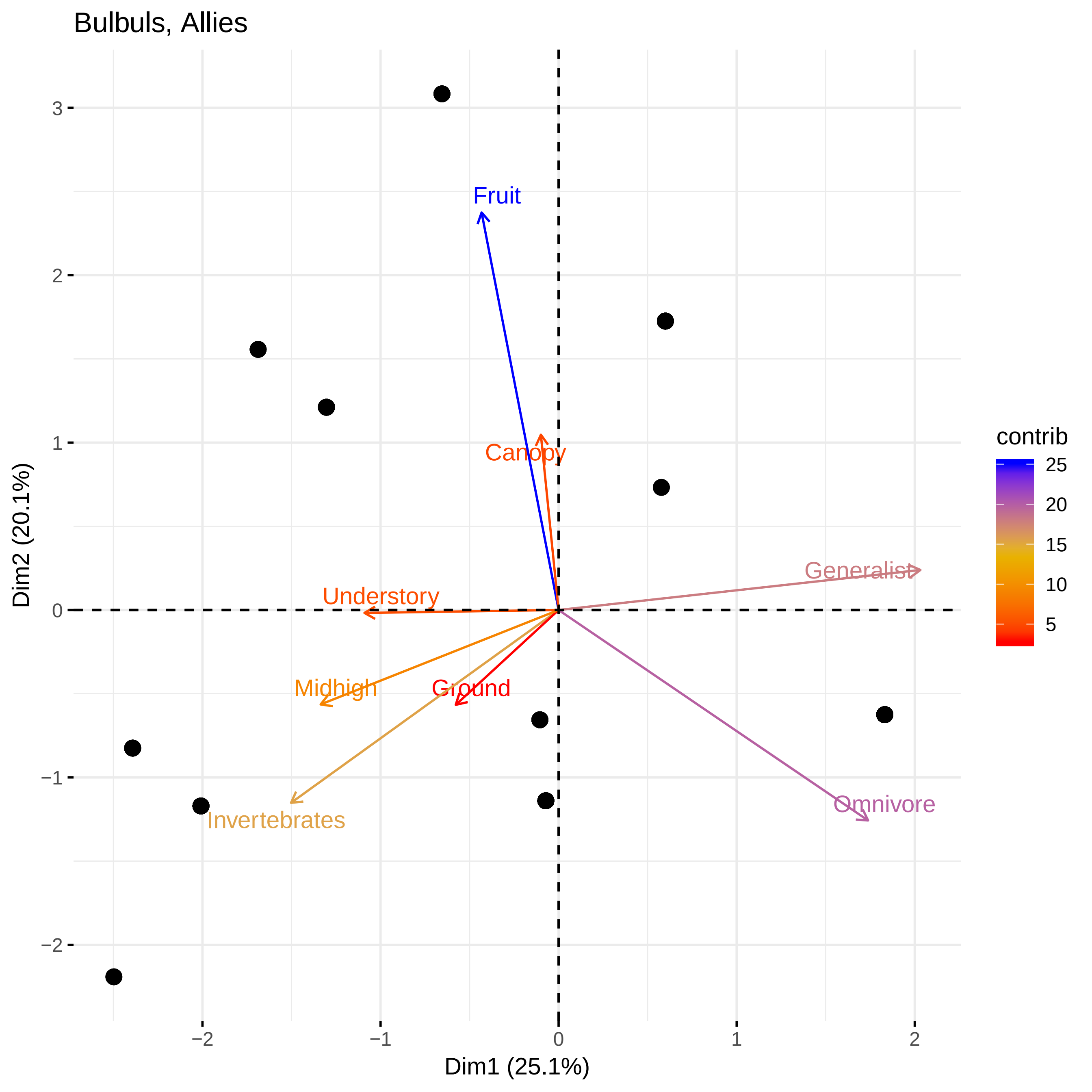

Bulbuls, Allies
Fruit
Canopy
Generalist
Understory
Midhigh
Ground
Invertebrates
Omnivore
contrib
25
20
15
10
5
3
2
1
0
−1
−2
Dim2 (20.1%)
Dim1 (25.1%)

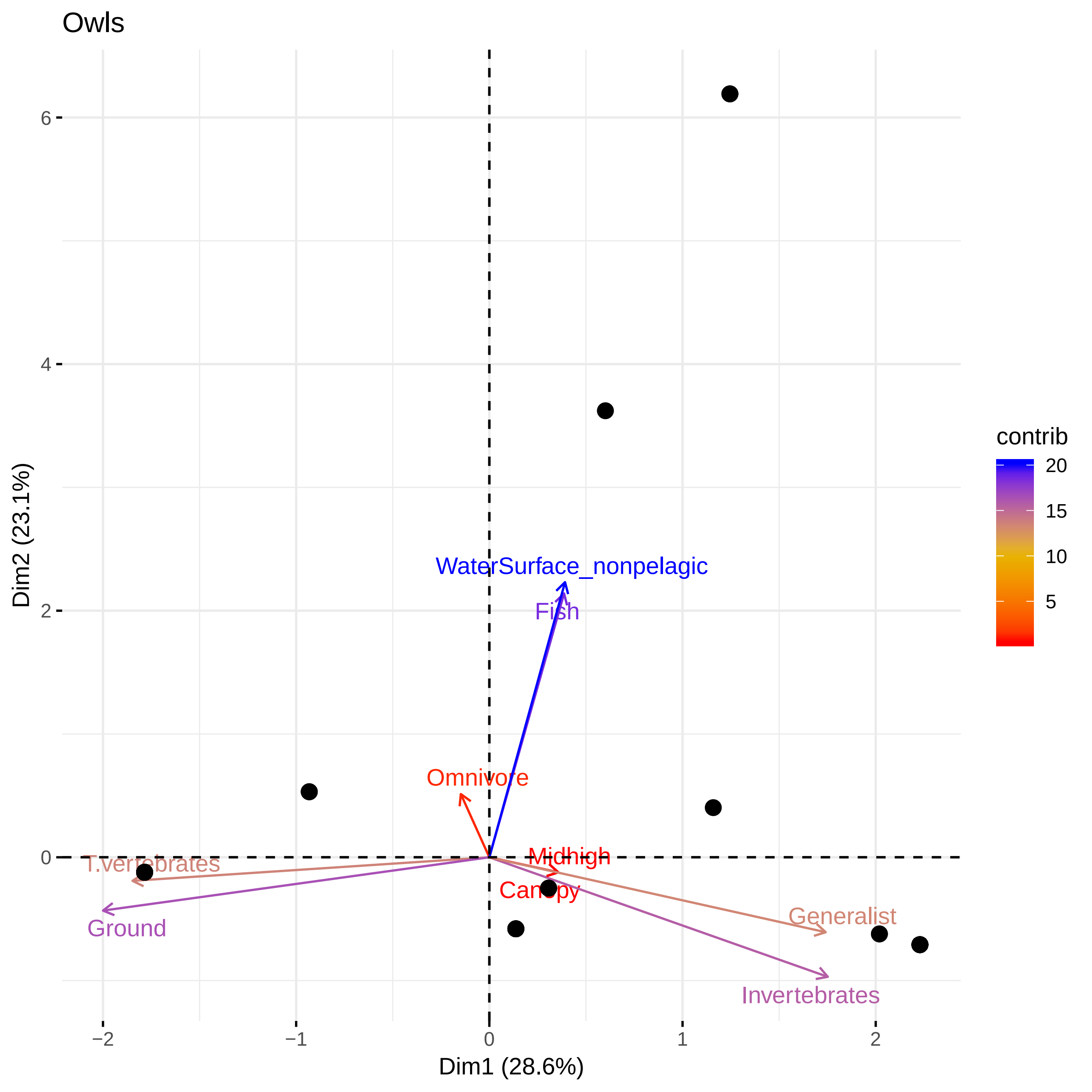
Owls
WaterSurface_nonpelagic
Fish
Omnivore
Midhigh
Canopy
T.vertebrates
Ground
Generalist
Invertebrates
contrib
20
15
10
5
6
4
2
0
−2
−1
0
1
2
Dim1 (28.6%)
Dim2 (23.1%)

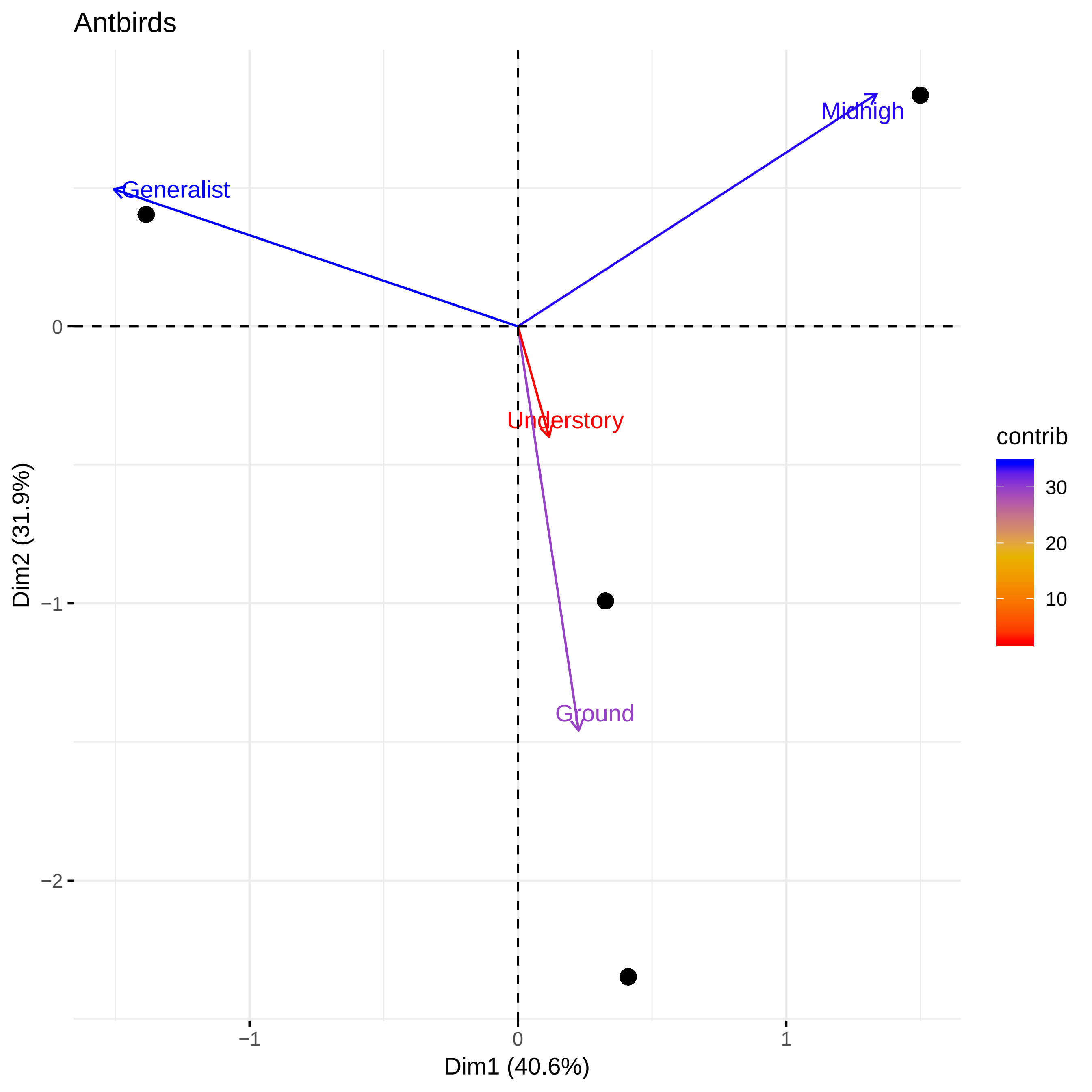

Antbirds
Midhigh
Generalist
Understory
Ground
0
−1
−2
−1
0
1
Dim1 (40.6%)
Dim2 (31.9%)
contrib
30
20
10

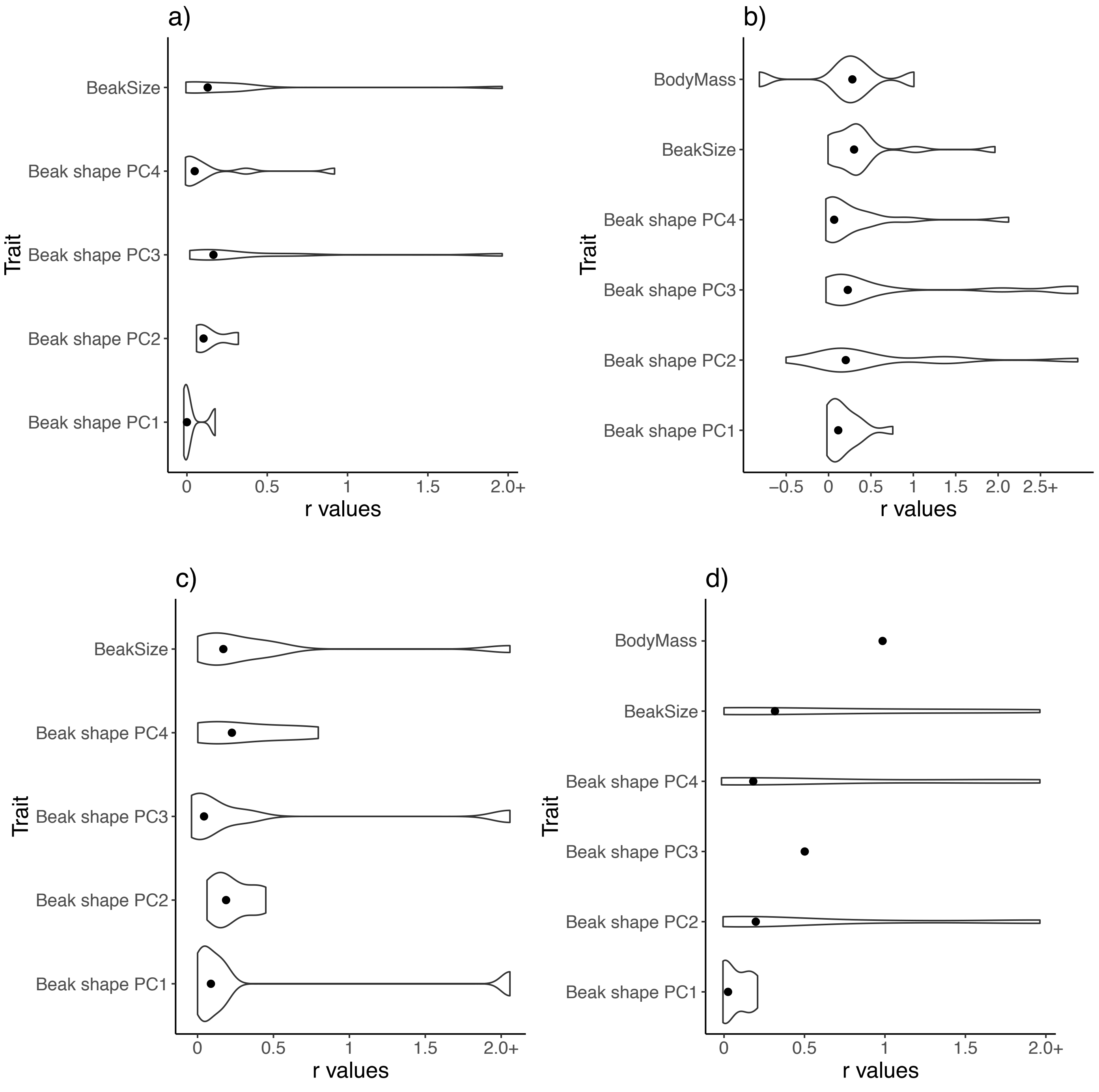

Figure S2. Violin plots showing the distributions of rate parameter values for exponential diversity-dependent models, applied (a) across deep-time scales, and (b) across recent radiations, (c) across genetic trees, and (d) across deep-time scales with biogeographic reconstructions. Positive r values indicate an exponential increase in trait evolutionary rates with the accumulation of species.

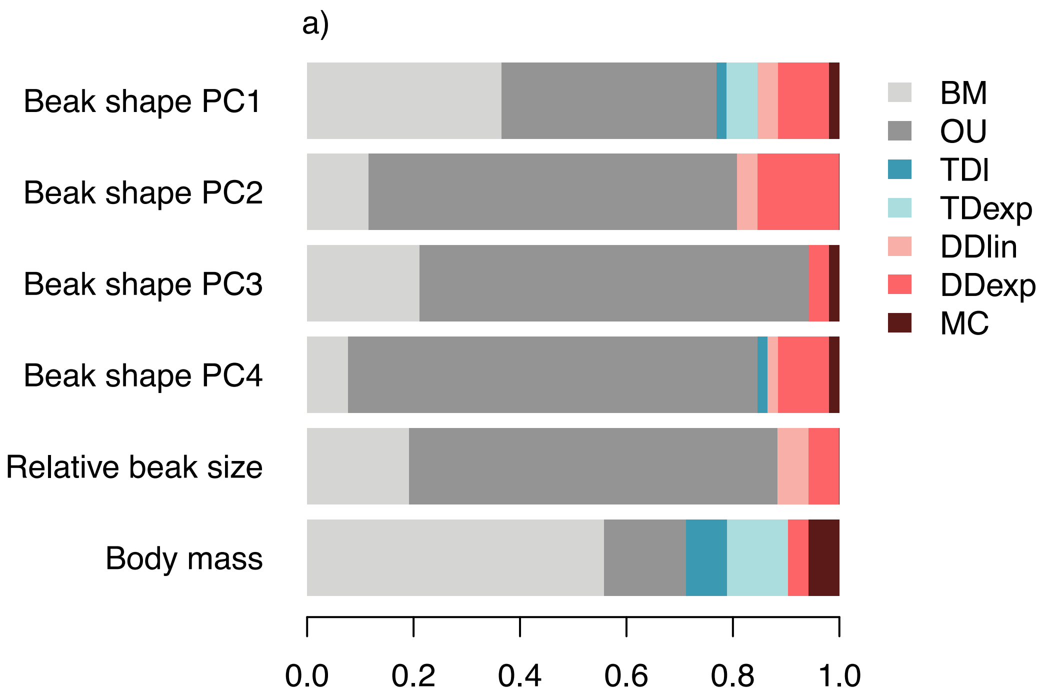

Figure S3. a) Model support (proportion of times each model is chosen as best i.e. smallest AICc values) across clades when modelling the evolution of various ecomorphological traits. We consider models without competition: Brownian motion (BM), Ornstein-Uhlenbeck (OU), linear (TDlin) and exponential (TDexp) time-dependent, and models with competition: linear (DDlin) and exponential (DDexp) diversity-dependent, and the matching competition (MC). Results summarized across avian orders and super-families trees used in models with biogeographic reconstructions (52 clades). (Overleaf) b) The signature of competition across across 52 avian orders and super-families trees used in models with biogeographic reconstructions. Support for competition (purple) is determined by whether a diversity or trait dependent model explains the data best in the focal clade, with an AICc difference greater than two compared to any model assuming lineages evolve independently. The following ecomorphological traits are considered (left to right circles): beak shape (purple indicates signal of competition in either PC1, PC2, PC3, or PC4), relative beak size, and body mass. 14 out of 52 clades show support for competition in either beak shape, size, or body mass ("any trait" in the stacked bars). Clades where models with and without competition cannot be distinguished by an AICc difference greater than two are marked by light gray.

b)

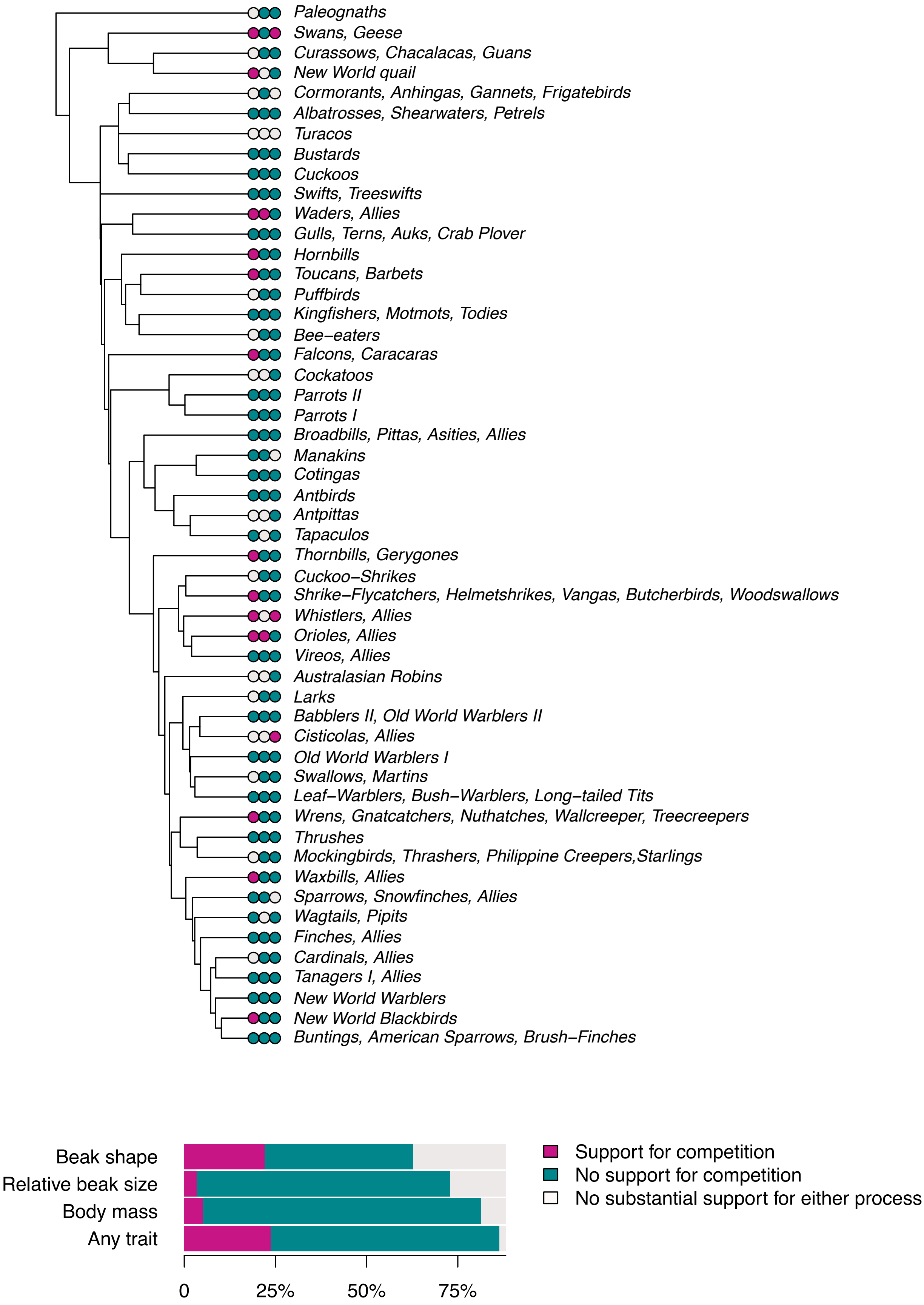
Paleognaths
Swans, Geese
Curassows, Chachalacas, Guans
New World quail
Cormorants, Anhingas, Gannets, Frigatebirds
Albatrosses, Shearwaters, Petrels
Turacos
Bustards
Cuckoos
Swifts, Treeswifts
Waders, Allies
Gulls, Terns, Auks, Crab Plover
Hornbills
Toucans, Barbets
Puffbirds
Kingfishers, Motmots, Todies
Bee–eaters
Falcons, Caracaras
Cockatoos
Parrots II
Parrots I
Broadbills, Pittas, Asities, Allies
Manakins
Cotingas
Antbirds
Antpittas
Tapaculos
Thornbills, Gerygones
Cuckoo–Shrikes
Shrike–Flycatchers, Helmetshrikes, Vangas, Butcherbirds, Woodswallows
Whistlers, Allies
Orioles, Allies
Vireos, Allies
Australasian Robins
Larks
Babblers II, Old World Warblers II
Cisticolas, Allies
Old World Warblers I
Swallows, Martins
Leaf–Warblers, Bush–Warblers, Long–tailed Tits
Wrens, Gnatcatchers, Nuthatches, Wallcreeper, Treecreepers
Thrushes
Mockingbirds, Thrashers, Philippine Creepers,Starlings
Waxbills, Allies
Sparrows, Snowfinches, Allies
Wagtails, Pipits
Finches, Allies
Cardinals, Allies
Tanagers I, Allies
New World Warblers
New World Blackbirds
Buntings, American Sparrows, Brush–Finches
Beak shape
Relative beak size
Body mass
Any trait
0
25%
50%
75%
Support for competition
No support for competition
No substantial support for either process

Figure S4 (Overleaf). The signature of competition across 93 avian clades. Support for competition (purple) is determined by whether a diversity or trait dependent model explains the data best in the focal clade, with an AICc difference greater than two compared to any model assuming lineages evolve independently. The following ecomorphological traits are considered (left to right circles): beak shape (purple indicates signal of competition in either PC1, PC2, PC3, or PC4), relative beak size, and body mass. 37 out of 93 clades show support for competition in either beak shape, size, or body mass ("any trait" in the stacked bars). Clades where models with and without competition cannot be distinguished by an AICc difference greater than two are marked by light gray. Clades are named using avian orders and super-families names, and, if clades represent recent radiations within orders and super-families, the node number of the most recent common ancestor for each radiation is marked.

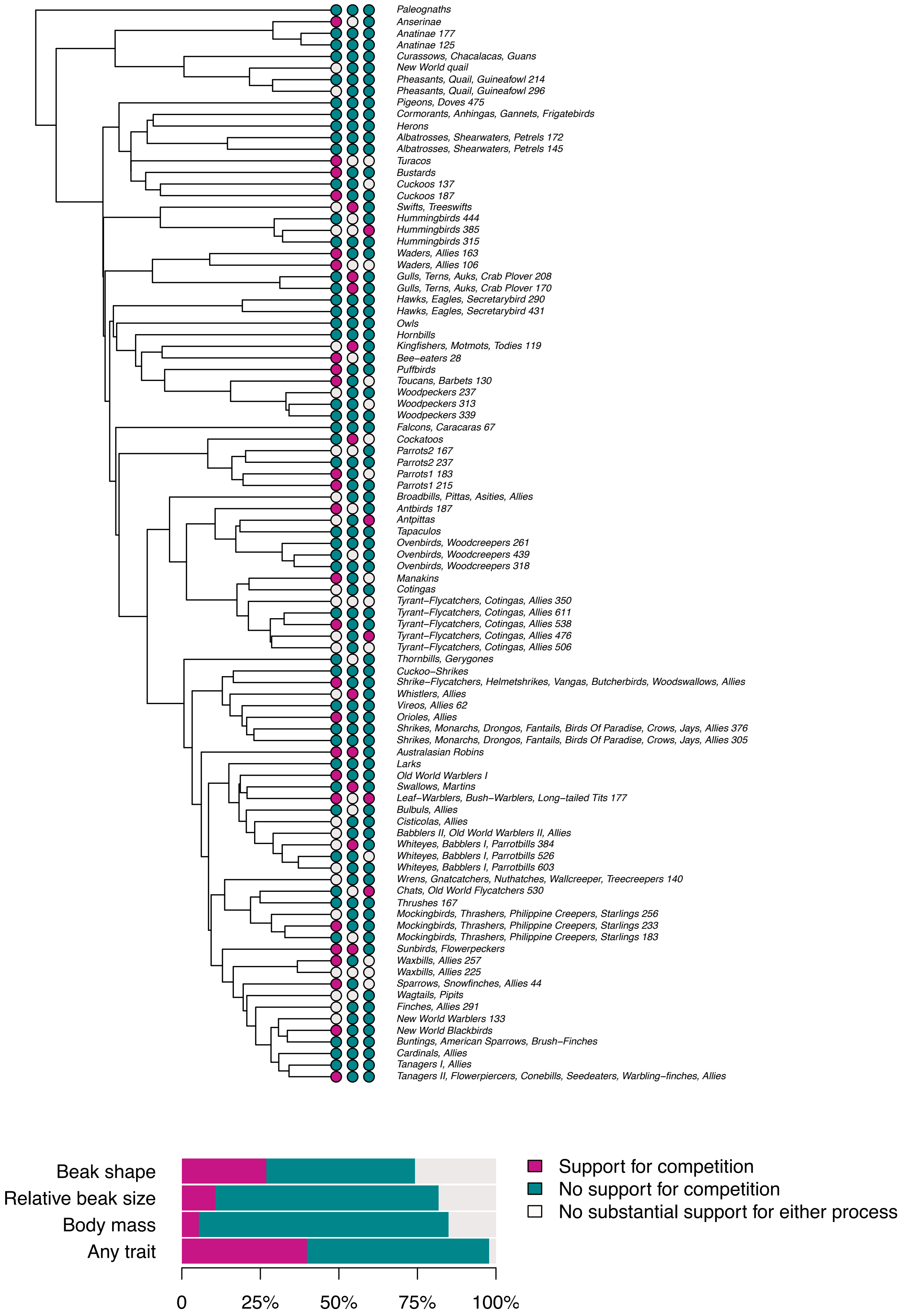
Paleognaths
Anserinae
Anatinae 177
Anatinae 125
Curassows, Chacalacas, Guans
New World quail
Pheasants, Quail, Guineafowl 214
Pheasants, Quail, Guineafowl 296
Pigeons, Doves 475
Cormorants, Anhingas, Gannets, Frigatebirds
Herons
Albatrosses, Shearwaters, Petrels 172
Albatrosses, Shearwaters, Petrels 145
Turacos
Bustards
Cuckoos 137
Cuckoos 187
Swifts, Treeswifts
Hummingbirds 444
Hummingbirds 385
Hummingbirds 315
Waders, Allies 163
Waders, Allies 106
Gulls, Terns, Auks, Crab Plover 208
Gulls, Terns, Auks, Crab Plover 170
Hawks, Eagles, Secretarybird 290
Hawks, Eagles, Secretarybird 431
Owls
Hornbills
Kingfishers, Motmots, Todies 119
Bee–eaters 28
Puffbirds
Toucans, Barbets 130
Woodpeckers 237
Woodpeckers 313
Woodpeckers 339
Falcons, Caracaras 67
Cockatoos
Parrots2 167
Parrots2 237
Parrots1 183
Parrots1 215
Broadbills, Pittas, Asities, Allies
Antbirds 187
Antpittas
Tapaculos
Ovenbirds, Woodcreepers 261
Ovenbirds, Woodcreepers 439
Ovenbirds, Woodcreepers 318
Manakins
Cotingas
Tyrant–Flycatchers, Cotingas, Allies 350
Tyrant–Flycatchers, Cotingas, Allies 611
Tyrant–Flycatchers, Cotingas, Allies 538
Tyrant–Flycatchers, Cotingas, Allies 476
Tyrant–Flycatchers, Cotingas, Allies 506
Thornbills, Gerygones
Cuckoo–Shrikes
Shrike–Flycatchers, Helmetshrikes, Vangas, Butcherbirds, Woodswallows, Allies
Whistlers, Allies
Vireos, Allies 62
Orioles, Allies
Shrikes, Monarchs, Drongos, Fantails, Birds Of Paradise, Crows, Jays, Allies 376
Shrikes, Monarchs, Drongos, Fantails, Birds Of Paradise, Crows, Jays, Allies 305
Australasian Robins
Larks
Old World Warblers I
Swallows, Martins
Leaf–Warblers, Bush–Warblers, Long–tailed Tits 177
Bulbuls, Allies
Cisticolas, Allies
Babblers II, Old World Warblers II, Allies
Whiteyes, Babblers I, Parrotbills 384
Whiteyes, Babblers I, Parrotbills 526
Whiteyes, Babblers I, Parrotbills 603
Wrens, Gnatcatchers, Nuthatches, Wallcreeper, Treecreepers 140
Chats, Old World Flycatchers 530
Thrushes 167
Mockingbirds, Thrashers, Philippine Creepers, Starlings 256
Mockingbirds, Thrashers, Philippine Creepers, Starlings 233
Mockingbirds, Thrashers, Philippine Creepers, Starlings 183
Sunbirds, Flowerpeckers
Waxbills, Allies 257
Waxbills, Allies 225
Sparrows, Snowfinches, Allies 44
Wagtails, Pipits
Finches, Allies 291
New World Warblers 133
New World Blackbirds
Buntings, American Sparrows, Brush–Finches
Cardinals, Allies
Tanagers I, Allies
Tanagers II, Flowerpiercers, Conebills, Seedeaters, Warbling–finches, Allies
Beak shape
Relative beak size
Body mass
Any trait
0
25%
50%
75%
100%
Support for competition
No support for competition
No substantial support for either process

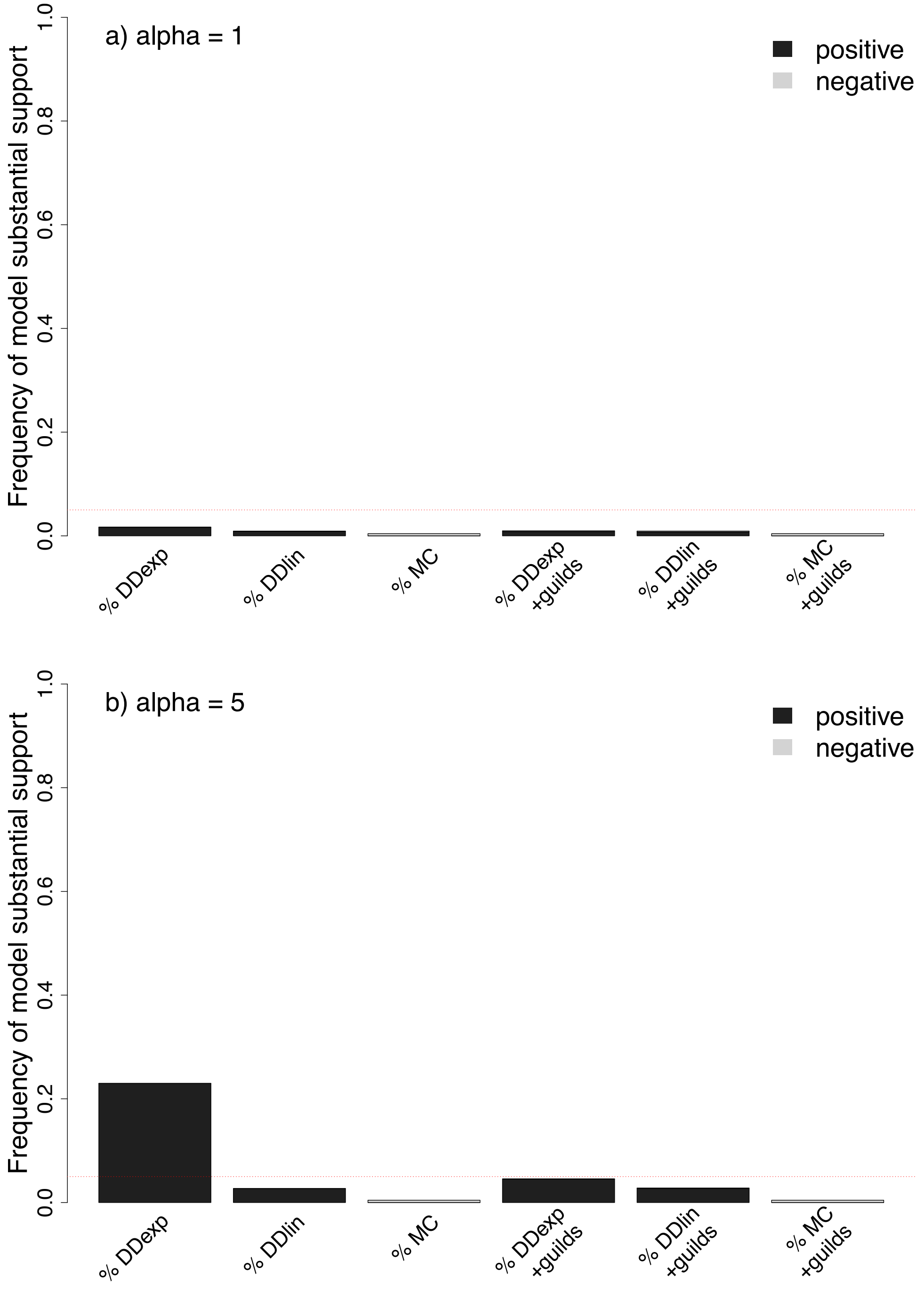

Figure S5. False positives error quantified as substantial support for models with competition (an AICc value smaller at a difference of two units from the AICc of the OU model, simulated with a) alpha = 1, and b) alpha = 5). The dashed line indicates the 5% value. Positive values for the r parameter in diversity-dependent models and S parameter in the matching competition model are indicated by a dark gray colour, whereas negative values of the parameters are indicated by light gray colours. Results summarized across trees of various sizes (25, 51, 112, 195 species).

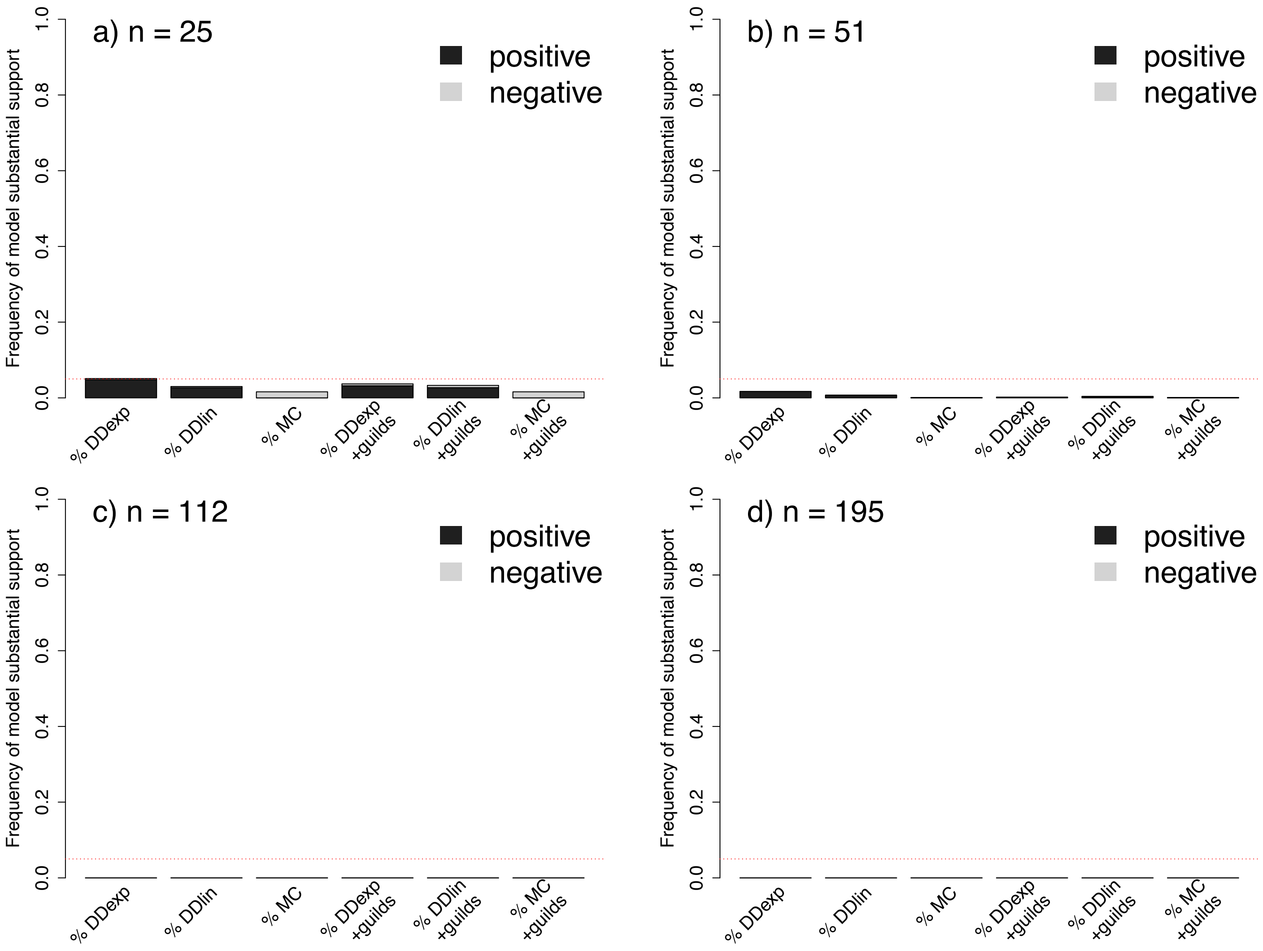

Figure S6. False positives error quantified as substantial support for models with competition (an AICc value smaller at a difference of two units from the AICc of the OU model, simulated with alpha = 1). The dashed line indicates the 5% value. Positive values for the r parameter in diversity-dependent models and S parameter in the matching competition model are indicated by a dark gray colour, whereas negative values of the parameters are indicated by light gray colours. Results summarized across trees with n = a) 25, b) 51, c) 112, and d) 195 species.

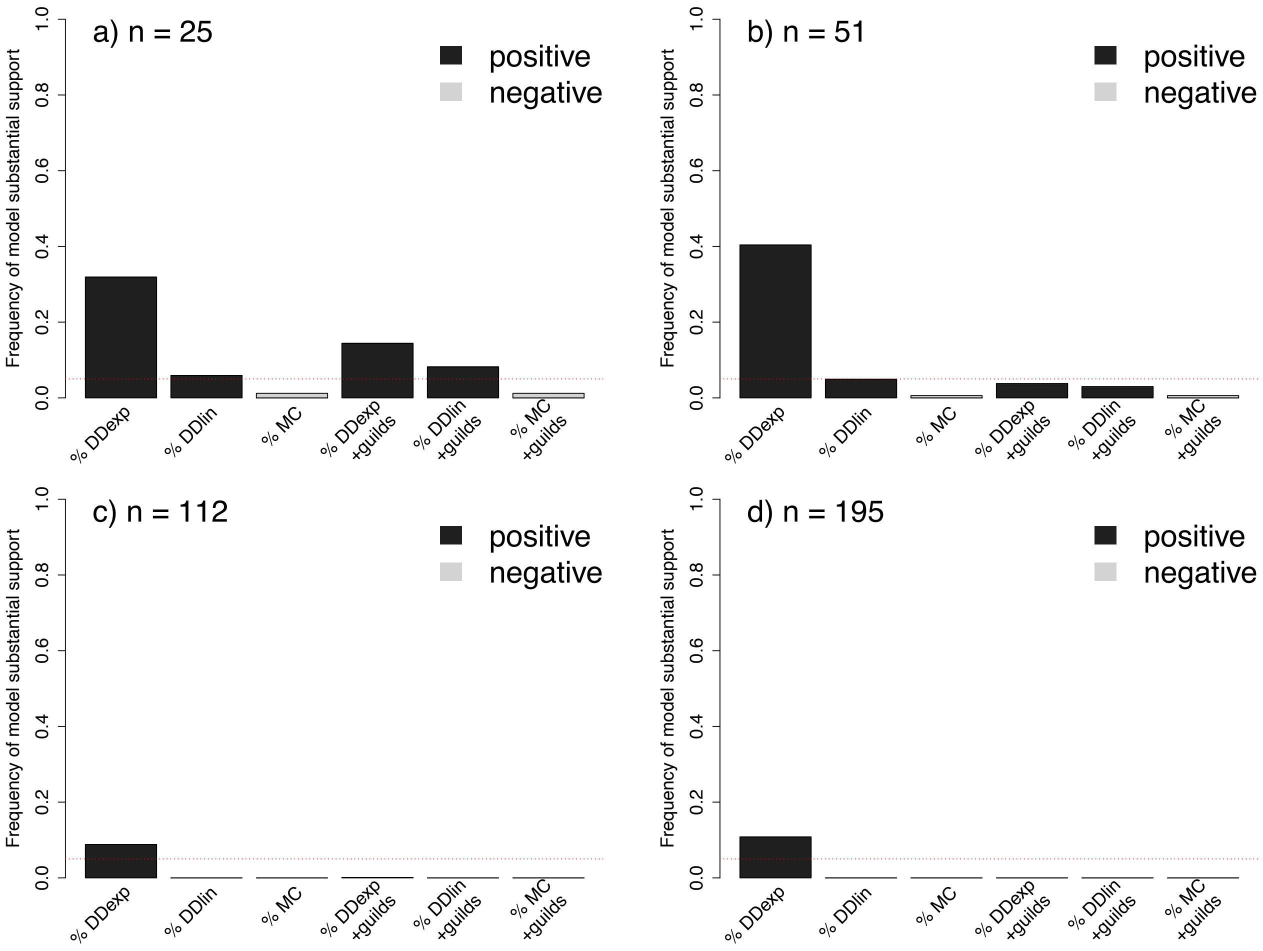

Figure S7. False positives error quantified as substantial support for models with competition (an AICc value smaller at a difference of two units from the AICc of the OU model, simulated with alpha = 5). The dashed line indicates the 5% value. Positive values for the r parameter in diversity-dependent models and S parameter in the matching competition model are indicated by a dark gray colour, whereas negative values of the parameters are indicated by light gray colours. Results summarized across trees with n = a) 25, b) 51, c) 112, and d) 195 species.

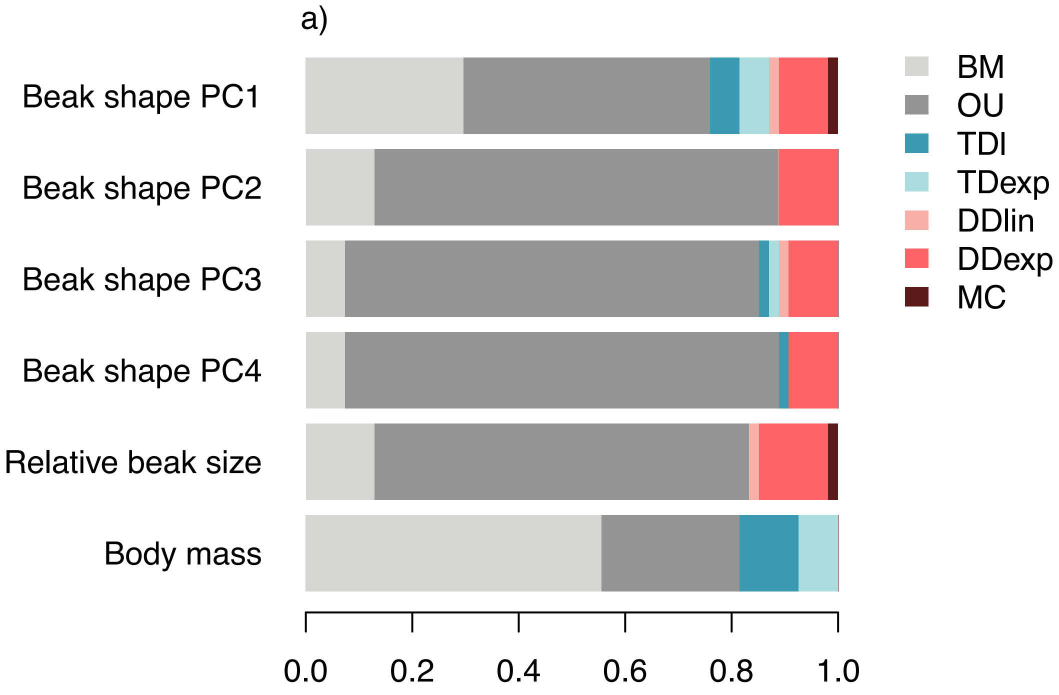

Figure S8. a) Model support (proportion of times each model is chosen as best i.e. smallest AICc values) across clades when modelling the evolution of various ecomorphological traits. We consider models without competition: Brownian motion (BM), Ornstein-Uhlenbeck (OU), linear (TDlin) and exponential (TDexp) time-dependent, and models with competition: linear (DDlin) and exponential (DDexp) diversity-dependent, and the matching competition (MC). Results summarized across avian orders and super-families trees built using species for which genetic data is available (54 clades). (Overleaf) b) The signature of competition across across 54 avian orders and super-families trees built using species for which genetic data is available. Support for competition (purple) is determined by whether a diversity or trait dependent model explains the data best in the focal clade, with an AICc difference greater than two compared to any model assuming lineages evolve independently. The following ecomorphological traits are considered (left to right circles): beak shape (purple indicates signal of competition in either PC1, PC2, PC3, or PC4), relative beak size, and body mass. 16 out of 54 clades show support for competition in either beak shape, size, or body mass ("any trait" in the stacked bars). Clades where models with and without competition cannot be distinguished by an AICc difference greater than two are marked by light gray.

b)

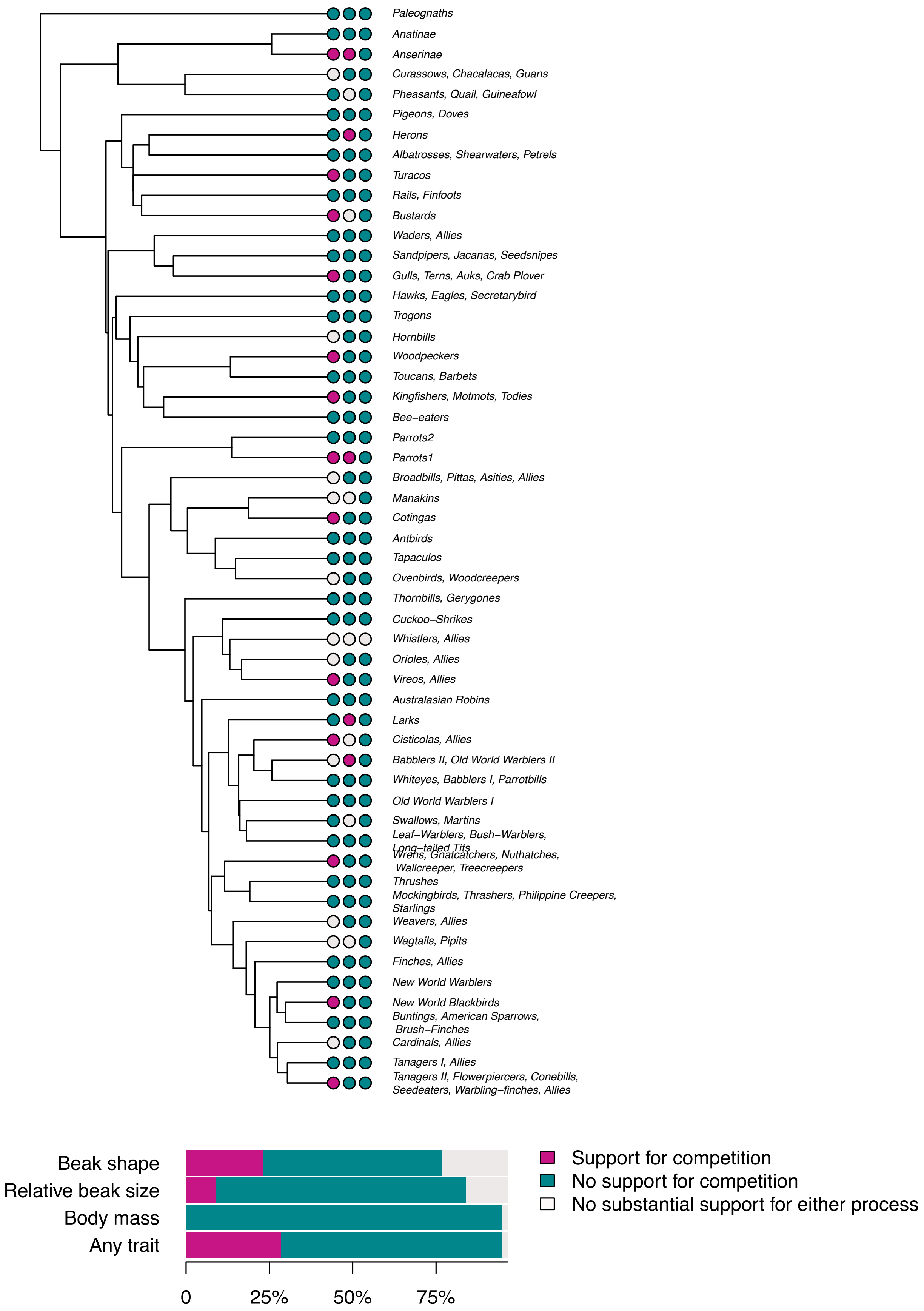
Paleognaths
Anatinae
Anserinae
Curassows, Chachalacas, Guans
Pheasants, Quail, Guineafowl
Pigeons, Doves
Herons
Albatrosses, Shearwaters, Petrels
Turacos
Rails, Finfoots
Bustards
Waders, Allies
Sandpipers, Jacanas, Seedsnipes
Gulls, Terns, Auks, Crab Plover
Hawks, Eagles, Secretarybird
Trogons
Hornbills
Woodpeckers
Toucans, Barbets
Kingfishers, Motmots, Todies
Bee–eaters
Parrots2
Parrots1
Broadbills, Pittas, Asities, Allies
Manakins
Cotingas
Antbirds
Tapaculos
Ovenbirds, Woodcreepers
Thornbills, Gerygones
Cuckoo–Shrikes
Whistlers, Allies
Orioles, Allies
Vireos, Allies
Australasian Robins
Larks
Cisticolas, Allies
Babblers II, Old World Warblers II
Whiteyes, Babblers I, Parrotbills
Old World Warblers I
Swallows, Martins
Leaf–Warblers, Bush–Warblers,
Long–tailed Tits
Wrens, Gnatcatchers, Nuthatches,
Wallcreeper, Treecreepers
Thrushes
Mockingbirds, Thrashers, Philippine Creepers,
Starlings
Weavers, Allies
Wagtails, Pipits
Finches, Allies
New World Warblers
New World Blackbirds
Buntings, American Sparrows,
Brush–Finches
Cardinals, Allies
Tanagers I, Allies
Tanagers II, Flowerpiercers, Conebills,
Seedeaters, Warbling–finches, Allies
Beak shape
Relative beak size
Body mass
Any trait
0
25%
50%
75%
Support for competition
No support for competition
No substantial support for either process

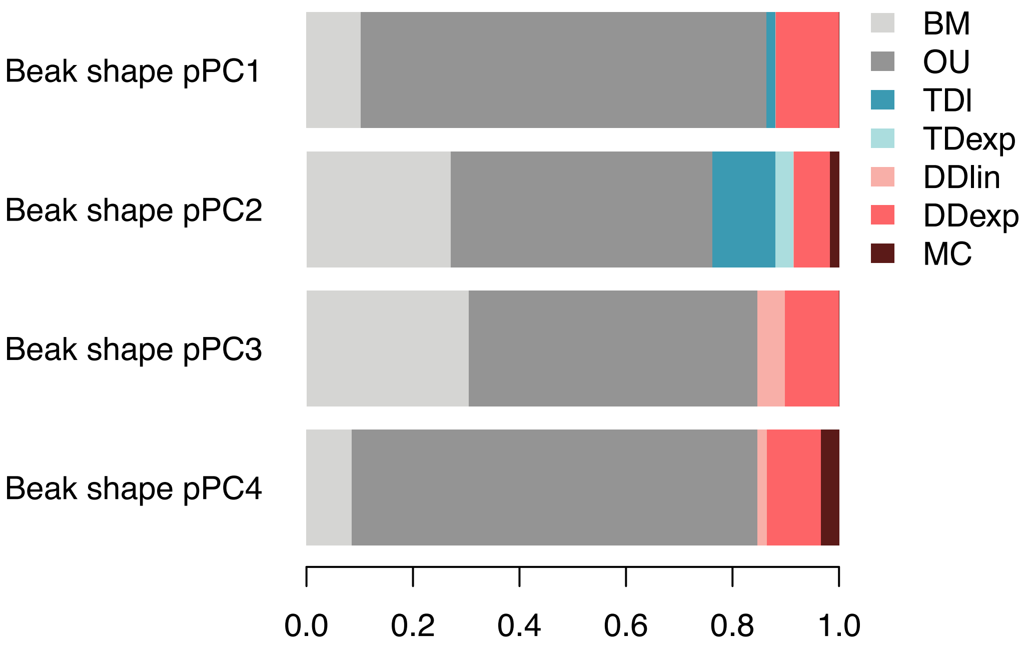

Figure S9. a) Model support (proportion of times each model is chosen as best i.e. smallest AICc values) across clades when modelling the evolution of beak shape using a phylogenetic principal component analysis. We consider models without competition: Brownian motion (BM), Ornstein-Uhlenbeck (OU), linear (TDlin) and exponential (TDexp) time-dependent, and models with competition: linear (DDlin) and exponential (DDexp) diversity-dependent, and the matching competition (MC). Results summarized across 59 avian orders and super-families. Overall, 14 out of the 59 clades show support for competition in beak shape (i.e. a diversity or trait dependent model explains the data best in the focal clade, with an AICc difference greater than two compared to any model assuming lineages evolve independently.
